# Mapping Leopard Conservation Priorities in Southeast Asia

**DOI:** 10.1002/ece3.74459

**Published:** 2026-11-02

**Authors:** Luciano Atzeni, Jan Kamler, Eric Ash, Pyae Phyoe Kyaw, Akchousanh Rasphone, Chanratana Pin, Cedric Kai Wei Tan, Susana Rostro‐García, Patrick Jantz, Ivan Gonzalez, Dawn Burnham, Samuel A. Cushman, David W. Macdonald

**Affiliations:** ^1^ Wildlife Conservation Research Unit, Department of Biology University of Oxford Oxford UK; ^2^ Wildlife Conservation Society Myanmar Program Yangon Myanmar; ^3^ World Wide Fund for Nature Vientiane Laos; ^4^ Ministry of Environment Phnom Penh Cambodia; ^5^ Conservation Ecology Program King Mongkut's University of Technology Thonburi Bangkok Thailand; ^6^ School of Biological and Environmental Sciences University of Nottingham Malaysia Semenyih Malaysia; ^7^ Panthera New York New York USA; ^8^ School of Informatics, Computing, and Cyber Systems Northern Arizona University Flagstaff Arizona USA; ^9^ Department of Biology University of Southern Denmark Odense Denmark

**Keywords:** CoLa DSS, conservation translocation, habitat connectivity, Indochinese leopard, *Panthera pardus delacouri*, reintroduction

## Abstract

The Indochinese leopard ( *Panthera pardus delacouri* ) is one of the most endangered leopard subspecies, restricted to < 4% of its historical range across Southeast Asia. With severe declines driven by poaching, habitat loss and fragmentation, and prey depletion, recovery requires urgent, spatially informed conservation action. This study presents the first connectivity analysis for the Indochinese leopard across its extant and former range, aiming to identify core habitats, priority connectivity areas and opportunities for population restoration. We used resistant kernel modelling through the CoLa Decision Support System to evaluate landscape connectivity across 14 forest complexes in the extant range and 11 potential reintroduction areas, considering scenarios of extant, reintroduction and combined range connectivity. Importance to connectivity was assessed based on Kernel Extent, Kernel Sum, movement density and protected area overlap. Our results confirmed the Dawna–Tenasserim landscape, spanning parts of Thailand and Myanmar, and Peninsular Malaysia as the most critical strongholds. In the former range, our analysis revealed potential for large‐scale metapopulation structural connectivity across Cambodia and Lao PDR, despite recent extirpations. We stress that reintroduction opportunities will require actions such as reducing poaching, restoring prey and improving habitat management. In some complexes, intensive landscape restoration may be needed to reconnect major core areas with smaller reintroduction complexes that could be critical for meta‐population connectivity. Despite increasing human pressure in the region, substantial habitat and connectivity potential remain. Our framework provides a spatial foundation to prioritise actions for preventing extinction of this subspecies and guiding large carnivore recovery across fragmented tropical landscapes.

## Graphical abstract

The Critically Endangered Indochinese leopard ( *Panthera pardus delacouri* ) is restricted to < 4% of its historical range across Southeast Asia by poaching, habitat loss and prey depletion. Using resistant kernel modelling through the CoLa Decision Support System, we present the first connectivity analysis for the subspecies across its extant and former range, evaluating 14 forest complexes in the extant range and 11 potential reintroduction areas. The Dawna–Tenasserim landscape of Thailand and Myanmar and Peninsular Malaysia emerge as the critical strongholds, while eastern Cambodia and southern Lao PDR retain the structural connectivity for large‐scale metapopulation recovery, provided poaching is reduced and prey populations restored.

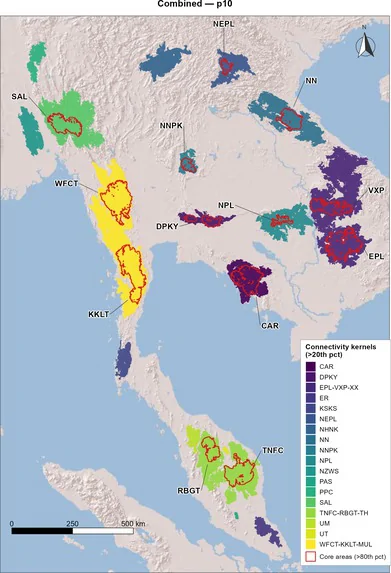

## 1 Introduction

Large carnivores have experienced dramatic global population declines and range contractions, with leopards ( *Panthera pardus* ) being particularly affected across their distribution (Stein et al. 2020; Jacobson et al. 2016). Among the subspecies, the Indochinese leopard (*P. p. delacouri*) has undergone one of the most severe declines, now persisting in only 4% of its historical range (Jacobson et al. 2016; Rostro‐García et al. 2016). This contraction mirrors the fate of other Asiatic leopard subspecies, each now occupying merely 2% of their former distributions (Jacobson et al. 2016), reflecting broader large carnivore declines across Asia (Gray et al. 2018).

The Critically Endangered Indochinese leopard has experienced a population decline > 80% over the last three generations (Rostro‐García et al. 2019). Its former range in mainland Southeast Asia was framed by a few major physiographic units: the Tenasserim range and the Dawna hills along the Thailand–Myanmar border in the west; the lowland plains of eastern Cambodia and the Annamite range along the Lao–Vietnamese border in the east; the Cardamom Mountains of southwestern Cambodia, a separate massif to the south; and the Titiwangsa range and its associated lowland forests in peninsular Malaysia. Once widespread from southern China's Pearl River to Singapore, with the Irrawaddy River in Myanmar marking its western limit (Kitchener et al. 2017), the subspecies is now extirpated in Vietnam (Rostro‐García et al. 2016; Willcox et al. 2014), Lao People's Democratic Republic (PDR), Cambodia (Rostro‐García et al. 2016, 2019, 2023; Rasphone et al. 2019) and Singapore.

This decline reflects a broader conservation crisis in Southeast Asia, a region experiencing some of the world's highest deforestation rates and biodiversity loss (Hughes 2017; Tilker et al. 2019; Gray et al. 2018). Habitat fragmentation, illegal wildlife trade, snaring for bushmeat and infrastructure development threaten the subspecies' survival (Tilker et al. 2019; Lam et al. 2023; Rostro‐García et al. 2023; Rasphone et al. 2021), with snaring emerging as the primary threat to the persistence of all large‐bodied terrestrial wildlife species in the region (O'Kelly et al. 2018; Gray et al. 2018; Ripple et al. 2016).

Currently, the Indochinese leopard persists in two major strongholds: the Northern Tenasserim Forest Complex (Thailand and Myanmar) and Peninsular Malaysia (Rostro‐García et al. 2016, 2019). In Thailand, key populations occur in the Western Forest Complex and Kaeng Krachan–Kuiburi Complex, with others in Hala Bala Wildlife Sanctuary, Salawin National Park and Namtok Huai Yang National Park along international borders (Rostro‐García et al. 2016, 2019). Myanmar hosts populations in the Southern Forest Complex extending to Lenya National Park (Rostro‐García et al. 2019), a potentially viable group in northern Karen State (Greenspan et al. 2021, 2023), and isolated populations in northern protected areas (Kyaw et al. 2021, 2024). In Malaysia, populations remain across major forest complexes (Belum–Temenggor, Taman Negara National Park, Endau–Rompin National Park) and fragmented secondary forests (Rostro‐García et al. 2016, 2019). With the total remaining Indochinese leopard population estimated to be about 622 individuals (range = 114–1130), including only about 422 mature individuals (range = 77–766; Rostro‐García et al. 2019) and recent functional extirpations in Thailand's Khlong Saeng–Khao Sok Complex (Rostro‐García et al. 2024), urgent conservation action may be needed to save this Critically Endangered subspecies.

Population assessments reveal alarmingly low densities of Indochinese leopards across their remaining range. Salak Phra Wildlife Sanctuary recorded the lowest mean (±SD) density at just 0.23 ± 0.11 leopards/100 km^2^ (Rostro‐García et al. 2024). Similarly low figures were reported in Kweekoh Wildlife Sanctuary, eastern Myanmar (0.72–1.39 leopards/100 km^2^; Greenspan et al. 2023), while Malaysian protected areas showed densities from 0.98 ± 0.55 in Pasir Raja to 2.64 ± 0.86 leopards/100 km^2^ in Taman Negara (Rostro‐García et al. 2024). These numbers reflect dramatic declines across nearly all remaining subpopulations. Before its extirpation, Cambodia's Srepok Wildlife Sanctuary population declined 82% over 10 years (2009 and 2019), including a 72% decline in just 5 years (2009–2014), primarily due to poaching (Rostro‐García et al. 2019, 2023). In Malaysia, Kenyir Wildlife Corridor experienced full population turnover and a 44% density decline between 2013 and 2015 (Hedges et al. 2015; Rimba unpubl. data; Rostro‐García et al. 2019). Even well–protected sites like Huai Kha Khaeng Wildlife Sanctuary in Thailand saw a 38% decline in just 3 years (Simcharoen and Duangchantrasiri 2008; Rostro‐García et al. 2019), indicating that no stronghold remains secure from anthropogenic pressures (e.g., Lam et al. 2023).

For large carnivores like the Indochinese leopard, landscape connectivity is crucial for population persistence (Cushman et al. 2006; Kaszta, Cushman, Htun, et al. 2020; Kaszta, Cushman, and Macdonald 2020). Apex predators require extensive ranges and traverse diverse topographic and land‐use conditions during dispersal (Fattebert et al. 2013; Elliot et al. 2014; Hussain et al. 2022). Dispersal habitat use may resemble that of resident individuals (Fattebert et al. 2015), yet other studies demonstrate movements across highly modified and fragmented landscapes, indicating that functional connectivity can persist even under strong human disturbance (Elliot et al. 2014; Wilkinson et al. 2024). At the same time, realised connectivity is often constrained by mortality risks and disturbance, even when structural corridors are available (Whittington et al. 2022; Day et al. 2020; Ash et al. 2023). Connectivity can also decline abruptly once habitat loss exceeds certain thresholds, limiting opportunities for dispersal (Cushman et al. 2010; Zemanova et al. 2017). These examples emphasise the need to distinguish structural corridors derived from models from functional connectivity shaped by anthropogenic pressures (Pitman et al. 2016; Betts et al. 2015; Modi et al. 2025; Kanagaraj et al. 2013). While structural connectivity provides a foundation, long‐term persistence ultimately depends on reducing persecution and ensuring sufficient prey and habitat quality (Bleyhl et al. 2021).

In increasingly fragmented landscapes, maintaining connectivity has become central to conservation planning (Macdonald et al. 2024; Kaszta et al. 2024; Brennan et al. 2022). Recent studies in the Indochinese leopard's range have identified critical corridors and bottlenecks affecting large felids' movements (Greenspan et al. 2021; Kaszta, Cushman, Htun, et al. 2020; Kaszta, Cushman, and Macdonald 2020; Ash et al. 2026; Suttidate et al. 2021), while broader analyses highlighted the importance of smaller habitat patches in sustaining connectivity (Diniz et al. 2021; Rezaei et al. 2022; Kanagaraj et al. 2013). Connectivity is also relevant to conservation translocations, seen as crucial tools for species recovery (Betts et al. 2015; Jule et al. 2008; Wolf and Ripple 2018; Thomas et al. 2023), which require a thorough understanding of landscape connectivity patterns to ensure long‐term success in highly fragmented landscapes (Zemanova et al. 2017; Gantchoff et al. 2022; Greenspan et al. 2021; Wilkinson et al. 2024).

Given the daunting conservation situation for the Indochinese leopard, understanding patterns of landscape connectivity becomes crucial (Cushman et al. 2018; Ash et al. 2026; Kaszta, Cushman, and Macdonald 2020). While recent research has documented population persistence in key strongholds (Rostro‐García et al. 2019, 2024; Greenspan et al. 2021, 2023), significant knowledge gaps remain regarding connectivity across the subspecies' range. Connectivity has been investigated for other large felids in Southeast Asia (Kaszta, Cushman, Htun, et al. 2020; Kaszta, Cushman, and Macdonald 2020; Greenspan et al. 2021; Suttidate et al. 2021; Ash et al. 2026; Kanagaraj et al. 2013; Modi et al. 2025), yet a comprehensive range‐wide connectivity analysis for the Indochinese leopard is still lacking.

This gap is particularly critical for identifying and evaluating prospective reintroduction areas that could establish new core populations and restore lost connectivity in historically occupied regions. Such efforts require a deep understanding of both current and potential connectivity patterns, essential for developing effective corridor networks (Kaszta, Cushman, Htun, et al. 2020; Kaszta, Cushman, and Macdonald 2020) and guiding reintroduction efforts that contribute to meta‐population recovery (Thomas et al. 2023; Jule et al. 2008; Betts et al. 2015). This analysis is urgent given Southeast Asia's rapid landscape change, which undermines opportunities for maintaining and restoring functional connectivity. To address these knowledge gaps, we present the first comprehensive connectivity analysis for the Indochinese leopard using cumulative resistant kernel modelling (Compton et al. 2007). Our approach evaluates connectivity among existing populations in extant forest complexes and assesses how potential reintroduction areas could serve as stepping stones or establish new connected strongholds. By prioritising core habitats and key corridors, we provide a systematic framework to guide urgent recovery efforts for this critically endangered subspecies.

## 2 Methods

### 2.1 Creation of the Resistance Surface

The basis for the resistance surface was a 2015–250 m land cover map of Southeast Asia (Miettinen et al. 2016). We reclassified the original layer into five categories (Table S1). This approach simplifies the thematic resolution to reduce land cover classification uncertainty and provides a conservative, transparent analogue to the resistance surface developed for Indochinese tigers ( *Panthera tigris corbetti* ) in Ash et al. (2020, 2022, 2026), thereby ensuring methodological comparability across regional large‐carnivore connectivity studies. We clipped the layer using the Indochinese leopard range polygon from Rostro‐García et al. (2019) to match the IUCN‐defined range, excluding southern China, which was absent from the original land‐cover dataset (Miettinen et al. 2016). Road features of the range countries were downloaded from OpenStreetMap (OpenStreetMap Contributors 2023). Primary and secondary roads were converted to rasters at the same resolution as the land‐cover dataset and overlaid on the reclassified resistance surface with the values of 100 and 30, respectively, using a cell statistic operation with *rule = max* (Ash et al. 2020, 2026). We reclassified the Global Human Settlement Layer (GHSL; GHS–BUILT–S–R2023A; Pesaresi and Politis 2023) into a binary raster, assigning built‐up areas a value of 100, and everything else 1. After re‐sampling to match landcover resolution, we used a cell statistic operation with *rule = max* to incorporate built‐up areas on the resistance surface (Figure 1). All operations were performed in ArcGIS Pro 3.2.2. Prior to raster and vector operations, all files were reprojected to Albers Equal Area Conic projections for Southern Asia (ESRI:102028).

**FIGURE 1:**
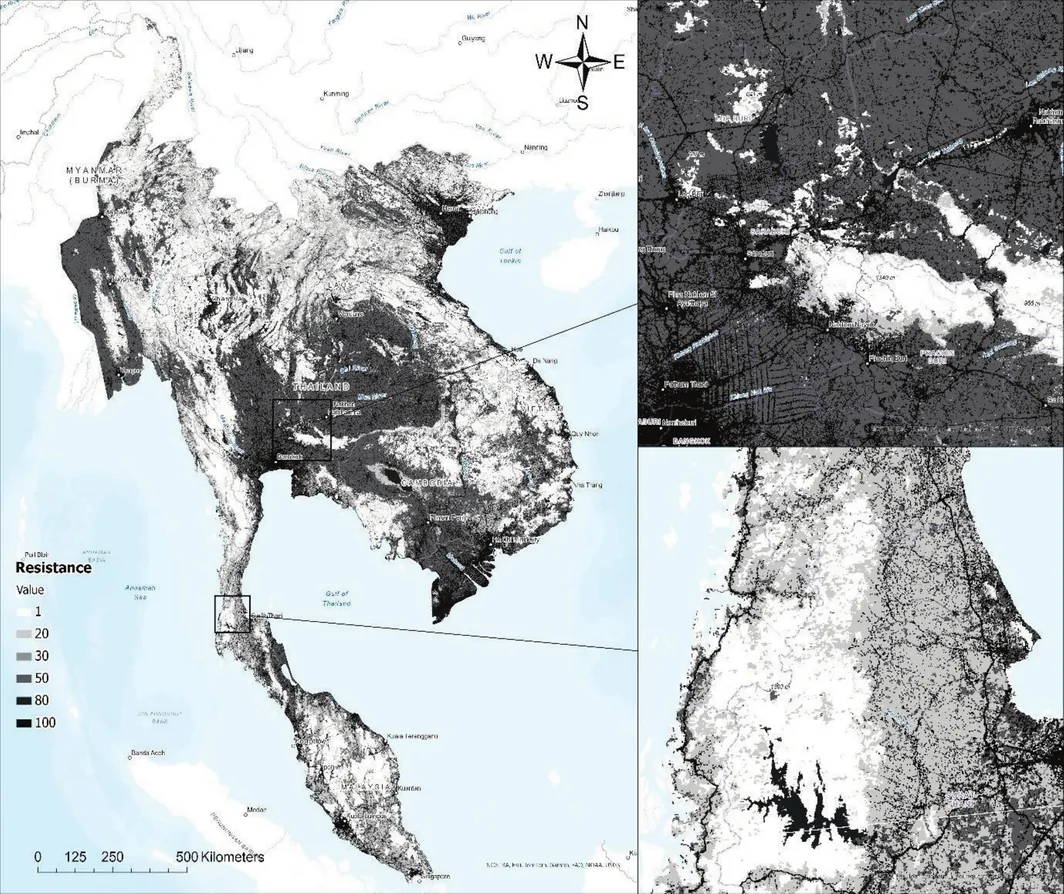
Native 250 m reclassified land‐cover resistance surface underlying the analysis; inset panels show its fine‐scale detail. The extent is clipped to the Indochinese leopard ( *Panthera pardus delacouri* ) range of Rostro‐García et al. (2019), excluding southern China.

### 2.2 Creation of Source Locations in the Extant Indochinese Leopard Range

We downloaded protected area polygon shapefiles for all countries within the Indochinese leopard's IUCN range from the WDPA (UNEP–WCMC and IUCN 2024) and merged them with Myanmar's official dataset (WCS Myanmar, unpublished; MONREC 2020, unpublished). Protected areas and forest complexes were selected as potential leopard source locations based on Rostro‐García et al. (2016, 2019) and unpublished data contributing to Stein et al. (2020) and Rostro‐García et al. (2019). In Myanmar, selections also drew on Kyaw et al. (2021, 2024) and unpublished presence data (WCS Myanmar; MONREC 2020).

After identifying all protected areas with Indochinese leopard populations, we overlaid the extant range from Stein et al. (2020) and extracted portions located outside protected areas. Protected areas and portions of extant range outside protected areas were then grouped into forest complexes using a proximity‐based clustering approach: patches within 20 km were iteratively merged until all within‐group patches were separated by no more than 20 km. This threshold was based on documented leopard dispersal capabilities, as individuals typically disperse long distances at maturity (Fattebert et al. 2013) and can cross fragmented, human‐dominated landscapes (Wilkinson et al. 2024). The 20 km threshold represents a precautionary threshold, remaining far below the largest reported dispersal distances (~194.5 km, Fattebert et al. 2013; ~111 km, Liang et al. 2022), supporting the aggregation of patches into structurally connected forest complexes.

This grouping criterion generated the following starting localities (Table 1; Figure 2): The Western Forest Complex‐Taninthayi (WFCT) spans across both Thailand and Myanmar, and connects several protected areas, including Thung Yai Naresuan Wildlife Sanctuary, Huai Kha Khaeng Wildlife Sanctuary, Salak Phra Wildlife Sanctuary, Khuen Si Nakarin National Park and Khao Laem National Park in Thailand with the Taninthayi Nature Reserve in Myanmar. Further south, the Kaeng Krachan Forest Complex‐Lenya‐Taninthayi (KKLT) links Namtok Huay Yang National Park, Kuiburi National Park and Kaeng Krachan National Park Forest Complex in Thailand with Myanmar's Lenya National Park system, including its extension and Taninthayi National Park. The Salawin complex (SAL) in Thailand comprises Salawin National Park and its associated Wildlife Sanctuary. In Malaysia, the Taman Negara Forest Complex (TNFC) incorporates several protected areas, including Jerangau Forest Reserve, Jengai Forest Reserve, Hulu Nerus Forest Reserve, and Taman Negara across Pahang, Kelantan, and Terengganu states, along with Sungai Ketiar Wildlife Sanctuary. The Royal Belum–Gunung–Temenggor complex (RBGT) includes Royal Belum State Park and Gunung Basor Forest Reserve in Malaysia, connecting with Hala Bala Wildlife Sanctuary in Thailand. We included previously identified extant populations in Malaysia, including Ulu Muda Forest Reserve (UM), Tengku Hassanal Wildlife Reserve (TH; formerly Krau Wildlife Reserve), Endau–Rompin complex (ER), Pasoh Forest Reserve (PAS), and Ayer Hitam Forest Reserve (AH). We also considered several smaller but significant protected areas, including the Mulayit Wildlife Sanctuary (MUL), North Zarmayi Wildlife Sanctuary (NZWS), and Panlaung Pyadalin Cave Wildlife Sanctuary (PPC) in Myanmar (Table 1; Figure 2).

**TABLE 1:** Forest complexes in the extant range of the Indochinese leopard. Sites (Field ID) represent either forest complexes or single protected areas.

ID	Protected areas	Country	Area	Points
WFCT	Taninthayi Nature Reserve	MMR	13,701.53	210
	Thung Yai Naresuan Wildlife Sanctuary	THA		
	Huai Kha Khaeng Wildlife Sanctuary	THA		
	Salak Phra Wildlife Sanctuary	THA		
	Khuen Si Nakarin National Park	THA		
	Khao Laem National Park	THA		
KKLT	Taninthayi National Park	MMR	12,544.56	192
	Lenya National Park	MMR		
	Lenya National Park (Extension)	MMR		
	Namtok Huai Yang National Park	THA		
	Kuiburi National Park	THA		
	Kaeng Krachan National Park Forest Complex	THA		
SAL	Salawin National Park	THA	5990.39	92
	Salawin Wildlife Sanctuary	THA		
TNFC	Jengai Forest Reserve	MYS	5981.31	92
	Jerangau Forest Reserve	MYS		
	Hulu Nerus Forest Reserve	MYS		
	Taman Negara National Park (Pahang)	MYS		
	Taman Negara National Park (Kelantan)	MYS		
	Taman Negara National Park (Terengganu)	MYS		
	Sungai Ketiar Wildlife Sanctuary	MYS		
RBGT	Hala Bala Wildlife Sanctuary	THA	3684.91	56
	Royal Belum State Park	MYS		
	Gunung Basor Forest Reserve	MYS		
MUL	Mulayit Wildlife Sanctuary	MMR	2114.95	32
UM	Ulu Muda Forest Reserve	MYS	1256.86	19
NZWS	North Zarmayi Wildlife Sanctuary	MMR	998.163	15
ER	Endau Rompin (Johor) National Park	MYS	910.76	14
	Endau Rompin (Pahang) State Park	MYS		
TH	Tengku Hassanal Wildlife Reserve	MYS	635.79	10
PPC	Panlaung Pyadalin Cave Wildlife Sanctuary	MMR	364.32	6
UT	Ulu Temiang Forest Reserve	MYS	152.13	2
PAS	Pasoh Forest Reserve	MYS	136.30	2
AH	Ayer Hitam Forest Reserve	MYS	16.78	1

*Note:* Area (in km^2^) refers to the cumulative sum of the extent of protected areas plus any polygon classified as extant in Rostro‐García et al. (2019) and considered part of a complex. Points were initialised based on a simple relationship between area and the average density (1.532 individuals/100 km^2^), calculated for the Indochinese leopard by Rostro‐García et al. (2024). Country labels: MMR, Myanmar; MYS, Malaysia; THA, Thailand.

Abbreviations: AH, Ayer Hitam; ER, Endau Rompin complex; KKLT, Kaeng Krachan Forest Complex‐Lenya National Park–Taninthayi National Park; MUL, Mulayit complex; NZWS, North Zarmayi Wildlife Sanctuary; PAS, Pasoh complex; PPC, Panlaung Pyadalin Cave Wildlife Sanctuary; RBGT, Royal Belum–Gunung–Temenggor complex; SAL, Salawin complex; TH, Tengku Hassanal Wildlife Reserve; TNFC, Taman Negara Forest Complex; UM, Ulu Muda; UT, Ulu Temiang; WFCT, Western Forest Complex‐Taninthayi Nature Reserve.

**FIGURE 2:**
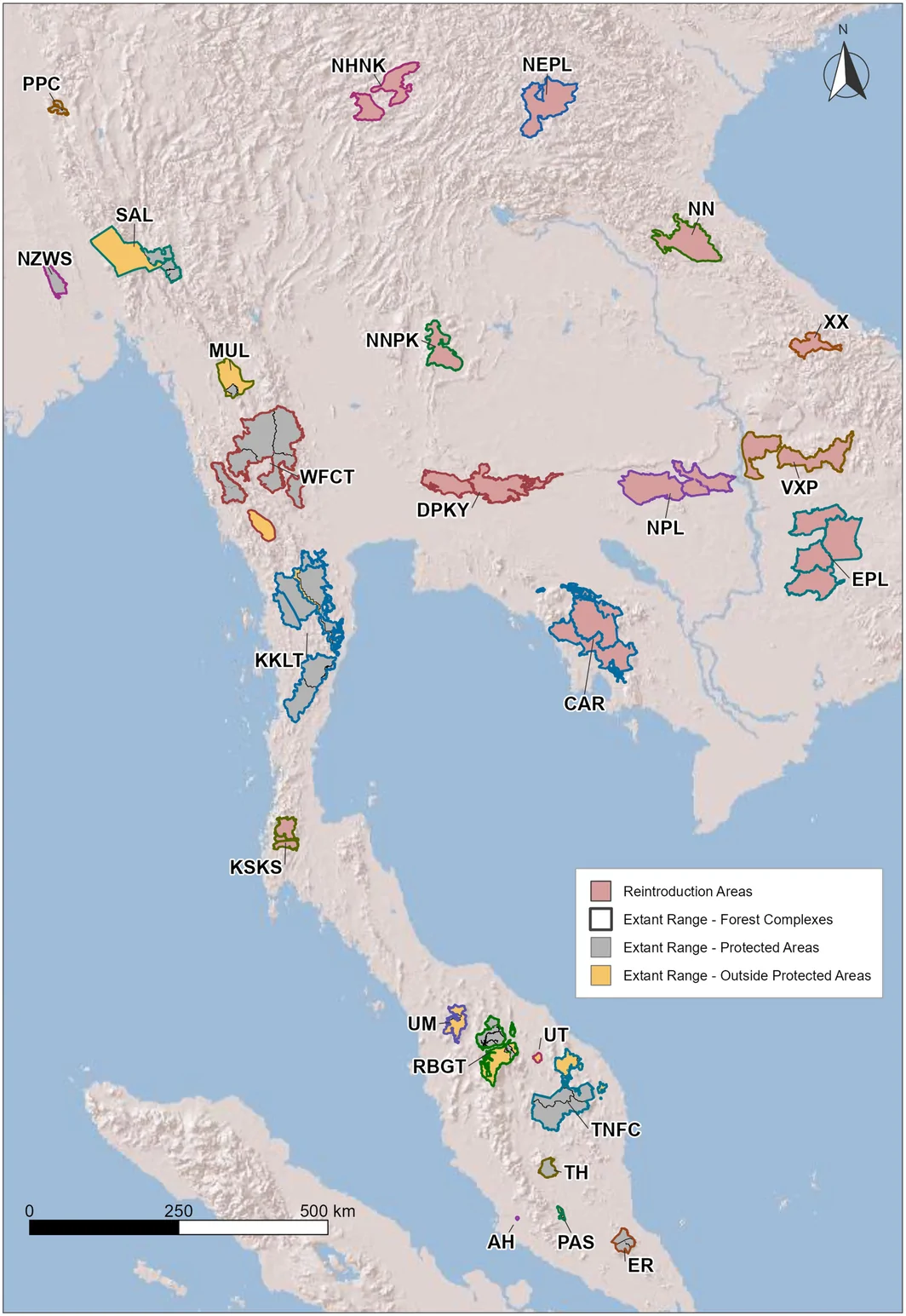
Extant range and proposed reintroduction areas of the Indochinese leopard ( *Panthera pardus delacouri* ) across Southeast Asia. Reintroduction areas are shown in pink; within the extant range, protected areas are shown in grey and land outside protected areas in orange, while each forest complex is outlined in its own border colour to distinguish it from neighbouring complexes (see Tables 1 and 2 for further details on the forest complexes). Extant Range: WFCT = Western Forest Complex–Taninthayi Nature Reserve; KKLT = Kaeng Krachan Forest Complex–Lenya National Park–Taninthayi National Park; SAL = Salawin complex; TNFC = Taman Negara Forest Complex; RBGT = Royal Belum–Gunung–Temenggor complex; MUL = Mulayit complex; UM = Ulu Muda; NZWS = North Zarmayi Wildlife Sanctuary; ER = Endau Rompin complex; TH = Tengku Hassanal Wildlife Reserve; PPC = Panlaung Pyadalin Cave Wildlife Sanctuary; UT = Ulu Temiang; PAS = Pasoh complex; AH = Ayer Hitam. Reintroduction areas: KSKS = Khlong Saeng–Khao Sok Complex; CAR = Cardamom Mountains; DPKY = Dong Phayayen–Khao Yai Forest Complex; NNPK = Nam Nao–Phu Khiew complex; NPL = Northern Plains Landscape; VXP = Virachey–Xe Pian complex; EPL = Eastern Plains Landscape; NHNK = Nam Ha–Nam Kan complex; NN = Nakai–Nam Theun; NEPL = Nam Et–Phou Louey; XX = Xe Xap.

The number of source points per area (Table 1), which serve as starting locations for the cumulative resistant kernel model, was calculated by multiplying the average mean density taken from six study areas across four countries in mainland Southeast Asia (1.532 individuals/100 km^2^; Rostro‐García et al. 2024) by the area of each forest complex (in km^2^), then dividing by 100 (see Rostro‐García et al. 2024 for the SD and 2.5th and 97.5th percentiles for each density estimate at the six sites). To allow variability in the locations of the source points, we created 10 sets of random source points using ArcGIS Pro 3.2.2.

### 2.3 Creation of Source Locations in Proposed Reintroduction Areas in the Indochinese Leopard Former Range

We hypothesised 11 potential reintroduction locations based on recent local extinction times (Rostro‐García et al. 2016, 2023, 2024; Stein et al. 2020) (Table 2; Figure 2). These areas extend across Thailand, Cambodia and Lao PDR. In Thailand, we identified the Khlong Saeng Wildlife Sanctuary–Khao Sok National Park complex (KSKS), the Dong Phayayen–Khao Yai Forest Complex (DPKY) and the Nam Nao National Park–Phu Khiew Wildlife Sanctuary complex (NNPK). In Cambodia, the Cardamom Mountains complex (CAR) encompasses the Cardamom Corridor, Central Kravanh National Park and Southern Kravanh National Park regions. The Northern Plains Landscape (NPL) includes three interconnected areas: Chhep Wildlife Sanctuary, Kulen Promtep Wildlife Sanctuary and Preah Roka Wildlife Sanctuary. The Eastern Plains Landscape (EPL) comprises Keo Seima Wildlife Sanctuary, Lumphat Wildlife Sanctuary, Phnom Prich Wildlife Sanctuary and Srepok Wildlife Sanctuary. The Virachey National Park–Xe Pian National Protected Area complex (VXP) uniquely spans both Cambodia and Lao PDR. In Lao PDR, we identified several additional sites: the Nam Ha National Protected Area–Nam Kan National Park complex (NHNK), the Nakai–Nam Theun National Park area (NN), Nam Et–Phou Louey National Park (NEPL) and Xe Xap National Protected Area (XX). The number of points initialised in these complexes followed the same rationale adopted for the extant range (Table 2). As with extant locations, we introduced stochasticity in the modelling by creating 10 sets of random source points using ArcGIS Pro 3.2.2.

**TABLE 2:** Proposed reintroduction areas in the Indochinese leopard range. Sites (Field ID) represent either forest complexes or single protected areas.

ID	Protected areas	Country	Area	Points
KSKS	Khao Sok National Park	THA	1629.70	24
	Khlong Saeng Wildlife Sanctuary	THA		
CAR	Cardamom Corridor	KHM	9848.47	150
	Central Kravanh National Park	KHM		
	Southern Kravanh National Park	KHM		
DPKY	Dong Phayayen–Khao Yai Forest Complex	THA	6218.58	95
NNPK	Nam Nao National Park	THA	2558.70	39
	Phu Khiew Wildlife Sanctuary	THA		
NPL	Chhep Wildlife Sanctuary	KHM	6930.68	105
	Kulen Promtep Wildlife Sanctuary	KHM		
	Preah Roka Wildlife Sanctuary	KHM		
VXP	Virachey National Park	KHM	5931.19	90
	Xe Pian National Protected Area	LAO		
EPL	Keo Seima Wildlife Sanctuary	KHM	11,410.89	173
	Lumphat Wildlife Sanctuary	KHM		
	Phnom Prich Wildlife Sanctuary	KHM		
	Srepok Wildlife Sanctuary	KHM		
NHNK	Nam Ha National Protected Area	LAO	3671.08	56
	Nam Kan National Park	LAO		
NN	Nakai–Nam Theun National Park	LAO	4828.46	65
NEPL	Nam Et–Phou Louey National Park	LAO	4256.15	74
XX	Xe Xap National Protected Area	LAO	1538.41	23

*Note:* Area (in km^2^) refers to the cumulative sum of the protected areas extent. Points were initialised based on a simple relationship between area and the average density (1.532 individuals/100 km^2^), calculated for the Indochinese leopard by Rostro‐García et al. (2024). Country labels: KHM, Cambodia; LAO, Lao PDR; THA, Thailand.

Abbreviations: CAR, Cardamom Mountains; DPKY, Dong Phayayen–Khao Yai Forest Complex; EPL, Eastern Plains Landscape; KSKS, Khlong Saeng–Khao Sok Complex; NEPL, Nam Et–Phou Louey; NHNK, Nam Ha–Nam Kan complex; NN, Nakai–Nam Theun; NNPK, Nam Nao–Phu Khiew complex; NPL, Northern Plains Landscape; VXP, Virachey–Xe Pian complex; XX, Xe Xap.

### 2.4 Connectivity Model

We used the software CoLa DSS (Connecting Landscapes Decision Support System) implemented as an R package (Jantz et al. 2025), to run resistant kernel models (Compton et al. 2007). Cumulative resistant kernels provide spatially comprehensive and omnidirectional assessments of connectivity patterns. The cumulative resistant kernel approach estimates expected movement density patterns from source locations to all other cells across the raster landscape and has been shown to outperform many other methods in estimating actual movement patterns (Cushman et al. 2014), gene flow (Atzeni et al. 2024) and simulated movement density (Lumia et al. 2024). The cumulative resistant kernel method calculates the probability that organisms originating from source points can reach specific cells within user‐defined distance parameters based on the landscape's resistance, the density and placement of source points, and the organism's dispersal capability. The software is a wrapper and extension of pre‐existing tools for modelling landscape connectivity (UNICOR; Landguth et al. 2012) and landscape genetics (CDPOP; Landguth and Cushman 2010), including added functionalities for the creation and modification of resistance surfaces.

The maximum dispersal ability was set at 200 km. This threshold was informed by the longest documented straight‐line leopard dispersal (~194.5 km for African leopards, Fattebert et al. 2013), that serves as a conservative upper bound rather than a typical value. Asia‐based estimates for the North China leopard (*P. p. japonensis*) indicate dispersal potential of ~111 km (Liang et al. 2022), underscoring that the 200 km value used in this analysis is a cautious ceiling rather than an assumption of routine movements. Dispersal probability declined with distance following a Gaussian (half‐normal) kernel, *K* (*d*) = exp (−*d* ^2^/2*σ* ^2^), with the standard deviation fixed at one quarter of the maximum dispersal distance (σ = 50 km), so that the 200 km threshold corresponds to 4*σ*. The kernel equalled 1 at the source, declined smoothly with cumulative resistance distance, and was set to zero beyond 200 km.

We simulated cumulative resistant kernels (function *cola::crk_py()*) under two source‐point scenarios: one using source points in the extant range (Extant Range scenario) and one using source points in the hypothesised reintroduction areas (Reintroduction scenario). To account for the stochastic placement of source points, each scenario was run with 10 independent sets of random source points per study area, and the 10 resulting kernel surfaces were averaged to produce a single connectivity surface per study area. These per‐area surfaces were then combined by taking the cell‐wise maximum across the areas of a scenario, yielding a landscape connectivity surface for the extant range and one for the reintroduction areas; a third, Combined surface was obtained as the cell‐wise maximum of the two, representing connectivity under a scenario in which reintroductions have been implemented alongside the extant population.

### 2.5 Sensitivity to the Resistance Transform and Pixel Grain

We assessed the sensitivity of the connectivity surfaces to two analytical choices: the functional form of the resistance surface (hereafter transform) and the pixel grain at which connectivity was modelled. These two axes were chosen because they act on every cell of the surface and are the choices most likely to bias a resistance‐based connectivity model: the functional form determines how strongly the modified matrix is penalised relative to forest, which is the assumption most exposed when resistance values are transferred between species, and the grain determines whether linear barriers are diluted by aggregation. The number of source points was not varied, since it follows deterministically from the area of each complex and a single published density estimate; its stochastic component, the placement of the points, was instead addressed with the 10 replicate sets described above. An application of the same framework to tigers in the region, in which connectivity was simulated under low, medium and high population sizes for every landscape, found the resulting priorities to be largely insensitive to the starting population size (Ash et al. 2026).

Alternative resistance surfaces were derived from the baseline surface described above by varying the functional form of resistance rather than reassigning individual class values. We applied a monotonic power transform to the baseline values and rescaled the result to 1–100, using the formula

$$R^{\prime }=1+\left(R^{p}-1\right)/\left(100^{p}-1\right)\times 99.$$

Because the transform is strictly monotonic and fixes the endpoints, it leaves the extreme classes unchanged (forest = 1; urban, built‐up and primary roads = 100) and re‐weights only the intermediate human‐influenced and non‐forest classes, while guaranteeing that every distinct input class maps to a distinct output value. Varying the exponent produced three surfaces differing only in form: concave (*p* = 0.7), which raises the resistance of the intermediate classes, the identity (*p* = 1.0; our baseline) and convex (*p* = 1.5), which lowers them (Table S2) [note that the same exponents are labelled with the opposite terms in Ash et al. (2020)]. These bracket a sharper versus a softer penalty for non‐forest habitat and follow the power‐transform approach previously used to represent alternative hypotheses about the relative resistance of land‐cover classes in the regional tiger surface (Ash et al. 2020). Grain was assessed by running the full connectivity analysis on two grids obtained from the native ~250 m resistance layer by block averaging: a ~1 km grid (aggregation factor 4), used throughout the study, and a finer ~500 m grid (aggregation factor 2).

For both axes the comparison was made on the individual, per‐replicate kernel surfaces, that is, one raster per study area, scenario and replicate, before any averaging across replicates or combination into landscape surfaces. In this way, the agreement was measured on the raw model output rather than on smoothed products. Each pair of surfaces was compared over their union footprint, defined as the cells with a value > 0 in at least one of the two surfaces, and three coefficients were computed: Pearson's *r*, Spearman's *ρ* and Pearson's *r* on log (*x* + 1)—transformed values, the last to accommodate the heavy‐tailed distribution of movement density, while accommodating the cells that are zero in one of the two surfaces. Along the resistance axis we compared the three transforms pairwise (p0.7 vs. p1.0, p1.0 vs. p1.5, p0.7 vs. p1.5) on the ~1 km grid, holding study area, scenario and replicate constant. Along the grain axis, we compared the ~1 km and ~500 m surfaces of the same resistance transform at both grains: at the coarse grain, by block‐aggregating the ~500 m surface by a factor of two onto the ~1 km grid, and at the fine grain, by block‐replicating the ~1 km surface by a factor of two onto the ~500 m grid; the coarse grain measures agreement at the resolution of the reported results, while the fine grain additionally reflects that the coarser surface carries no sub‐kilometre structure by construction. Correlations were computed for every study area × scenario × replicate combination and summarised in two ways: as the median, minimum and maximum of each coefficient across all comparisons within a comparison axis (Section S1), and as the mean Spearman's *ρ* (±SD across the 10 replicates) per study area, reported separately for the extant‐range and reintroduction scenarios (Table 3).

**TABLE 3:** Agreement of the cumulative resistant‐kernel connectivity surfaces between analytical choices, per study area.

Scenario	Study area	Resistance			Grain	
		p0.7 versus p1.0	p1.0 versus p1.5	p0.7 versus p1.5	1 km versus 500 m (1 km)	1 km versus 500 m (500 m)
Extant	AH	0.998 ± 0.000	0.997 ± 0.001	0.993 ± 0.001	0.966 ± 0.024	0.918 ± 0.029
Extant	ER	0.993 ± 0.000	0.966 ± 0.002	0.937 ± 0.003	0.993 ± 0.001	0.991 ± 0.001
Extant	KKLT	0.993 ± 0.000	0.982 ± 0.001	0.959 ± 0.001	0.988 ± 0.002	0.985 ± 0.002
Extant	MUL	0.985 ± 0.002	0.977 ± 0.002	0.936 ± 0.005	0.952 ± 0.025	0.949 ± 0.026
Extant	NZWS	0.998 ± 0.000	0.994 ± 0.001	0.985 ± 0.002	0.982 ± 0.004	0.978 ± 0.005
Extant	PAS	0.986 ± 0.001	0.812 ± 0.011	0.727 ± 0.015	0.965 ± 0.041	0.961 ± 0.036
Extant	PPC	0.980 ± 0.003	0.957 ± 0.005	0.909 ± 0.008	0.930 ± 0.024	0.927 ± 0.025
Extant	RBGT	0.989 ± 0.001	0.957 ± 0.005	0.923 ± 0.004	0.990 ± 0.001	0.988 ± 0.001
Extant	SAL	0.987 ± 0.001	0.973 ± 0.001	0.936 ± 0.002	0.966 ± 0.011	0.965 ± 0.011
Extant	TH	0.996 ± 0.000	0.970 ± 0.003	0.940 ± 0.004	0.982 ± 0.019	0.981 ± 0.016
Extant	TNFC	0.997 ± 0.000	0.988 ± 0.000	0.975 ± 0.001	0.991 ± 0.001	0.989 ± 0.002
Extant	UM	0.987 ± 0.001	0.968 ± 0.002	0.920 ± 0.003	0.965 ± 0.013	0.963 ± 0.014
Extant	UT	0.966 ± 0.044	0.936 ± 0.009	0.828 ± 0.076	0.945 ± 0.052	0.937 ± 0.058
Extant	WFCT	0.994 ± 0.000	0.985 ± 0.001	0.965 ± 0.002	0.986 ± 0.004	0.984 ± 0.004
Reintroduction	CAR	0.997 ± 0.000	0.994 ± 0.000	0.984 ± 0.001	0.984 ± 0.005	0.978 ± 0.006
Reintroduction	DPKY	0.997 ± 0.001	0.993 ± 0.001	0.982 ± 0.002	0.986 ± 0.004	0.981 ± 0.005
Reintroduction	EPL	0.997 ± 0.000	0.994 ± 0.000	0.985 ± 0.001	0.978 ± 0.004	0.975 ± 0.005
Reintroduction	KSKS	0.996 ± 0.000	0.991 ± 0.001	0.977 ± 0.001	0.988 ± 0.002	0.979 ± 0.002
Reintroduction	NEPL	0.989 ± 0.002	0.965 ± 0.003	0.926 ± 0.004	0.970 ± 0.005	0.970 ± 0.007
Reintroduction	NHNK	0.986 ± 0.002	0.971 ± 0.002	0.924 ± 0.003	0.983 ± 0.007	0.979 ± 0.007
Reintroduction	NN	0.993 ± 0.000	0.983 ± 0.000	0.958 ± 0.001	0.968 ± 0.003	0.965 ± 0.004
Reintroduction	NNPK	0.997 ± 0.000	0.990 ± 0.001	0.976 ± 0.002	0.990 ± 0.002	0.988 ± 0.002
Reintroduction	NPL	0.995 ± 0.001	0.989 ± 0.002	0.972 ± 0.005	0.970 ± 0.008	0.967 ± 0.009
Reintroduction	VXP	0.994 ± 0.000	0.987 ± 0.001	0.970 ± 0.002	0.977 ± 0.004	0.976 ± 0.004
Reintroduction	XX	0.991 ± 0.000	0.979 ± 0.001	0.950 ± 0.002	0.969 ± 0.005	0.967 ± 0.006

*Note:* Each cell gives the mean Spearman rank correlation ± SD across the 10 random source‐point replicates, computed between the two surfaces of a comparison over their union footprint (cells with a value > 0 in at least one surface). Comparisons are grouped along two axes. Resistance: The three functional forms of the resistance surface compared pairwise (p0.7 concave vs. p1.0 linear; p1.0 vs. p1.5 convex; p0.7 vs. p1.5, the most divergent pair), all at the ~1 km grain. Grain: The ~1 km and ~500 m surfaces of the same resistance transform, reported at both grains, that is with the ~500 m surface aggregated onto the ~1 km grid (1 km) and with the ~1 km surface replicated onto the ~500 m grid (500 m). Values close to 1 indicate that the spatial pattern of predicted movement density is essentially unchanged by the choice; lower values identify areas that are more sensitive to it. Study‐area codes are as in Table 1 (extant range) and Table 2 (reintroduction areas) or Figure 2 (both).

### 2.6 Coefficient of Variation (CoV) Across Replicates

Because source points were placed at random within each area, we quantified how reproducible the connectivity prediction was across the 10 replicate source‐point sets, holding the resistance surface fixed, so that the only source of variation was the stochastic placement of the points. For each resistance transform, we computed the per‐cell CoV as the standard deviation divided by the mean across the 10 replicate surfaces, treating missing values as zero.

CoV was computed at two spatial scopes. In the landscape scope, the study areas were first merged into a single landscape surface within each replicate by taking the cell‐wise maximum across all areas, giving 10 combined surfaces (one per replicate); the per‐cell mean, standard deviation and CoV were then computed across these 10 combined surfaces. This scope therefore measures the reproducibility of the landscape surface that is carried forward to the priority analysis. In the per‐area scope, no merging was performed: for each study area, the per‐cell CoV was computed across that area's own 10 replicate surfaces, measuring reproducibility within a single area.

CoV is undefined where no movement is predicted, so it was evaluated only over the reachable footprint, that is the cells with a replicate mean > 0. For each scope and transform we summarised the distribution of per‐cell CoV over this footprint by its median, mean and 90th percentile (describing the high‐variability tail and therefore bounding the worst case). We further indicated the percentage of footprint cells with CoV below 0.10 (highly reproducible prediction that indicates how much of the surface is well constrained), and the number of footprint cells. We also mapped the per‐cell CoV of the combined surface for the three transforms (Section S2).

We further examined whether the three resistance transforms produced spatially concordant predictions once replicate variability had been averaged out. For each transform, we correlated the replicate‐mean combined connectivity surfaces cell‐wise over their union footprint (cells with a value > 0 under at least one transform), using both Spearman's *ρ* and Pearson's *r* (Section S2).

### 2.7 Core Area and Connectivity Kernel Analysis

For each scenario, we identified Core Areas (alternatively Core Patches), defined as areas with greater than the 80th percentile of resistant kernel values. These areas represent locations where the expected density of movement is highest and define the zones that would likely support stable populations given the potential dispersal ability. Additionally, we identified Connectivity Kernels above the 20th percentile of the resistant kernel values. Unlike Core Areas, these encompass regions characterised by lower (albeit non‐negligible) intensity of movement, and represent critical areas that may facilitate dispersal processes around and between stable areas.

To create a composite kernel output across the entire Indochinese leopard range for both the Extant Range and Reintroduction scenarios, we employed a raster operation using the maximum value rule for overlapping cells. This approach was chosen over a summation rule to better represent the actual movement potential across the landscape. Using maximum values prevents the artificial inflation of movement intensity that would occur when summing values from adjacent forest complexes. This is important because movement intensity is fundamentally determined by landscape characteristics rather than the additive effect of neighbouring source areas. When two forest complexes are connected, an assessment of their combined influence on movement should reflect the underlying landscape connectivity rather than the sum of their individual effects. Finally, we created a composite scenario by overlaying the outputs from both Extant Range and Reintroduction scenarios, applying the same threshold and ranking methodology to the merged raster.

To rank these areas, we applied several key metrics, following Kaszta, Cushman, and Macdonald (2020), Cushman et al. (2018) and Ash et al. (2026). These included the total area of each patch (Kernel Extent), the sum of the kernel values within each patch (Kernel Sum), the number of protected areas intersected by, or overlapping with, the core areas or connectivity kernel patches, and the sum of the kernel values divided by the area of each patch (intensity). Intensity was included because Kernel Extent and Kernel Sum both scale with patch size, so expressing movement density per unit area distinguishes patches that concentrate high intensity of movement from those that accumulate a large total by being extensive. The relative ranking of each metric for each patch was determined as the ratio of the metric value to the highest values across all patches within the same percentile category and scenario (Ash et al. 2026). These relative rankings were then averaged to provide a final mean rank for each patch (Ash et al. 2026).

The same thresholding and ranking procedure was also conducted separately for each forest complex in both Extant Range and Reintroduction scenarios. These single‐forest‐complex outputs are provided as archived data files but are not discussed further (see Section S3), as the connectivity patterns of interest emerge between forest complexes rather than within them.

### 2.8 Robustness of the Priority Ranking

To determine whether the identification of the highest priority areas was robust to the resistance parameterisation, we compared the priority rankings obtained under the three resistance transforms in the Combined scenarios. The ranked patches are not directly comparable between transforms because a patch may merge several forest complexes under one transform and keep them separate under another. We therefore mapped the ranking onto a fixed and always comparable set of units, the forest complexes: each patch was expanded into its constituent complexes, and every complex was assigned the composite score (mean rank) of the patch it belongs to. Where a complex belonged to more than one patch, because it was fragmented or because a cluster was split under another transform, it retained its best patch, that is, the highest composite score and the lowest patch rank; a complex falling in no patch was assigned a score of zero and ranked last. The set of complexes was taken as the union of the constituents across the three transforms, so that merged complexes share the score of their cluster and no complex is dropped.

On this fixed set of complexes, and separately for each scenario and percentile threshold, we quantified concordance among the three rankings with Kendall's coefficient of concordance (*W*), corrected for ties and tested against a *χ* ^2^ distribution, and with the Spearman rank correlation averaged over the three pairs of transforms. Values of *W* approaching 1 indicate that the three transforms order the complexes in the same way. We additionally summarised the stability of each individual complex as the mean, standard deviation and range (minimum to maximum) of the rank it held across the three transforms, so that complexes whose priority was invariant could be distinguished from those whose position shifted (Section S4).

Because the coalescence of connectivity kernels is itself a source of rank movement, we further examined the complexes composed of more than one forest area, that is, the clusters that form when connectivity kernels merge, in the Combined scenario at the Connectivity Kernel threshold. For each such cluster, we followed all four metrics across the three transforms, in order of increasing landscape permeability, together with the pairwise percentage change between transforms, noting, for the areas that join later, the transform at which they enter the cluster.

### 2.9 Sensitivity of the Priority Metrics and Connectivity Differences

Robustness quantifies whether the transforms order the areas in the same way, but not whether the metrics themselves take the same values; therefore we additionally examined how much each metric changed between transforms. Because a set of forest areas can form a single merged patch under one transform and several separate patches under another, a patch on one side would otherwise be compared with a whole cluster on the other. To keep every comparison like for like on a fixed unit, the areas that share a patch under either transform were grouped together and, on each side, the metric was totalled over every patch containing any of the group's areas. Kernel Extent, Kernel Sum and the number of protected areas are additive and were summed over those patches; Intensity is a ratio and cannot be summed, so it was re‐derived from the aggregated totals as the summed Kernel Sum divided by the summed extent. Because the group is defined on the union of the two transforms, an area forming a patch under only one of them contributes to that side alone.

For every group, scenario and percentile threshold we compared the three transforms pairwise and expressed the change in each metric as the symmetric relative difference, 100 × (*A*−*B*)/(*A* + *B*), which is bounded between −100 and + 100 and treats the two transforms symmetrically, so that no transform is privileged as the reference. We then averaged these differences across all groups to summarise, for each comparison, scenario and threshold, the mean sensitivity of each metric to the resistance transform. As a spatial complement, we also computed the cell‐wise absolute difference between the composite connectivity surfaces of each pair of transforms, each surface first rescaled between zero and one by its own maximum, so that disagreement could be located in space rather than only summarised by area (Section S5).

### 2.10 Software

All analyses were carried out in R 4.4.2 (R Core Team 2024) using RStudio 2025.05.0 (Posit team 2025). Cumulative resistant kernels were generated with the cola package (Jantz et al. 2025). The raster, vector and dataframe operations through which the Core Areas and Connectivity Kernels were delineated, and all subsequent spatial processing, used terra (Hijmans 2025) and sf (Pebesma 2018; Pebesma and Bivand 2023). Figures were produced with ggplot2 (Wickham 2016), using tidyterra (Hernangómez 2023) to render raster layers, ggspatial (Dunnington 2023), maptiles (Giraud 2025) and ggrepel (Slowikowski 2024) for the map frames, basemaps and leader labelled annotations, patchwork (Pedersen 2024) for panel composition, and viridisLite (Garnier et al. 2023) and scales (Wickham et al. 2025) for colour and axis scaling. Formatted tables were produced with flextable (Gohel and Skintzos 2024) and officer (Gohel et al. 2026).

## 3 Results

### 3.1 Sensitivity to the Resistance Transform and Grain Size

The connectivity surfaces were highly concordant across all analytical choices. Pooled over all study areas and replicates, the median Spearman correlation was 0.993 between the two most similar transforms (p0.7 vs. p1.0) and 0.982 between p1.0 and p1.5, and remained high even between the two most divergent forms, p0.7 vs. p1.5 (0.958) (Table S3). Along the grain axis the median Spearman correlation was 0.980 at the ~1 km grain and 0.977 at the ~500 m grain, with the same pattern for the Pearson and log‐transformed coefficients (Table S3). Agreement was equally high within each scenario, with a global mean Spearman correlation of 0.962 across the extant forest complexes (Figure S1) and 0.979 across the reintroduction areas (Figure S2), and it was consistent across individual complexes (Table 3): per‐area mean correlations exceeded 0.95 in every area for p0.7 versus p1.0, and the few lower values were confined to the smallest complexes, in particular PAS (0.727) and UT (0.828) for the most divergent transform pair, and PPC (0.930) and AH (0.918) for the grain comparison.

### 3.2 Coefficient of Variation

At the landscape scale, the median per‐cell CoV of the combined surface was low and almost identical under the three transforms (0.180, 0.174 and 0.165 for p0.7, p1.0 and p1.5), as were the corresponding means (0.347, 0.316 and 0.292). The proportion of the reachable footprint with a CoV below 0.10 rose from 22.0% to 23.0% and 25.3% across the same sequence, while the 90th percentile of the per‐cell CoV fell from 0.714 to 0.587 and 0.513 (Table S4).

Within the extant range, TH, ER and NZWS were the most reproducible complexes under every transform, with median per‐cell CoV between 0.055 and 0.125, mean CoV between 0.134 and 0.284, and 41.4% to 64.3% of their cells below a CoV of 0.10. The least reproducible were UT, AH and PAS, where median CoV ranged from 0.305 to 2.062 and mean CoV from 0.551 to 2.088, the 90th percentile reached 1.104 to 3.162, and virtually no cell fell below 0.10 (at most 0.1% under any transform) (Table S4).

Within the reintroduction areas the spread was much narrower. NNPK, EPL and DPKY were the most reproducible, with median CoV between 0.102 and 0.139 and mean CoV between 0.245 and 0.517, although the share of cells below 0.10 varied widely among them (0% to 49.1%). NPL, NEPL and NHNK were the least reproducible, with median CoV from 0.203 to 0.400, mean CoV from 0.333 to 0.694, a 90th percentile of 0.550 to 1.769, and no more than 6.7% of cells below 0.10 (Table S4). Across both scenarios, variability was concentrated at the margins of the predicted footprint rather than in the areas of high movement density (Figure S3; Table S4).

The three replicate‐mean landscape surfaces were nearly interchangeable, with pairwise correlations between the global means of 0.991 (p0.7 vs. p1.0), 0.982 (p1.0 vs. p1.5) and 0.959 (p0.7 vs. p1.5) for Spearman's *ρ*, and 0.997, 0.991 and 0.979 for Pearson's *r* (Table S5).

### 3.3 Priority Connectivity Kernels and Core Areas

The identity of the highest priority areas was unaffected by the resistance transform. Under the linear transform (p1.0; Figure 3, Table 4) the Connectivity Kernels were led by the WFCT–KKLT–MUL cluster (Mean 0.88), which took the maximum relative score for both Kernel Sum and Intensity (1.00) on a near‐maximal extent (0.96) and with a moderate protected‐area coverage (0.55; 32 protected areas). It was followed by EPL–VXP–XX (0.69), the most extensive kernel (Kernel Extent 1.00; 71,112 km^2^) and the best covered by protected areas after TNFC–RBGT–TH (0.60; 35 protected areas), but with intermediate Kernel Sum and Intensity (0.58 and 0.56). TNFC–RBGT–TH (0.67) had the highest protected‐area coverage of any kernel (1.00; 58 protected areas) and a high Intensity (0.68) on a smaller extent (0.58) and the lowest Kernel Sum of the three (0.41). The core areas were led by WFCT (0.98), which took the maximum for Kernel Sum, protected areas and Intensity simultaneously, followed by KKLT (0.84), which held the largest core patch (Kernel Extent 1.00; 14,046 km^2^) with lower protected‐area coverage (0.62). The remaining high‐ranking core areas were EPL (0.73) and CAR (0.65), both balanced across the four metrics (0.62–0.82 and 0.54–0.76), and TNFC (0.69), again ranking on protected‐area coverage (0.85) rather than on extent or Kernel Sum (0.65 and 0.52). The same complexes occupied the same positions under the concave (p0.7; Figure 4, Table 5) and convex (p1.5; Figure 5, Table 6) transforms, with composite scores changing by no more than a few hundredths: WFCT held a Core Area score of 0.98 under all three transforms, KKLT 0.84–0.86 and EPL 0.73–0.75.

**FIGURE 3:**
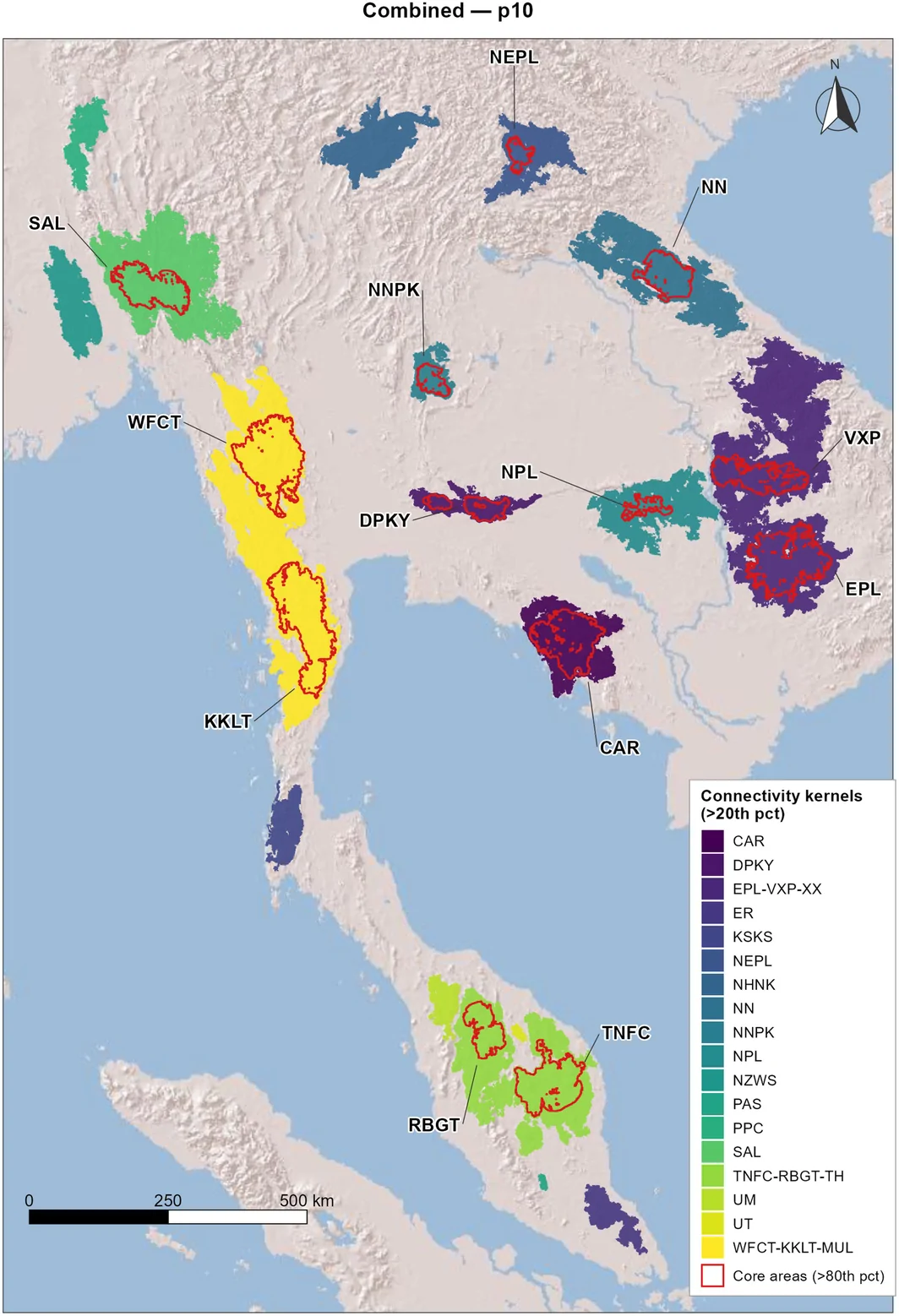
Priority connectivity kernels and core areas for the combined scenario under the linear resistance transform (p1.0). Connectivity Kernels (kernel values above the 20th percentile of their distribution) are filled and distinguished by colour according to the study‐area complex they originate from and intersect, as given in the legend; Core Areas (above the 80th percentile) are outlined in red and labelled with leader lines. The map is drawn on an Esri shaded‐relief basemap. The corresponding priority metrics and ranking are reported in Table 4; study‐area codes are as in Tables 1 and 2 (see also Figure 2).

**TABLE 4:** Priority ranking of the Connectivity Kernels (kernel values above the 20th percentile of their distribution; *p* = 0.2) and Core Areas (above the 80th percentile; *p* = 0.8), delineated on the combined landscape surface under the linear resistance transform (p1.0).

Site	Kernel Extent	Kernel Sum	PAs	Intensity	Kernel Extent rank	Kernel Sum rank	PAs rank	Intensity rank	Mean	*p*
WFCT–KKLT–MUL	68,039.34	1,925,693.22	32	28.3	0.96	1	0.55	1	0.88	0.2
EPL–VXP–XX	71,111.98	1,124,869.40	35	15.82	1	0.58	0.6	0.56	0.69	
TNFC–RBGT–TH	41,464.21	795,454.52	58	19.18	0.58	0.41	1	0.68	0.67	
CAR	19,622.04	514,234.06	12	26.21	0.28	0.27	0.21	0.93	0.42	
SAL	37,937.51	426,943.88	16	11.25	0.53	0.22	0.28	0.4	0.36	
NN	28,635.70	318,496.63	14	11.12	0.4	0.17	0.24	0.39	0.3	
DPKY	8076.39	141,262.13	6	17.49	0.11	0.07	0.1	0.62	0.23	
NNPK	5652.92	94,093.95	9	16.65	0.08	0.05	0.16	0.59	0.22	
NPL	19,322.07	147,780.96	14	7.65	0.27	0.08	0.24	0.27	0.22	
NHNK	14,684.84	138,446.71	4	9.43	0.21	0.07	0.07	0.33	0.17	
NEPL	12,878.64	106,224.49	2	8.25	0.18	0.06	0.03	0.29	0.14	
UM	4262.49	38,114.99	8	8.94	0.06	0.02	0.14	0.32	0.13	
KSKS	6733.36	47,856.44	9	7.11	0.09	0.02	0.16	0.25	0.13	
ER	5768.72	39,952.17	10	6.93	0.08	0.02	0.17	0.24	0.13	
NZWS	11,125.32	68,098.00	1	6.12	0.16	0.04	0.02	0.22	0.11	
PPC	6672.28	8930.42	3	1.34	0.09	0	0.05	0.05	0.05	
PAS	375.65	411.76	5	1.1	0.01	0	0.09	0.04	0.03	
UT	566.20	241.45	1	0.43	0.01	0	0.02	0.02	0.01	
WFCT	13,196.85	800,430.52	13	60.65	0.94	1	1	1	0.98	0.8
KKLT	14,045.70	725,422.90	8	51.65	1	0.91	0.62	0.85	0.84	
EPL	11,521.95	547,532.25	8	47.52	0.82	0.68	0.62	0.78	0.73	
TNFC	9109.42	413,619.28	11	45.41	0.65	0.52	0.85	0.75	0.69	
CAR	9344.65	431,759.52	8	46.2	0.67	0.54	0.62	0.76	0.65	
SAL	7590.43	289,460.72	4	38.13	0.54	0.36	0.31	0.63	0.46	
VXP	6962.22	227,648.72	5	32.7	0.5	0.28	0.38	0.54	0.43	
NN	5512.51	229,886.58	3	41.7	0.39	0.29	0.23	0.69	0.4	
RBGT	4399.25	138,944.63	5	31.58	0.31	0.17	0.38	0.52	0.35	
NNPK	2117.11	64,250.24	5	30.35	0.15	0.08	0.38	0.5	0.28	
DPKY	2335.02	72,633.71	4	31.11	0.17	0.09	0.31	0.51	0.27	
NPL	1825.35	54,653.38	3	29.94	0.13	0.07	0.23	0.49	0.23	
NEPL	1632.05	48,831.70	1	29.92	0.12	0.06	0.08	0.49	0.19	
DPKY	953.70	25,578.54	1	26.82	0.07	0.03	0.08	0.44	0.15	

*Note:* Kernel Extent = patch area (km^2^); Kernel Sum = sum of the kernel values within the patch; PAs = number of protected areas intersecting the patch; Intensity = Kernel Sum per km^2^ of patch area. Each metric is expressed relative to the highest value across all patches within the same percentile category, and the four relative ranks are averaged to give the composite Mean, on which patches are ordered. Patches are labelled with the study‐area complex or complexes they originate from and intersect; codes are as in Tables 1 and 2 (see also Figure 2).

**FIGURE 4:**
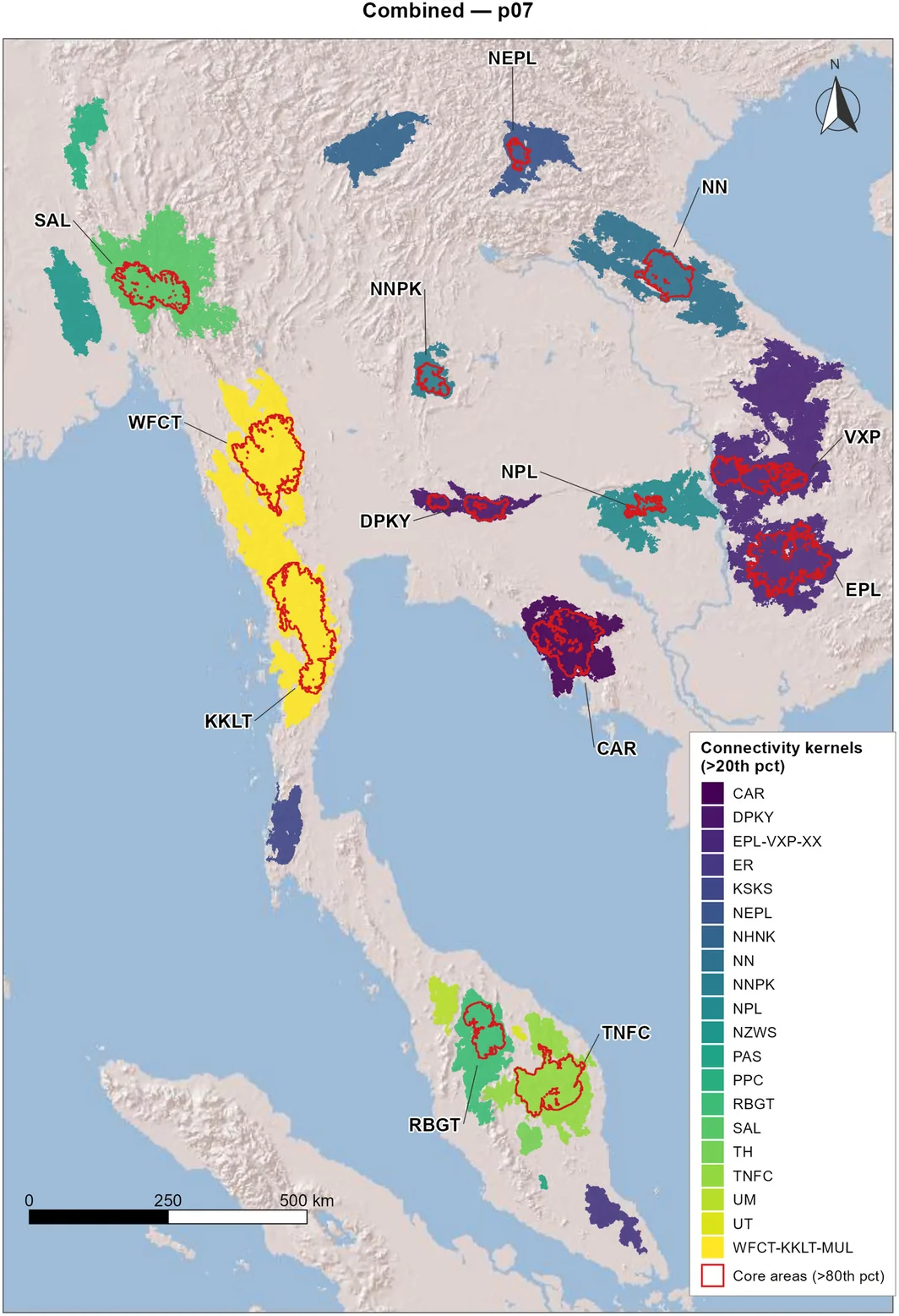
Priority connectivity kernels and core areas for the combined scenario under the concave resistance transform (p0.7). Connectivity Kernels (kernel values above the 20th percentile of their distribution) are filled and distinguished by colour according to the study‐area complex they originate from and intersect, as given in the legend; Core Areas (above the 80th percentile) are outlined in red and labelled with leader lines. The map is drawn on an Esri shaded‐relief basemap. The corresponding priority metrics and ranking are reported in Table 5; study‐area codes are as in Tables 1 and 2 (see also Figure 2).

**TABLE 5:** Priority ranking of the Connectivity Kernels (kernel values above the 20th percentile of their distribution; *p* = 0.2) and Core Areas (above the 80th percentile; *p* = 0.8), delineated on the combined landscape surface under the concave resistance transform (p0.7).

Site	Kernel Extent	Kernel Sum	PAs	Intensity	Kernel Extent rank	Kernel Sum rank	PAs rank	Intensity rank	Mean	*p*
WFCT–KKLT–MUL	64,267.39	1,782,487.38	32	27.74	0.96	1	0.94	1	0.98	0.2
EPL–VXP–XX	66,751.93	1,013,907.91	34	15.19	1	0.57	1	0.55	0.78	
TNFC	21,705.41	515,600.33	28	23.75	0.33	0.29	0.82	0.86	0.57	
CAR	18,807.84	440,704.77	12	23.43	0.28	0.25	0.35	0.84	0.43	
RBGT	15,071.42	228,613.50	24	15.17	0.23	0.13	0.71	0.55	0.4	
SAL	33,719.70	378,057.39	14	11.21	0.51	0.21	0.41	0.4	0.38	
NN	26,832.25	302,778.93	14	11.28	0.4	0.17	0.41	0.41	0.35	
NNPK	5084.90	85,918.52	9	16.9	0.08	0.05	0.26	0.61	0.25	
DPKY	7438.16	127,840.02	6	17.19	0.11	0.07	0.18	0.62	0.24	
NPL	17,498.52	121,832.57	13	6.96	0.26	0.07	0.38	0.25	0.24	
UM	3485.67	34,869.33	8	10	0.05	0.02	0.24	0.36	0.17	
ER	5230.78	37,618.09	10	7.19	0.08	0.02	0.29	0.26	0.16	
NHNK	11,563.88	114,363.91	2	9.89	0.17	0.06	0.06	0.36	0.16	
KSKS	6009.42	42,705.13	9	7.11	0.09	0.02	0.26	0.26	0.16	
NEPL	10,271.92	82,053.43	2	7.99	0.15	0.05	0.06	0.29	0.14	
NZWS	10,416.88	63,634.79	1	6.11	0.16	0.04	0.03	0.22	0.11	
TH	1916.52	13,209.69	5	6.89	0.03	0.01	0.15	0.25	0.11	
PPC	6238.28	8029.85	3	1.29	0.09	0	0.09	0.05	0.06	
PAS	299.97	332.74	5	1.11	0	0	0.15	0.04	0.05	
UT	435.82	140.40	1	0.32	0.01	0	0.03	0.01	0.01	
WFCT	12,351.64	748,905.65	11	60.63	0.93	1	1	1	0.98	0.8
KKLT	13,223.28	666,364.71	8	50.39	1	0.89	0.73	0.83	0.86	
EPL	10,687.69	491,076.09	8	45.95	0.81	0.66	0.73	0.76	0.74	
TNFC	8702.77	395,953.58	11	45.5	0.66	0.53	1	0.75	0.73	
CAR	8588.80	365,031.39	7	42.5	0.65	0.49	0.64	0.7	0.62	
SAL	6804.48	250,152.44	3	36.76	0.51	0.33	0.27	0.61	0.43	
VXP	6069.60	194,080.89	5	31.98	0.46	0.26	0.45	0.53	0.43	
NN	5321.94	221,173.06	3	41.56	0.4	0.3	0.27	0.69	0.41	
RBGT	4238.78	131,855.39	5	31.11	0.32	0.18	0.45	0.51	0.37	
NNPK	1970.32	58,918.82	5	29.9	0.15	0.08	0.45	0.49	0.29	
DPKY	2124.41	63,877.11	4	30.07	0.16	0.09	0.36	0.5	0.28	
NPL	1214.47	35,049.03	3	28.86	0.09	0.05	0.27	0.48	0.22	
NEPL	1209.00	34,387.47	1	28.44	0.09	0.05	0.09	0.47	0.17	
DPKY	705.70	18,296.69	1	25.93	0.05	0.02	0.09	0.43	0.15	

*Note:* Kernel Extent = patch area (km^2^); Kernel Sum = sum of the kernel values within the patch; PAs = number of protected areas intersecting the patch; Intensity = Kernel Sum per km^2^ of patch area. Each metric is expressed relative to the highest value across all patches within the same percentile category, and the four relative ranks are averaged to give the composite Mean, on which patches are ordered. Patches are labelled with the study‐area complex or complexes they originate from and intersect; codes are as in Tables 1 and 2 (see also Figure 2).

**FIGURE 5:**
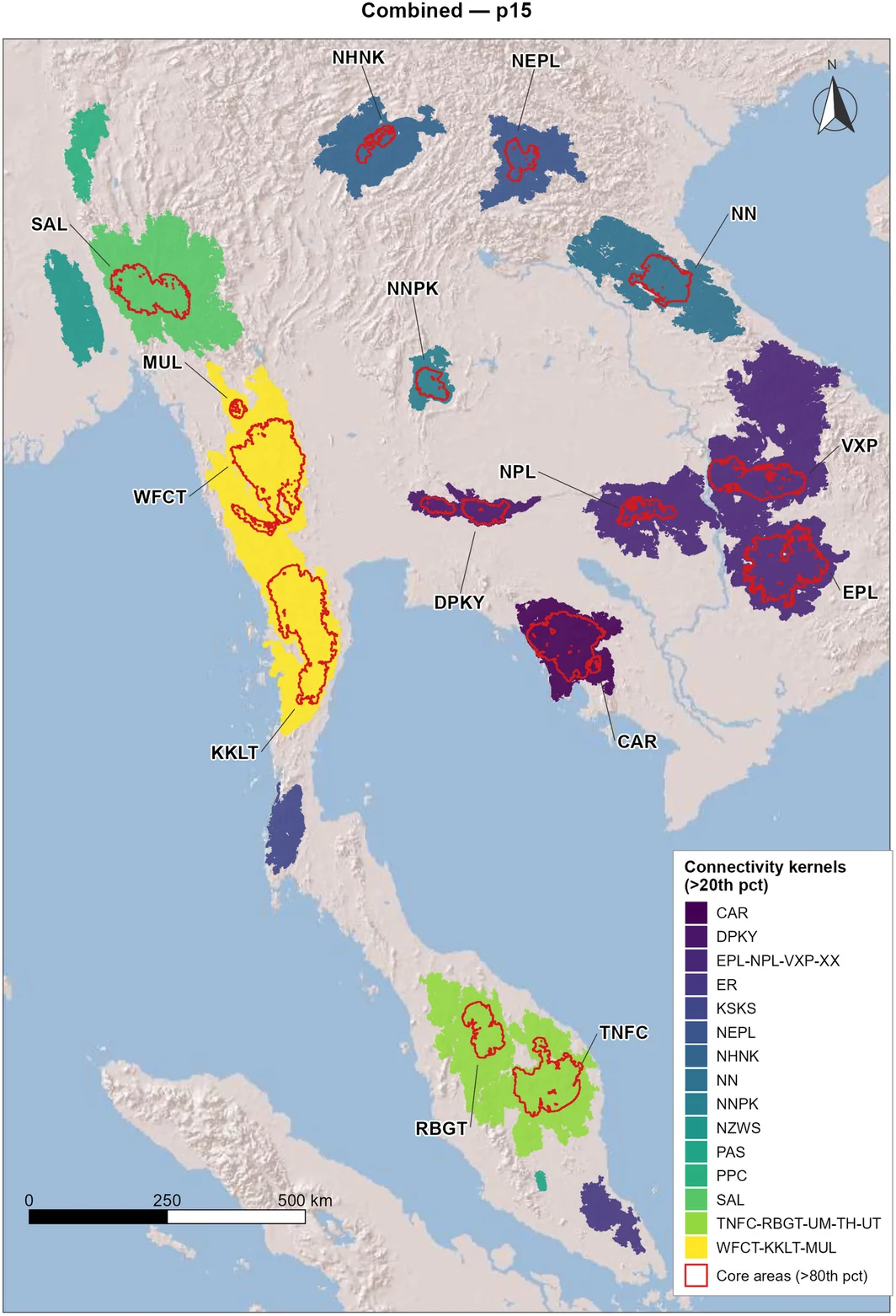
Priority connectivity kernels and core areas for the combined scenario under the convex resistance transform (p1.5). Connectivity Kernels (kernel values above the 20th percentile of their distribution) are filled and distinguished by colour according to the study‐area complex they originate from and intersect, as given in the legend; Core Areas (above the 80th percentile) are outlined in red and labelled with leader lines. The map is drawn on an Esri shaded‐relief basemap. The corresponding priority metrics and ranking are reported in Table 6; study‐area codes are as in Tables 1 and 2 (see also Figure 2).

**TABLE 6:** Priority ranking of the Connectivity Kernels (kernel values above the 20th percentile of their distribution; *p* = 0.2) and Core Areas (above the 80th percentile; *p* = 0.8), delineated on the combined landscape surface under the convex resistance transform (p1.5).

Site	Kernel Extent	Kernel Sum	PAs	Intensity	Kernel Extent rank	Kernel Sum rank	PAs rank	Intensity rank	Mean	*p*
WFCT–KKLT–MUL	75,036.21	2,198,721.94	32	29.3	0.74	1	0.43	0.97	0.79	0.2
EPL–NPL–VXP–XX	101,667.89	1,534,696.20	50	15.1	1	0.7	0.68	0.5	0.72	
TNFC–RBGT–UM–TH–UT	54,529.76	922,063.60	74	16.91	0.54	0.42	1	0.56	0.63	
CAR	21,565.02	651,319.19	13	30.2	0.21	0.3	0.18	1	0.42	
SAL	44,709.18	523,683.72	19	11.71	0.44	0.24	0.26	0.39	0.33	
NN	31,691.03	346,020.20	14	10.92	0.31	0.16	0.19	0.36	0.25	
DPKY	8832.25	166,448.88	6	18.85	0.09	0.08	0.08	0.62	0.22	
NNPK	6564.69	108,632.56	10	16.55	0.06	0.05	0.14	0.55	0.2	
NHNK	21,388.11	193,078.81	4	9.03	0.21	0.09	0.05	0.3	0.16	
NEPL	17,519.51	173,234.97	2	9.89	0.17	0.08	0.03	0.33	0.15	
KSKS	7789.19	56,819.30	9	7.29	0.08	0.03	0.12	0.24	0.12	
ER	7338.77	44,971.48	11	6.13	0.07	0.02	0.15	0.2	0.11	
NZWS	12,306.05	75,500.87	1	6.14	0.12	0.03	0.01	0.2	0.09	
PPC	7576.75	10,685.48	3	1.41	0.07	0	0.04	0.05	0.04	
PAS	599.94	611.85	5	1.02	0.01	0	0.07	0.03	0.03	
WFCT	14,176.08	878,276.75	13	61.95	0.92	1	1	1	0.98	0.8
KKLT	15,447.99	833,961.31	8	53.99	1	0.95	0.62	0.87	0.86	
EPL	12,596.00	642,671.72	8	51.02	0.82	0.73	0.62	0.82	0.75	
TNFC	9586.27	439,490.54	12	45.85	0.62	0.5	0.92	0.74	0.7	
CAR	10,740.56	557,265.25	8	51.88	0.7	0.63	0.62	0.84	0.7	
SAL	8609.77	352,635.01	4	40.96	0.56	0.4	0.31	0.66	0.48	
VXP	8283.36	283,541.97	5	34.23	0.54	0.32	0.38	0.55	0.45	
NN	5717.65	241,257.37	4	42.2	0.37	0.27	0.31	0.68	0.41	
RBGT	4500.45	149,297.97	5	33.17	0.29	0.17	0.38	0.54	0.35	
NNPK	2237.46	69,809.38	5	31.2	0.14	0.08	0.38	0.5	0.28	
DPKY	2606.73	86,338.83	4	33.12	0.17	0.1	0.31	0.53	0.28	
NPL	2795.46	91,289.37	3	32.66	0.18	0.1	0.23	0.53	0.26	
NEPL	2565.70	85,643.25	1	33.38	0.17	0.1	0.08	0.54	0.22	
WFCT	1258.23	33,435.03	3	26.57	0.08	0.04	0.23	0.43	0.19	
NHNK	1738.72	47,460.22	2	27.3	0.11	0.05	0.15	0.44	0.19	
DPKY	1254.59	36,155.91	1	28.82	0.08	0.04	0.08	0.47	0.17	
MUL	565.29	15,007.37	1	26.55	0.04	0.02	0.08	0.43	0.14	

*Note:* Kernel Extent = patch area (km^2^); Kernel Sum = sum of the kernel values within the patch; PAs = number of protected areas intersecting the patch; Intensity = Kernel Sum per km^2^ of patch area. Each metric is expressed relative to the highest value across all patches within the same percentile category, and the four relative ranks are averaged to give the composite Mean, on which patches are ordered. Patches are labelled with the study‐area complex or complexes they originate from and intersect; codes are as in Tables 1 and 2 (see also Figure 2).

What the transform changed was not the ranking but the geometry of the priority network, and it did so in opposite directions at the two thresholds. At the Connectivity Kernel threshold, increasing landscape permeability merged patches: the combined surface yielded 20 kernels under p0.7, 18 under p1.0 and 15 under p1.5, as separate complexes coalesced into progressively larger multi‐forest clusters (Figures 3, 4, 5, Tables 4, 5, 6). TNFC, RBGT and TH were three distinct kernels under p0.7, merged into a single patch under p1.0, and incorporated UM and UT under p1.5; NPL likewise joined the EPL–VXP–XX cluster under p1.5. At the Core Area threshold the opposite occurred: the number of patches rose from 14 under p0.7 and p1.0 to 17 under p1.5, and three patches appeared only under the most permeable transform (one each in MUL and NHNK and a second in WFCT). A complex could also be represented by more than one patch. DPKY held two disjunct core areas under each transform (Figures 3, 4, 5, Tables 4, 5, 6) and NHNK obtained two distinct core areas under p1.0 in the reintroduction scenario (Table S9, Figure S14).

Intensity, the movement density per unit area, was the metric that most often decided position within the ranking, because it separated patches that concentrate movement from those that accumulate large totals simply by being extensive. The clearest contrast among the Connectivity Kernels under p1.0 was between CAR and SAL: CAR covered about half the extent of SAL (19,622 against 37,938 km^2^) but ranked above it (Mean 0.42 against 0.36), its movement being more than twice as concentrated (relative Intensity 0.93 against 0.40). The same mechanism carried DPKY and NNPK into mid‐table positions, seventh and eighth, on relative extents of only 0.11 and 0.08, their intensities (0.62 and 0.59) being close to twice the median of the kernel set (0.32). NPL, of extent comparable to CAR, moved in the opposite direction and fell to ninth because its movement was thinly spread (0.27). The same trade‐off operated at the top of the ranking: EPL–VXP–XX was the most extensive kernel of all, yet its relative Intensity was little more than half that of WFCT–KKLT–MUL (0.56 against 1.00) (Figure 3, Table 4).

Below this band, position was set by the absence of any strong metric rather than by trade‐offs between them. UM, KSKS and ER (all 0.13) and NZWS (0.11) combined moderate extents with low movement density, NZWS additionally intersecting a single protected area (0.02). PPC (0.05) was the extreme case: it enclosed 6672 km^2^ of kernel but accumulated only 8930 kernel units, against 1,925,693 over 68,039 km^2^ in the leading cluster, so a sizeable patch on the map corresponded to very little predicted movement. PAS and UT closed the ranking (0.03 and 0.01), negligible on every metric (Table 4).

Among the Core Areas this contrast was much weaker. Thresholding at the 80th percentile retains only the densest cells, so Intensity varied within a narrow band (0.44–1.00 across the 14 patches) and could no longer discriminate strongly between patches; extent and protected‐area coverage therefore dominated the ordering, giving a middle band of SAL (0.46), VXP (0.43), NN (0.40) and RBGT (0.35) and a lower one of NNPK (0.28), DPKY (0.27), NPL (0.23) and NEPL (0.19). The rankings obtained for the extant range and the reintroduction areas separately reproduced these patterns under all three transforms, with the same complexes leading each scenario (Tables S6–S11; Figures S7–S18).

### 3.4 Robustness of Priority Rankings

Concordance among the three transforms was high in every scenario and at both thresholds (Table 7). For the Core Areas the ranking was effectively invariant: Kendall's *W* reached 1.000 in the extant range and in the combined scenario, and 0.985 in the reintroduction areas, with mean Spearman correlations of 1.000, 0.999 and 0.978 respectively (all *p* ≤ 0.017). For the Connectivity Kernels, concordance was slightly lower but still strong, with *W* being 0.884 in the extant range and in the combined scenario and 0.938 in the reintroduction areas (mean *ρ* 0.825–0.906; all *p* ≤ 0.002). The same pattern held when the complexes were ranked in isolation rather than on the landscape surfaces, with *W* between 0.945 and 0.985.

**TABLE 7:** Concordance of the priority ranking across the three resistance transforms (p0.7, p1.0, p1.5), by level, scenario and percentile threshold.

Level	Scenario	*P*	*n*	Kendall *W*	Friedman *χ* ^2^	df	*p*	Spearman *ρ*
Landscape	Extant	0.2	13	0.884	31.8	12	0.001	0.825
	0.8	5	1.000	12	4	0.017	1	
Reintroduction	0.2	11	0.938	28.1	10	0.002	0.906	
	0.8	9	0.985	23.6	8	0.003	0.978	
Combined	0.2	24	0.884	61	23	< 0.001	0.826	
	0.8	15	1.000	42	14	< 0.001	0.999	
Single area	Extant	0.2	13	0.985	35.5	12	< 0.001	0.978
	0.8	13	0.981	35.3	12	< 0.001	0.972	
Reintroduction	0.2	11	0.984	29.5	10	0.001	0.976	
	0.8	11	0.945	28.4	10	0.002	0.918	

*Note:* Level distinguishes the ranking obtained on the landscape surfaces, where the study‐area complexes are combined by cell‐wise maximum, from that obtained for each complex in isolation (single area). *P* is the percentile threshold, 0.2 for the Connectivity Kernels and 0.8 for the Core Areas. *n* is the number of study‐area complexes ranked, the fixed set of units on which the three rankings are compared; patches were expanded into their constituent complexes so that merged and separate patches remain comparable across transforms. Kendall *W* is the tie‐corrected coefficient of concordance among the three rankings, ranging from 0 (no agreement) to 1 (identical rankings), tested against a chi‐square distribution with the given Friedman *χ* ^2^, degrees of freedom (df) and significance (*p*). Spearman *ρ* is the rank correlation averaged over the three pairs of transforms. Values of *W* and *ρ* approaching 1 indicate that the three transforms order the complexes in the same way. Both are reported rounded to three decimal places, so a displayed value of 1.000 indicates agreement that is either complete or differs from unity by < 0.001.

The rank heat map (Figure 6) shows that every complex retained its rank under all three transforms at the Core Area threshold. At the Connectivity Kernel threshold, the leading complexes were equally stable, and all the movement occurred in the lower half of the ranking among the complexes that merge into multi‐forest clusters: TH, UM, UT and NPL shifted position as they were absorbed into the neighbouring clusters because a complex entering a cluster inherited the rank of the whole patch rather than its own.

**FIGURE 6:**
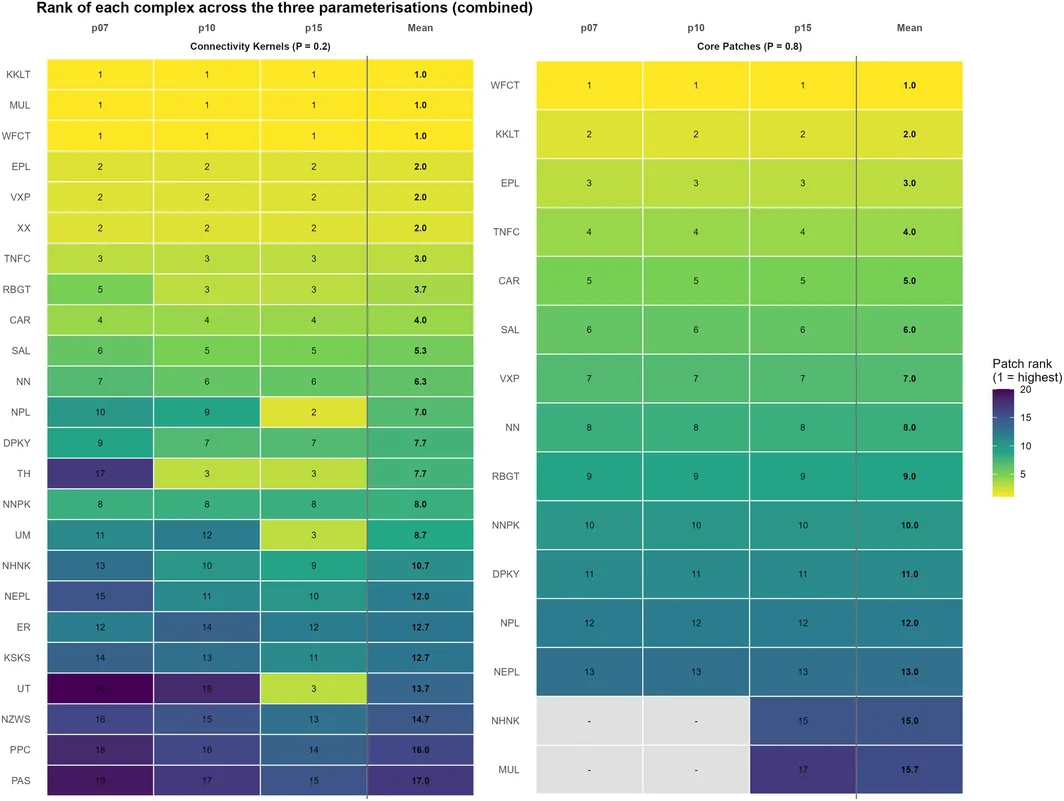
Rank held by each study‐area complex under the three resistance transforms (p0.7, p1.0, p1.5) in the combined scenario, for the Connectivity Kernels (*p* = 0.2, left panel) and the Core Areas (*p* = 0.8, right panel). Each cell gives the rank of the patch to which the complex belongs, 1 being the highest priority, and is shaded on a continuous scale from yellow (highest) to dark purple (lowest); the Mean column is the average rank across the three transforms, and rows are ordered by it. Because a patch may contain more than one complex, all the complexes sharing a merged patch inherit its rank and therefore appear with identical values: KKLT, MUL and WFCT all rank first at *p* = 0.2, as do EPL, VXP and XX in second place. Grey cells marked with a dash indicate complexes that formed no patch above the threshold under that transform, as for NHNK and MUL, which appear as core areas only under p1.5. In the Mean column such complexes are counted immediately below the last ranked patch of that transform, which is why MUL has a mean rank of 15.7: Two placeholder values of 15, one for each transform under which it forms no core area, and its actual rank of 17 under p1.5. Study‐area codes are as in Tables 1 and 2 (see also Figure 2).

The rank‐stability figure (Figure 7) quantifies this by scenario. Of the 24 units ranked at the Connectivity Kernel threshold in the combined scenario, nine held an identical rank under all three transforms, whereas UT (mean rank 13.67, range 3–20; SD 9.29), TH (7.67, range 3–17; SD 8.08), UM (8.67, range 3–12; SD 4.93) and NPL (7.00, range 2–10; SD 4.36) accounted for almost all of the variability of the combined scenario (Table S12). The extant and reintroduction scenarios behaved consistently, with UT and TH the most variable in the former and NPL in the latter, and with the reintroduction ranking the more stable of the two (6 of 11 units invariant). At the Core Area threshold variability nearly vanished: 14 of the 15 units in the combined scenario show an invariant rank and only MUL a variable one (mean rank 15.67, range 15–17; SD 1.15). Both MUL and NHNK, however, crossed the 80th percentile only under the most permeable transform (Figure 5; Table 6). A complex that forms no core area is counted immediately below the last ranked patch of that transform, so for these two the mean rank combines a placeholder position under p0.7 and p1.0 with their actual rank under p1.5, and for NHNK the two happen to coincide. Their entries should therefore be read as absence from the ranking under the two less permeable transforms rather than as a change of position. Per‐complex values for every scenario, threshold and level are given in Table S12.

**FIGURE 7:**
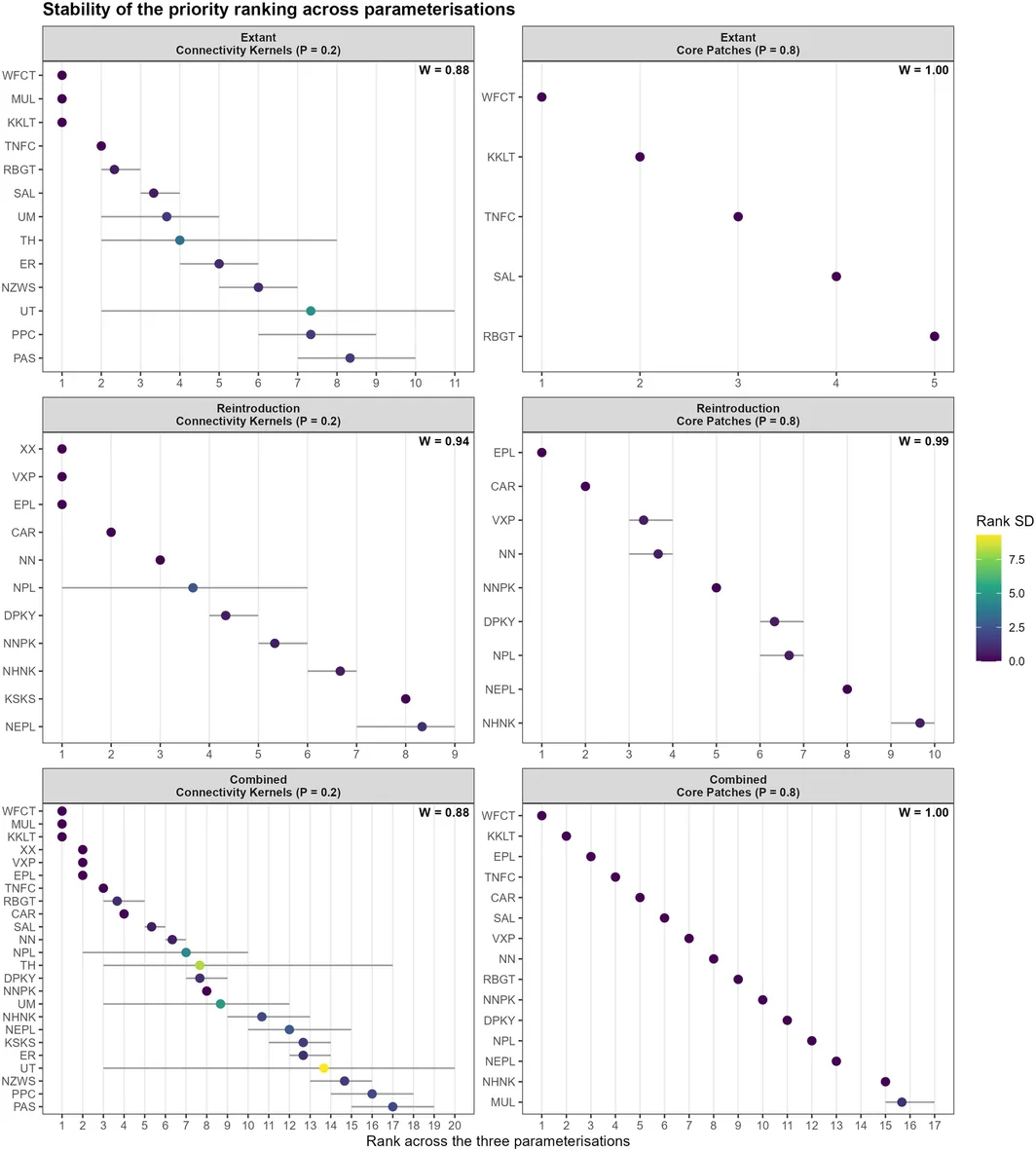
Stability of the priority ranking across the three resistance transforms (p0.7, p1.0, p1.5), by scenario and threshold: Extant range (top), reintroduction areas (middle) and combined (bottom), for the Connectivity Kernels (*p* = 0.2, left) and the Core Areas (*p* = 0.8, right). Each study‐area complex is plotted at its mean rank across the three transforms, with a horizontal line spanning the minimum to maximum rank it attained; complexes with no line held the same rank under all three transforms. Points are coloured by the standard deviation of the rank, from dark purple (invariant) to yellow (most variable). Kendall's coefficient of concordance (*W*) for the corresponding ranking is annotated in each panel, rounded to two decimal places. Complexes are ordered by mean rank and, as in Figure 6, those belonging to the same patch share its rank, so the wide ranges of TH, UT, UM and NPL reflect their absorption into multi‐forest clusters under the more permeable transforms rather than a change in their own connectivity. A complex that forms no patch above the threshold under a given transform is counted immediately below the last ranked patch of that transform, so mean ranks and ranges for complexes that qualify under only some of the transforms mix actual and placeholder positions (see Figure 6). Per‐complex values are given in Table S12; study‐area codes are as in Tables 1 and 2 (see also Figure 2).

The three multi‐forest clusters responded to the transform in the same direction but not to the same degree (Figure 8). Extent and Kernel Sum increased monotonically with landscape permeability in all three, by 17%–28% and 16%–35%, respectively, between p0.7 and p1.5, the largest gains being in EPL–NPL–VXP–XX (+21% and +35%), where NPL joins only at p1.5, and in RBGT–TH–TNFC–UM–UT (+28% and +16%), which gains two members at the same step. Protected‐area counts changed little (0% to +12%), and Intensity was the only metric to move in opposite directions between clusters: it rose modestly in EPL–NPL–VXP–XX (+12%) and KKLT–MUL–WFCT (+6%), where the merged patches were of comparable density, but fell in RBGT–TH–TNFC–UM–UT (−9%, from 18.6 to 16.9 kernel units per km^2^).

**FIGURE 8:**
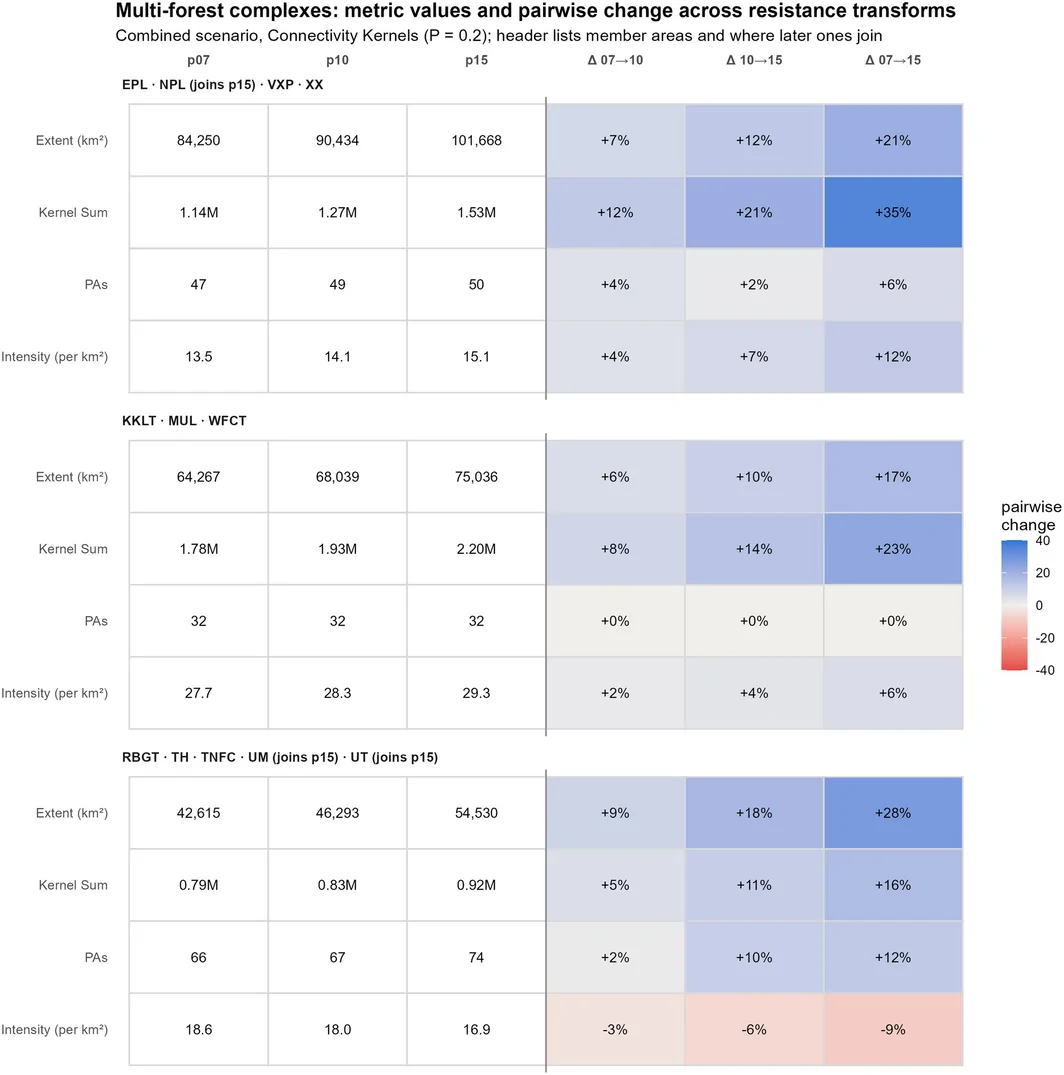
Metric values and pairwise change across the three resistance transforms for the multi‐forest clusters, that is the sets of forest complexes whose connectivity kernels coalesce into a single patch (combined scenario, Connectivity Kernels, *p* = 0.2). Each block follows one cluster, listing Kernel Extent (km^2^), Kernel Sum, the number of protected areas (PAs) and Intensity (kernel units per km^2^) under p0.7, p1.0 and p1.5 in the first three columns, and the pairwise per cent change between transforms (p0.7 → p1.0, p1.0 → p1.5, p0.7 → p1.5) in the last three, shaded from red (decrease) to blue (increase). A cluster is defined as the union of the complexes that share a patch under at least one transform, so that the same set of areas is compared throughout; where those complexes are still delineated as separate patches under a given transform, the value shown is the total over their individual patches rather than that of a single merged patch. The clusters do not all form at the same transform: KKLT–MUL–WFCT and EPL–VXP–XX are already merged at p0.7, with NPL joining the latter at p1.5, whereas RBGT–TH–TNFC merge only at p1.0, with UM and UT joining at p1.5; the block headers list the member areas and, in parentheses, the transform at which the later ones enter the cluster. Study‐area codes are as in Tables 1 and 2 (see also Figure 2).

### 3.5 Sensitivity of the Priority Metrics and Connectivity Differences

Averaged over all study‐area groups, the four metrics responded to the resistance transform in the same direction in every scenario and at both threshold levels (*p* = 0.2, the Connectivity Kernels; *p* = 0.8, the Core Areas), differing only in magnitude (Figure S19).

Between the two adjacent transforms p0.7 and p1.0, all metrics increased toward the more permeable surface. At the Connectivity Kernels threshold, the mean symmetric relative difference was +6 to +7 percentage points for Extent and Kernel Sum, +1 to +4 for protected‐area counts and +1 for Intensity, almost identically in the three scenarios. At the Core Areas threshold, the same comparison gave +4 to +10 for Extent and +5 to +12 for Kernel Sum, with the reintroduction areas already showing the largest values, +2 to +4 for protected areas and no more than +2 for Intensity.

Between the transforms p1.0 and p1.5 the changes were of comparable size but reversed in sign, as expected from the direction of the comparison. At the Connectivity Kernels threshold, extent fell by 9 points in all three scenarios and Kernel Sum by 9 to 11, while protected areas and Intensity moved by no > 2. At the core areas threshold, the reduction was somewhat larger in the reintroduction areas, −11 for Extent and −15 for Kernel Sum, against −7 and −8 in the extant range, with protected areas between −2 and −5 and Intensity between −1 and −4.

The comparison between the two most divergent transforms, p0.7 and p1.5, therefore produced the largest changes and separated the metrics most clearly. At the Connectivity Kernels threshold, Extent increased by 14 points in every scenario and Kernel Sum by 14 to 18, protected areas by 4 to 6, and Intensity by no > 3, falling to −1 in the extant range. At the Core Areas threshold, the contrast between scenarios was sharpest: in the reintroduction areas Extent rose by 20 and Kernel Sum by 25, the largest values recorded in the whole comparison, against +11 and +13 in the extant range and +13 and +17 in the combined scenario; protected areas increased by 4 to 8 and Intensity by 1 to 6.

The cell‐wise comparison of the continuous connectivity surfaces gives the same result spatially (Figure S20). Absolute differences remained small over the whole mapped footprint, staying below about 0.4 on the 0–1 scale and never approaching the upper end of the range, and they increased with the distance between transforms, being lowest for p0.7 versus p1.0 and highest for p0.7 versus p1.5. The larger differences were not confined to any particular part of the surface but were spread across the interiors of the most extensive connectivity areas, notably the Dawna–Tenasserim belt along the Thailand–Myanmar border, the Eastern Plains and Cardamom landscapes and the northern Lao and Vietnamese complexes, whereas the peninsular Malaysian areas changed almost imperceptibly under every comparison.

## 4 Discussion

The successful conservation of the Indochinese leopard will require differentiated, region‐specific strategies. This need is especially pressing given the recent regional extinctions of several leopard subpopulations (Rostro‐García et al. 2016, 2019, 2024), as well as parallel extinction trajectories observed for Indochinese tigers (Goodrich et al. 2022; Sanderson et al. 2023), and the complex suite of threats that continue to drive leopard declines (Rostro‐García et al. 2019, 2024).

This analysis provides the first range‐wide evaluation of structural connectivity for the Indochinese leopard, one of Asia's most threatened large carnivores. Using cumulative resistant kernel modelling (Compton et al. 2007), we identified opportunities to maintain or restore meta‐population processes. Despite severe range contractions and local extinctions, our results show that significant structural linkages persist within the Thai–Myanmar system and within Peninsular Malaysia, as well as across key forest complexes in Cambodia and Lao PDR (Tables 4, 5, 6; Figures 3, 4, 5). Mapping these Connectivity Kernels is crucial because structural connectivity forms the foundation for functional connectivity, but long‐term persistence depends on suitable habitat, adequate prey and the absence of anthropogenic threats such as poaching and persecution (Bleyhl et al. 2021; Day et al. 2020; Ghoddousi et al. 2020, 2021; Rostro‐García et al. 2019, 2024), all of which must be addressed within a broader socio‐ecological context.

Our framework complements demographic and ecological studies by offering a transparent spatial baseline that highlights where landscape conservation and/or recovery efforts may be most impactful. Comparable landscape‐level connectivity analyses have informed conservation planning for other wide‐ranging carnivores (Ash et al. 2026; Kaszta, Cushman, and Macdonald 2020; Rabinowitz and Zeller 2010; Pratzer et al. 2023; Calderón et al. 2024; Khosravi et al. 2018), demonstrating how structural maps can prioritise both core habitats and connectivity linkages in fragmented systems. Resistant kernel modelling captures gradients of movement probability across heterogeneous landscapes, avoiding arbitrary corridor delineation and instead identifying broad zones of opportunity (Unnithan Kumar and Cushman 2022; Jantz et al. 2025; Kaszta, Cushman, and Macdonald 2020; Kaszta, Cushman, Htun, et al. 2020; Cushman et al. 2018). This enables managers to target anti‐poaching, prey recovery, and habitat management where they can most effectively reinforce connectivity, tailored to local contexts and prevailing threats. In this way, our analysis using CoLa DSS (Jantz et al. 2025) provides not only an academic contribution but also a practical tool to guide where conservation actions will yield the greatest leverage for preventing extinction and restoring a lost apex predator.

### 4.1 Robustness of the Priority Network to Analytical Choices

A recurring concern in resistance‐based connectivity modelling is that the result may be an artefact of the resistance values assumed, particularly when those values are transferred from another species (Ash et al. 2020; Cushman et al. 2014; Zeller et al. 2018). We addressed this directly by repeating the whole analysis under three functional forms of the resistance surface, by re‐running it at a finer pixel grain, and by quantifying the variability introduced by the random placement of source points. Only the grain test required a second set of surfaces, comparing the ~1 km working grid with a ~500 m grid (Table 3; Table S3; Figures S1 and S2); as the predictions at both grains were highly concordant, the remainder of the suite was run on the ~1 km surfaces. Three comparisons at the level of the surfaces contrasted the transforms directly and were concordant: the correlation among predictions, computed per study area and replicate (Table 3; Table S3; Figures S1 and S2); the correlation among the replicate‐mean surfaces (Table S5) and the cell‐wise comparison of the raw connectivity surfaces (Figure S20). A fourth quantified the reproducibility of those surfaces across the 10 random source–point replicates (Table S4; Figure S3). The priority ranking that followed was correspondingly stable, almost invariant for the Core Areas and highly concordant for the Connectivity Kernels (Table 7; Figures 6 and 7; Table S12), and the change in the priority metrics between transforms was confined to the additive ones, Intensity being almost unaffected (Figure S19). Stability was greatest at the top of the ranking, where the priority areas lie, and the variation that remained was concentrated in the complexes that join or leave a merged patch (Figures 6 and 7; Table S12). The identification of WFCT, KKLT, EPL, TNFC and CAR as the highest‐priority core areas (Figures 6 and 7) is therefore a property of the landscape rather than of one parameterisation, which addresses a common criticism of connectivity models built on expert‐assigned or transferred resistance values (Zeller et al. 2012; Unnithan Kumar, Kaszta, and Cushman 2022; Unnithan Kumar, Turnbull, et al. 2022; Cushman et al. 2014).

### 4.2 Stronghold Systems in Extant Range

Our analysis supported two major strongholds for the Indochinese leopard, in agreement with previous research (Rostro‐García et al. 2016, 2019; Greenspan et al. 2021). The Thai–Myanmar complex, encompassing the WFCT and Kaeng Krachan–Lenya–Taninthayi (KKLT) areas, emerged as the most important stronghold, with WFCT achieving the highest mean ranking among Core Areas and the MUL–KKLT–WFCT cluster dominating Connectivity Kernels (Tables 4, 5, 6; Figures 3, 4, 5, 6, 7). This supports previous research confirming substantial structural connectivity across the Dawna–Tenasserim landscape, a region critical for multiple large carnivores (Sanderson et al. 2023; Ash et al. 2026; Duangchantrasiri et al. 2024; Greenspan et al. 2020, 2021, 2023; Rostro‐García et al. 2016, 2019). The structural integrity of the MUL–KKLT–WFCT system has been consistently demonstrated, from Indochinese leopards (Greenspan et al. 2021) to clouded leopards (Kaszta, Cushman, Htun, et al. 2020; Kaszta, Cushman, and Macdonald 2020) and Indochinese tigers (Ash et al. 2026; Suttidate et al. 2021), with recent long‐distance tiger dispersal further validating its regional importance (Simcharoen et al. 2022). This configuration did not depend on the resistance assumption: the three complexes formed a single connectivity kernel under all three transforms (Tables 4, 5, 6; Figures 3, 4, 5; Figure 8), and the cluster held the first rank under each of them (Table 7; Figures 6 and 7; Table S12).

Within WFCT, Huai Kha Khaeng Wildlife Sanctuary remains a key site for the subspecies (Rostro‐García et al. 2016, 2019). Although past surveys documented density declines (Simcharoen and Duangchantrasiri 2008) and current trends are unknown, populations might have improved given that increased law enforcement efforts there stabilised the local tiger population (Duangchantrasiri et al. 2016, 2024). Patterns of low density characterise Mae Wong and Khlong Lan National Parks (Phumanee et al. 2021), and Salak Phra (Rostro‐García et al. 2024), with leopard presence ascertained in Thung Yai Naresuan Wildlife Sanctuary (Vinitpornsawan et al. 2024). In the KKLT region, Kuiburi National Park maintained relatively recent stable populations (Pliosungnoen 2023), while density declines were reported from Kweekoh Wildlife Sanctuary in Myanmar's Karen State (Greenspan et al. 2023).

Our analysis identified additional areas of potential meta‐population connectivity despite current isolation. Although Salawin (SAL) achieved moderate connectivity metrics under all three resistance transforms (Tables 4, 5, 6; Figures 3, 4, 5), it likely lacks a viable leopard population (Rostro‐García et al. 2016). In contrast, NZWS and Panlaung Pyadalin Cave Wildlife Sanctuary (PPC) exhibited limited connectivity (Tables 4, 5, 6; Figures 3, 4, 5), consistent with the scarcity of leopard records in northern Myanmar since 2005 (Rostro‐García et al. 2016; Kyaw et al. 2024).

In peninsular Malaysia, the TH–TNFC–RBGT cluster represented the second most important stronghold of the extant range (Tables S6–S8) and the third on the combined surface (Tables 4, 5, 6; Figures 3, 4, 5), characterised by the largest number of protected areas of any Connectivity Kernel and encompassing a high‐ranking Core Area (TNFC) together with a smaller one (RBGT complex). Taman Negara historically supported robust populations (Kawanishi and Sunquist 2004; Lynam et al. 2007), with recent density estimates of 2.64 leopards/100 km^2^ (Rostro‐García et al. 2024). Kenyir Wildlife Corridor (within TNFC) showed comparable densities, ranging from 2.92 to 3.30 leopards/100 km^2^ (Hedges et al. 2015). Within the RBGT area, leopard presence in the late 1990_s_ was confirmed in Temenggor, Jengai and Ulu Temiang (Lynam et al. 2007), with records also documented in Hala Bala Wildlife Sanctuary (Thailand) (Kitamura et al. 2010; Kawanishi et al. 2010; Ngoprasert et al. 2012; Hambali et al. 2021), forming a larger trans‐boundary landscape (Rostro‐García et al. 2016). This cluster is also where the assumed permeability of the modified matrix changes the management units that follow from the analysis, and not only the magnitude of the metrics. Under the concave transform TNFC, RBGT and TH are three disjunct connectivity kernels (Table 5; Figure 4); under the linear transform they form a single one (Table 4; Figure 3); and under the convex transform they are joined by Ulu Muda and Ulu Temiang into one unit spanning 74 protected areas (Table 6; Figures 5 and 8). The larger extent is not matched by more intensive use, since this is the only cluster whose movement density falls as it grows, the areas absorbed being less intensively used than the original core (Figure 8). Whether these complexes are treated as one management unit or as several therefore depends on an assumption about matrix permeability for which no leopard‐specific empirical basis yet exists.

Several smaller areas with recorded leopard presence remained isolated from this main complex. These include the Endau Rompin complex (ER) (Rayan et al. 2013; Rayan 2007) and Pasoh Forest Reserve (PAS), which remained separate under all three resistance transforms (Tables 4, 5, 6; Figures 3, 4, 5), and Ulu Muda Forest Reserve (UM) (Tan et al. 2015) and Ulu Temiang Forest Reserve (UT), which were absorbed into the Malaysian cluster only under the most permeable transform (Table 6; Figures 5 and 8), indicating that their isolation is conditional on how strongly non‐forest habitat is penalised (in ecological terms, how leopards would exploit non‐forest habitat for dispersal and explorative forays). Recent corridor assessments detected leopard movement between Taman Negara and Sungai Yu, but no evidence around Tengku Hassanal Wildlife Reserve (TH) (Magintan et al. 2022), an important component of the multi‐forest Malaysian connectivity kernel (Tables 4 and 6; Figures 3, 5 and 8). However, occasional detections in previously unsurveyed areas like Ulu Sat Forest Reserve suggest some residual landscape permeability (Hizam et al. 2024). The vulnerability of isolated populations is exemplified by Ayer Hitam, where four leopards were recorded in 2008 (Sanei et al. 2011); this likely represents an unrecoverable relict population, if not already locally extinct.

### 4.3 Recovery Potential

Several promising areas for potential Indochinese leopard recovery were identified across its former eastern range. The VXP–EPL–XX complex emerged as the most structurally intact landscape based on our connectivity metrics, encompassing two of the leading core areas of the eastern range, the EPL and Virachey–Xe Pian (VXP), which held that position under every resistance assumption (Tables 4, 5, 6; Figures 3, 4, 5). The NPL and Nakai–Nam Theun National Park (NN) stood as complementary components of this recovery network, demonstrating potential for structural connectivity with the VXP–EPL–XX complex: under the most permeable transform NPL coalesced with that cluster into a single patch, whereas NN remained a separate kernel under all three transforms while holding a mid‐ranking Core Area (Tables 4, 5, 6; Figures 3, 5 and 8). The Cardamom Mountains (CAR) complex, with no leopard records since 2016 (Gray et al. 2017), although isolated, showed significant potential with its kernel and core area persisting under every resistance assumption (Tables 4, 5, 6; Figures 3, 4, 5). The movement it sustains is concentrated rather than thinly spread, forming a coherent unit for management while remaining isolated.

The EPL exemplifies the rapid collapse of leopard populations across the region. In 2009, Srepok Wildlife Sanctuary supported 3.6 individuals/100 km^2^ (Gray and Prum 2012), but densities fell to 1.0 leopard/100 km^2^ by 2014 (Rostro‐García et al. 2018) and became extirpated by 2021 (Rostro‐García et al. 2023). Phnom Prich Wildlife Sanctuary, which showed densities of 0.88–0.92 leopard/100 km^2^ in 2016, saw its leopard population reduced to a single individual by 2021 despite increased patrolling efforts (Rostro‐García et al. 2023, 2024).

Similar patterns of decline characterised other candidate reintroduction areas. Virachey National Park in Cambodia, part of the VXP–EPL–XX system, lost its leopards between 2001 and 2014 (Gray et al. 2012; Rostro‐García et al. 2016), while both Xe Xap National Protected Area (XX) and Xe Pian National Protected Area (within VXP), in southern Lao PDR, showed no recent records despite confirmed presence in the 1990_s_ (Timmins et al. 1993; Steinmetz et al. 1999; Rostro‐García et al. 2016). The NPL recorded leopards in Chhep Wildlife Sanctuary from 2012 to 2014 (Suzuki et al. 2017), with just two individuals in Preah Vihear between 2012 and 2015, but none detected in the final year (A. Suzuki, unpubl. data). Additional sightings were reported from Siem Pang Wildlife Sanctuary in 2002 and 2011, along with unconfirmed signs (BirdLife International 2012). Nakai–Nam Theun (NN)'s population collapsed from an estimated 30–40 individuals in the mid‐1990_s_ (Timmins and Evans 1996) to zero detections during intensive camera trapping from 2006 to 2011 (Coudrat et al. 2014).

In Thailand, the Nam Nao National Park—Phu Khiew Wildlife Sanctuary complex (NNPK) sustained a compact connectivity kernel but only a marginal core area (Tables 4, 5, 6; Figures 3, 4, 5). Its limited contribution reflects its setting more than its size: the complex is enclosed by an intensively cultivated and settled matrix that kept it separate from every other complex under all three resistance assumptions, so the intervening landscape, rather than the reserve itself, would require intensive management to restore connectivity northwards. Although leopards were historically present in NNPK (Borries and Koenig 2014), recent surveys have failed to detect them (Rostro‐García et al. 2016).

In northern Lao PDR, the Nam Ha National Protected Area—Nam Kan National Park complex (NHNK) and Nam Et–Phou Louey National Park (NEPL) contributed little to the predicted network (Tables 4, 5, 6; Figures 3, 4, 5), with NHNK reaching the core‐area threshold only under the most permeable assumption (Table 6; Figure 5). NHNK supported leopards until the late 1990_s_ (Duckworth et al. 1999) and may serve as the basis for a future northern meta‐population, though the status of leopards in southern China remains uncertain (Laguardia et al. 2017; Rostro‐García et al. 2016). NEPL, despite its status as Lao PDR's best protected area, last recorded leopards in 2004 (Johnson et al. 2006), with no detections in subsequent surveys (Rasphone et al. 2019).

In Thailand, the Khlong Saeng Wildlife Sanctuary–Khao Sok National Park Complex (KSKS) retained a Connectivity Kernel, but lacked a Core Area and showed no structural connectivity to the larger Thai–Myanmar complexes (MUL–KKLT–WFCT) under any resistance scenario (Tables 4, 5, 6; Figures 3, 4, 5). Leopards were last recorded in KSKS in 2014 (Rostro‐García et al. 2016) and were absent in recent surveys (Rostro‐García et al. 2024; Petersen et al. 2020). The Dong Phayayen–Khao Yai Forest Complex (DPKY) retained two separate core areas, a sign of internal fragmentation, and its kernel remained isolated under every resistance transform (Tables 4, 5, 6; Figures 3, 4, 5; cf. Ash et al. 2026), although the eastern network lies markedly closer to it under resistance p1.5 (Figure 5, Figures S6 and S18). Historical records documented leopard presence at Ta Phraya National Park (Ngoprasert et al. 2012), but recent camera‐trap surveys suggested leopards are now likely extirpated (Rostro‐García et al. 2016).

### 4.4 Implications for Prioritisation Under Scenario Uncertainty

The resistance transforms changed the magnitude of the metrics and the geometry of the network rather than the identity of the priority areas. This distinction matters for how the priority tables should be read (Tables 4, 5, 6; Figures 3, 4, 5; Figure S19). Kernel Extent and Kernel Sum are additive and increase systematically as the landscape is made more permeable, whereas Intensity, being a ratio, was almost unaffected, and protected‐area counts change only when a patch grows into a reserve it did not previously intersect. Absolute extents and Kernel Sums should therefore be read as conditional on the assumed resistance rather than as fixed properties of the landscape, whereas Intensity and the resulting ranking are comparable across assumptions. At the Core Area threshold, sensitivity was greatest in the candidate reintroduction areas, where connectivity is hypothetical and unconstrained by an occupied range, and least across the extant range; at the Connectivity Kernel threshold the two responded almost identically (Figure S19; Section S5, archived data).

The same permeability that increases the additive metrics also merges patches, and coalescence is not automatically a gain in connectivity quality (Tables 4, 5, 6; Figures 3, 4, 5 and 8). Extent and Kernel Sum necessarily rise when complexes merge, but Intensity indicates whether the ground gained carries comparable movement: it fell in the Malaysian cluster as Ulu Muda and Ulu Temiang were absorbed, because those areas are less intensively used than the original core, whereas it rose in the eastern cluster and in the Thai–Myanmar system (Figure 8). Where a merged cluster is adopted as a single management unit, its movement density should therefore be checked rather than inferred from its new size. The complexes whose rank moved most across transforms are those that enter or leave a cluster (Figures 6 and 7; Table S12): their position in the ranking depends on the resistance assumption, but their standing relative to the priority areas does not.

The thresholded priority patches are, moreover, a conservative rendering of the structure the model predicts. Before any percentile threshold is applied, the continuous connectivity surface of the combined scenario resolves into a small number of large connected blocks (Figures S4–S6). Under the linear and convex transforms the largest of these span the eastern Cambodian plains and the southern Annamites in the east, and the Dawna–Tenasserim range in the west; together with the peninsular Malaysian block they account for about three quarters of a reachable footprint of 454,000 and 524,000 km^2^ respectively (Figures S5 and S6; Table S4). Under the concave transform the same blocks are present but smaller and more fragmented, the three largest covering only about half of a smaller footprint (Figure S4; Table S4). These blocks are not internally uniform. Some of the linkages within them are strong enough to survive the percentile threshold: WFCT, KKLT and MUL, and EPL, VXP and XX, remain a single connectivity kernel under every transform (Tables 4, 5, 6; Figures 3, 4, 5; Figure 8). Others exist only among the lowest predicted values of the continuous surface and break as soon as any threshold is applied: SAL is attached to the western block and NN to the eastern one only once the matrix is treated as more permeable, and PPC only under the most permeable assumption (Figures S5 and S6). The first identifies ground that could carry movement as the landscape stands; the second, ground that could do so only if the intervening matrix were restored.

### 4.5 Institutional Management

Our analysis reveals complex and varying relationships between landscape structural integrity and protected area coverage across the Indochinese leopard's range. These patterns reflect broader challenges in protected area management across Southeast Asia, where institutional capacity, funding and enforcement effectiveness vary significantly between and within countries, shaping conservation outcomes (Rostro‐García et al. 2016, 2019).

The Thai–Myanmar system exemplifies this complexity. Much of the landscape within Myanmar's portion of the MUL–KKLT–WFCT Connectivity Kernel remains outside formal protection (Greenspan et al. 2020). The central Dawna Range, a critical corridor linking the Western Forest Complex with the Karen Hills (Ash et al. 2026), lacks designated conservation status despite its ecological importance (Greenspan et al. 2020). Even where protection exists, as in Kweekoh Wildlife Sanctuary, key connecting habitats remain vulnerable to encroachment and development (Greenspan et al. 2020, 2021). This shortfall reflects broader governance challenges in Myanmar. Only about 26.8% of protected areas in Karen State receive active management (Greenspan et al. 2020). Proposed areas like Lenya National Park face jurisdictional overlap and competing mandates (Greenspan et al. 2020). In regions such as Taninthayi, conservation governance is further complicated by contested land tenure and divergent stakeholder visions for land use (Paul 2018; Greenspan et al. 2020). Our own results quantify this shortfall. As the assumed permeability of the matrix increases, the MUL–KKLT–WFCT kernel gains almost 17% in extent and 23% in accumulated movement, yet the number of protected areas it intersects does not change at all, remaining at 32 under all three transforms (Tables 4, 5, 6; Figure 8). The additional connectivity predicted in this stronghold therefore brings no further reserve into the network, whereas the equivalent expansion in peninsular Malaysia draws in eight more, from 66 to 74 when its five constituent complexes are counted together, a 12% increase in the number of protected areas intersected (Figure 8).

The Malaysian system demonstrates comprehensive protected area coverage, with the TH–TNFC–RBGT complex spanning 58 protected areas under the linear transform, the largest number of any Connectivity Kernel (Table 4; Figure 3), and 74 once Ulu Muda and Ulu Temiang are absorbed under the most permeable transform (Table 6; Figures 5 and 8). The system likely benefits from Malaysia's higher socioeconomic status and strict penalties for wildlife crime, which may help reduce local involvement in poaching (Rostro‐García et al. 2024). However, some forest reserves in this key stronghold are less protected and susceptible to illegal activities, including cross‐border encroachment (Kawanishi and Sunquist 2004; Lam et al. 2023). Recent assessments highlighted emerging threats even in long‐established protected areas such as Taman Negara, particularly from illegal hunting and deforestation (Clements et al. 2021; Lam et al. 2023).

In potential reintroduction areas across Cambodia and Lao PDR, mismatches between ecological potential and protected areas coverage are more pronounced. The VXP–EPL–XX complex shows high structural connectivity and nominal protected area coverage (Tables 4, 5, 6; Figures 3, 4, 5), but limited institutional effectiveness, particularly in safeguarding core habitats. This gap is evident in the historical trajectory of population collapse across these landscapes (Rostro‐García et al. 2023). Despite international support since 2005, Nam Et–Phou Louey National Park (NEPL), one of Lao PDR's flagship protected areas, failed to sustain its leopard and tiger populations (Johnson et al. 2016; Rasphone et al. 2019).

In Cambodia, management effectiveness varies considerably across reserves. While Phnom Prich Wildlife Sanctuary and Srepok Wildlife Sanctuary have benefitted from government and NGO support, others such as Virachey National Park have seen minimal conservation investment (Gray et al. 2012). However, even enhanced protection has not ensured success, as leopards are now functionally extinct if not fully extirpated from both Phnom Prich and Srepok, despite expanded anti‐poaching initiatives (Rostro‐García et al. 2023). These outcomes suggest that current protected area models, especially in poaching hotspots, may require fundamental restructuring to address the intensity and scale of illegal hunting.

These regional contrasts highlight the critical importance of transboundary coordination (Farhadinia et al. 2021; Rostro‐García 2021), particularly in the Thai–Myanmar complex. Thailand's protected areas benefit from consistent national and international funding, with notable successes such as reduced poaching in Huai Kha Khaeng Wildlife Sanctuary (Duangchantrasiri et al. 2016; Simcharoen and Duangchantrasiri 2008). By comparison, Myanmar's protected areas receive less support, and their governance is often complicated by disputes between central and autonomous authorities (Greenspan et al. 2020, 2021). Political instability and ongoing civil unrest further limit conservation capacity and can pose indirect threats to biodiversity and habitats (Greenspan et al. 2020, 2021; Woods 2021). Furthermore, deforestation and poaching are expected to intensify as stability returns and development resumes (Connette et al. 2017), reinforcing the urgency of coordinated transboundary planning (Greenspan et al. 2021). Addressing this asymmetry calls for measures that go beyond spatial planning. Joint management of the protected areas that adjoin the border, coordinated patrol coverage and shared intelligence on snare removal and wildlife trafficking, harmonised legal protection and penalties on either side, and a standing bilateral working group including the authorities that administer Karen State in practice would each address the enforcement asymmetry that a mapped network cannot itself resolve.

This disparity in institutional resources and governance effectiveness critically undermines the ecological functionality of what should operate as unified conservation landscapes (Rostro‐García et al. 2016; Rostro‐García 2021).

### 4.6 Implementation Priorities and Challenges

Despite recent population losses, our analysis demonstrates the persistence of high connectivity and core area potential across the extant range and candidate reintroduction landscapes (Tables 4, 5, 6; Figures 3, 4, 5; Tables S6–S11). The fundamental habitat structure supporting connectivity remains largely intact, but functional persistence and recolonisation ultimately depend on whether mortality and poaching pressures can be reduced, prey base restored and broader landscape restoration implemented (Montes‐Rojas et al. 2024; Day et al. 2020; Bleyhl et al. 2021; Ghoddousi et al. 2020, 2022).

Among the reintroduction areas, structural continuity is strongest across eastern Cambodia and Lao PDR (Tables 4, 5, 6; Figures 3, 4, 5; Tables S9–S11). Large‐scale analogues in other ecosystems (Rabinowitz and Zeller 2010; Calderón et al. 2024; Khosravi et al. 2018; Montes‐Rojas et al. 2024) show how structurally continuous landscapes can sustain meta‐population processes. Expanding this network northward would require targeted management intervention linking Nakai–Nam Theun National Park (NN), Nam Et–Phou Louey National Park (NEPL), and Nam Ha–Nam Kan complex (NHNK), potentially re‐establishing continuity toward China, echoing evidence that restoration corridors and stepping‐stone patches enhance connectivity in fragmented systems (Montes‐Rojas et al. 2024; Kanagaraj et al. 2013; Suraci et al. 2020; Diniz et al. 2021; Stewart et al. 2019). The continuous connectivity surfaces support this reading more strongly than the thresholded patches do. Under the linear and convex transforms, the eastern complexes resolve into a single block reaching from the Eastern Plains and the Northern Plains through Virachey–Xe Pian and Xe Xap to Nakai–Nam Theun (Figures S5 and S6); under the concave transform, the same block is present except for Nakai–Nam Theun, which remains separate (Figure S4). This is the spatial basis on which an eastern stronghold could be rebuilt if mortality risk were brought under control.

By contrast, Nam Nao–Phu Khiew complex (NNPK) and Dong Phayayen–Khao Yai forest complex (DPKY) appear too isolated and embedded in human‐dominated matrices to represent viable reintroduction landscapes under current conditions (Ash et al. 2026), making it unlikely that these complexes could function as part of a broader meta‐population network. The same considerations apply to the Cardamom Mountains complex (CAR), which, although important in its own right, may reflect broader cases where viable carnivore populations are effectively isolated by intense human footprint (Iannella et al. 2024; Castilho et al. 2015; Kaszta, Cushman, and Macdonald 2020).

Within this broader configuration, Khao Sok–Khlong Saeng complex (KSKS) would be the obvious candidate for extending the western network, although no continuity between KSKS and the MUL–KKLT–WFCT cluster was predicted under any resistance transform (Tables 4, 5, 6; Figures 3, 4, 5). Establishing that link would depend on national‐scale landscape restoration and conservation interventions (Montes‐Rojas et al. 2024) and its re‐connection with Malaysian strongholds appears unlikely due to extensive landscape modification in southern Thailand.

In the same way, the intervening landscapes linking NZWS, Panlaung Pyadalin Cave Wildlife Sanctuary (PPC), and SAL complex to MUL–KKLT–WFCT (Thailand–Myanmar stronghold), and Endau Rompin complex (ER) to the TH–RBGT–TNFC cluster (Malaysian stronghold), are heavily modified by human land use and would require extensive restoration and active management to re‐establish connectivity with the major strongholds (McGregor et al. 2014). These separations did not depend on the resistance assumption: at the Connectivity Kernel threshold NZWS, PPC, SAL and ER remained disconnected from the major strongholds under all three transforms, including the most permeable (Tables 4, 5, 6; Figures 3, 4, 5).

While substantial habitat remains (Gray et al. 2023), restoration will require long‐term commitments and context‐specific adaptation. Reintroduction into unoccupied but suitable areas is possible (Sanderson et al. 2023), but only if the drivers of past extinctions are resolved (Rostro‐García et al. 2019, 2023, 2024). For example, indiscriminate and widespread snaring in eastern Cambodia, part of a larger snaring crisis negatively affecting biodiversity across eastern Indochina, was the primary reason for the recent extirpation of leopards from the landscape (Rostro‐García et al. 2023). This indiscriminate snaring also is causing major declines and extirpations of potential leopard prey in eastern Cambodia, despite large tracts of suitable habitat remaining (Rostro‐García et al. 2023). Therefore, any reintroduction programme for leopards in the area must first address the snaring crisis and reverse the prey declines, a large task that likely will involve public education campaigns to reduce bushmeat consumption and the illegal wildlife trade, and necessarily involving the cooperation of law enforcement personnel as well as local and national government officials. Sustained snare removal reduces but does not eliminate the pressure: 11 years of patrolling in two reserves in central Viet Nam cut snare occupancy by 37% but left almost a third of the area still snared, at about US$220,000 a year (Tilker et al. 2024).

In the Thai–Myanmar stronghold, deforestation (Tun et al. 2021; Connette et al. 2017; Greenspan et al. 2021), infrastructure expansion (Helsingen et al. 2015; Greenspan et al. 2021), poaching (Greenspan et al. 2020; Clements et al. 2021; Rostro‐García et al. 2024), and political instability (Greenspan et al. 2021, 2023; Woods 2021; McEvoy et al. 2022) all constrain enforcement and recovery. These threats are especially concerning in a landscape where many protected areas are too small to sustain viable carnivore populations (Greenspan et al. 2020, 2021).

Large‐scale projects such as the Dawei Special Economic Zone and the Belt and Road Initiative threaten to sever existing habitat networks, particularly through planned transportation corridors across the Dawna–Tenasserim landscape (Bassi et al. 2016; Ng et al. 2020; Kaszta, Cushman, Htun, et al. 2020; Helsingen et al. 2015; Hughes 2019).

In Malaysia, similar fragmentation pressures are evident, caused by roads, plantations, dams and urban expansion (Lynam et al. 2007; Clements et al. 2021). Although selectively logged forests can still support leopards under proper management (Rostro‐García et al. 2024), increasing development has made connectivity between protected areas more difficult (Magintan et al. 2022). While religious beliefs and distance from major wildlife trade markets may reduce local poaching incentives (Belecky and Gray 2020; McEvoy et al. 2022), threats persist from encroachment and foreign exploitation (Clements et al. 2021; Lam et al. 2023).

Reintroduction areas, particularly in eastern Cambodia and Lao PDR, face even greater challenges. Their proximity to Vietnam and China exposes them to heavy poaching driven by wildlife trade demand (Jiao et al. 2021). Eastern Southeast Asia is a global snaring hotspot, with densities exceeding 110 snares/km^2^ in some areas (Belecky and Gray 2020). In Srepok Wildlife Sanctuary, over 3700 snares were removed in a single year (Rostro‐García et al. 2024), likely representing just 11%–30% of the total deployed (O'Kelly et al. 2018).

Prey depletion is another critical barrier to recovery. In the EPL and Yok Don National Park (Vietnam), key prey species such as large ungulates have been severely reduced (Groenenberg et al. 2020, 2023). In many areas, prey declines drive carnivore loss, often as a result of commercial poaching and subsistence hunting (Rostro‐García et al. 2019, 2023; Shairp et al. 2016; Sandalj et al. 2016). These conditions underscore the importance of re‐establishing viable prey bases before attempting leopard reintroductions (IUCN/SSC 2013).

To address these challenges, conservation efforts must be phased, localised and multi‐scale. Core priorities include expanding and effectively managing protected area coverage (Greenspan et al. 2021; Gray et al. 2023), strengthening law enforcement and improving cross‐border cooperation, especially in landscapes where inconsistent protection across national borders undermines regional connectivity (Farhadinia et al. 2021; Rostro‐García et al. 2016, 2024).

### 4.7 Methodological Limitations

While our analysis provides a novel and spatially comprehensive assessment of potential connectivity for the Indochinese leopard, several limitations should be acknowledged. First, our modelling framework is static and does not incorporate temporal dynamics such as population movement, colonisation or extinction processes. This limits our ability to capture how connectivity may evolve under changing ecological or management conditions (Barros et al. 2019; Ash et al. 2020, 2022; Fletcher et al. 2019; Zeller et al. 2024). The use of dynamic, individual‐based simulations, such as agent‐based models (Schumaker and Brookes 2018; Unnithan Kumar, Kaszta, and Cushman 2022; Unnithan Kumar, Turnbull, et al. 2022) or demographic and genetic simulators like CDPOP (Landguth and Cushman 2010) could improve realism by simulating demographic processes and dispersal over multiple generations.

Second, our resistance surface is based on land cover and does not explicitly incorporate ecological mortality risks such as poaching pressure or prey availability (Ghoddousi et al. 2020, 2021; Titus and Jachowski 2025; Ash et al. 2022; Vasudev et al. 2023). These factors, particularly snaring, are among the leading causes of leopard decline across Southeast Asia and play a critical role in determining population viability and functional connectivity (Rostro‐García et al. 2024; O'Kelly et al. 2018; Ghoddousi et al. 2020, 2021).

Third, our land cover data may not accurately represent future habitat conditions. Landscape change in the region is rapid, with ongoing infrastructure expansion and land‐use conversion potentially fragmenting even currently intact habitats (Ng et al. 2020; Hughes 2017). Incorporating projected development scenarios into dynamic connectivity models would help identify areas at risk of future isolation (Ash et al. 2020). The same caution applies to present conditions. The land‐cover map underlying the resistance surface describes the mid 2010s, and forest loss and fragmentation have continued in the region since; no more recent product offers an equivalent, thematically consistent classification across the whole range at a comparable resolution. Our estimates of structural connectivity should therefore be read as an upper bound, since forest converted since the mapping date can only weaken, not strengthen, the linkages identified here.

Climate change was likewise not represented in our resistance surface, and its likely consequences for the priorities identified here deserve to be stated qualitatively. Projected climatic change in mainland Southeast Asia is expected to reduce the extent of suitable habitat and to shift its distribution, with species tending to retreat toward higher elevations (Namkhan et al. 2022), so the position of suitable habitat within the large blocks identified here may move even where the blocks themselves persist. Because the leopard is a habitat generalist with a broad climatic tolerance (Jacobson et al. 2016; Stein et al. 2020), the direct climatic effect on the species is likely to be smaller than the indirect effects transmitted through vegetation change, prey distributions and, in particular, the expansion of agriculture into land that becomes newly cultivable (Feng et al. 2021; Hughes 2017); the last of these would act on precisely the intermediate, human‐modified classes whose resistance our transforms bracket. Where climate change has been modelled for other leopard subspecies, responses were strongly subspecies‐specific, some losing and others gaining suitable range, and in all of them the share falling inside protected areas was low and projected to decline further (Mitchell et al. 2024). The direction of change for the Indochinese leopard therefore cannot be inferred from the species as a whole. Elevational and latitudinal linkages, which our analysis treats as equivalent to any other, may therefore acquire disproportionate value over the coming decades, as recognised in connectivity planning designed explicitly around elevational gradients elsewhere in the tropics (Townsend and Masters 2015; McCullough et al. 2024).

Additionally, the thematic content of our input layers may overestimate connectivity in some regions. Re‐running the analysis on a ~500 m grid changed the predicted pattern very little (Table 3; Table S3; Figures S1 and S2), so the modelling grain is unlikely to be the limiting factor; what remains unrepresented is the finer texture of the landscape, since only primary and secondary roads were included, built‐up land was treated as a single class, and edge effects were not modelled. For example, areas within the MUL–KKLT–WFCT kernel, predicted as highly connected, have shown previously reduced functional connectivity (Greenspan et al. 2021). High‐resolution data on roads, settlements and edge effects would improve accuracy and better reflect barriers to leopard dispersal.

Empirical validation is another key gap (Creech et al. 2024). Our connectivity predictions would benefit from validation with telemetry or genetic data (Lehnen et al. 2021; Calderón et al. 2024; Zeller et al. 2018; Cushman et al. 2014), which could refine resistance values and help distinguish between habitat preferences of resident versus dispersing individuals (e.g., Elliot et al. 2014; Zeller et al. 2018). These datasets are also critical for identifying functional thresholds of connectivity relevant to conservation interventions. Such data would also resolve what the resistance transforms can only bracket. The transforms act on precisely the quantity a behavioural study would inform, namely how strongly a modified but forested matrix should be penalised relative to closed forest, and the resulting spread in the mapped network is a measure of that uncertainty rather than of statistical noise. There is at present no empirical basis for preferring any point along this axis for the Indochinese leopard, and habitat selection by resident animals is a poor guide to the resistance experienced by dispersers (Zeller et al. 2018).

Our understanding of current leopard populations is also incomplete. Reliable population estimates exist only for a few regions (e.g., Rostro‐García et al. 2024; Greenspan et al. 2023; Clements et al. 2021), with many others lacking recent assessments. Survey gaps are especially pronounced in Myanmar, where political instability limits monitoring (Greenspan et al. 2021). Outdated presence data may have affected our selection of source areas, which the analysis cannot test. Where the points fall within a selected area was randomised and tested directly: across the 10 replicate sets the per‐cell CoV of the landscape surface was low, with variability concentrated at the margins of the predicted footprint rather than in the areas of high movement density (Table S4; Figure S3). The identity of the source complexes and the total number of points, which follows from a single range‐wide density estimate, were not varied. The number of points is nonetheless unlikely to alter the ranking reported here. Points are allocated in proportion to the area of each complex, apart from rounding in the smallest ones, so applying the lower or the upper confidence limit of the density estimate changes how many points are placed in total but not how they are distributed among complexes. Thus, the relative allocation, which stands in for the carrying capacity of each complex, governs the relative connectivity metrics on which the ranking is built. Displacing an area from its position would instead require the ratios between complexes to change, that is, independent abundance estimates showing that a given complex holds far more or far fewer animals than its area implies. Such estimates are not available for most of the range. Where this has been tested directly with the same framework, simulating connectivity under low, medium and high population sizes for each landscape, the resulting priorities were largely insensitive to the starting population size (Ash et al. 2026). What population density does influence is the magnitude of the connectivity metrics rather than the ordering of the areas, and in dynamic simulations its effect was smaller than that of mortality risk and dispersal ability (Ash et al. 2020).

Finally, while our Connectivity Kernel and Core Area thresholds (20th and 80th percentiles, respectively) follow accepted practices (Cushman et al. 2013; Kaszta, Cushman, and Macdonald 2020), the percentile values themselves were not varied. Both thresholds were nonetheless carried through every analysis, and their consequences are partly characterised: within each threshold, the priority ordering was concordant across the resistance transforms (Table 7; Figures 6 and 7), while the geometry of the network responded in opposite directions, patches merging at the 20th percentile and additional core areas emerging at the 80th as landscape permeability increased (Tables 4, 5, 6; Figures 3, 4, 5; Figure 8). Threshold selection therefore affects the mapped extent and configuration of the priority network more than the identity of the priority areas, especially in the absence of species‐specific dispersal validation (Zeller et al. 2018).

## 5 Conclusions

This study, using the CoLa DSS system (Jantz et al. 2025), provides the first comprehensive assessment of connectivity for the Indochinese leopard across Southeast Asia. Our analysis confirms the Thai–Myanmar and the Peninsular Malaysian systems as critical strongholds (Rostro‐García et al. 2016, 2019), with the MUL–KKLT–WFCT and TH–TNFC–RBGT clusters supporting substantial structural habitat networks and Core Areas (Tables 4, 5, 6; Figures 3, 4, 5). However, these areas face mounting pressures from infrastructure development, deforestation and poaching (Helsingen et al. 2015; Greenspan et al. 2020, 2021, 2023; Rostro‐García et al. 2024; Clements et al. 2021; Lam et al. 2023), highlighting the urgent need for enhanced protection and transboundary cooperation (Farhadinia et al. 2021; Rostro‐García 2021).

In the eastern portion of the range, our findings reveal the VXP–EPL–XX cluster as the most promising candidate for population recovery, supported by strong structural connectivity and potential for creating a larger meta‐population with adjacent landscapes like Nakai–Nam Theun National Park (NN) and the NPL (Tables 4, 5, 6; Figures 3, 4, 5 and 8; Figures S5 and S6). However, the recent leopard extirpation despite extensive landscape structural integrity underscores that connectivity alone is insufficient for population persistence (Rostro‐García et al. 2023). The widespread snaring crisis and collapse of prey communities continue to be the most urgent conservation concerns in these areas (Rostro‐García et al. 2024; O'Kelly et al. 2018; Gray et al. 2018; Jiao et al. 2021; Belecky and Gray 2020). Restoring leopard connectivity in this landscape is conditional on first reducing mortality risk: unless snaring is brought under control and prey populations are allowed to recover, structural continuity will not translate into occupancy, and reintroduction into a connected but unsafe landscape would repeat the losses already documented in the region.

Conservation success will require strengthening protection in existing strongholds while simultaneously preparing potential reintroduction sites through intensive anti‐poaching efforts and prey recovery programmes. Transboundary coordination will be essential, particularly in landscapes where political boundaries and different governance intersect areas of ecological importance (Greenspan et al. 2020, 2021; Rostro‐García et al. 2024; Farhadinia et al. 2021).

The Indochinese leopard's decline reflects broader conservation challenges for large carnivores in Southeast Asia. However, the presence of substantial intact habitat networks with potential to maintain connectivity, combined with clear priorities for protection and restoration, offers cautious optimism for species recovery.

## Author Contributions

**Luciano Atzeni:** conceptualization (lead), data curation (lead), formal analysis (lead), investigation (lead), methodology (lead), supervision (equal), validation (equal), visualization (lead), writing – original draft (lead), writing – review and editing (lead). **Jan Kamler:** conceptualization (equal), data curation (supporting), methodology (supporting), validation (equal), writing – review and editing (equal). **Eric Ash:** data curation (equal), methodology (equal), visualization (equal), writing – review and editing (equal). **Pyae Phyoe Kyaw:** data curation (supporting), methodology (supporting), validation (equal), writing – review and editing (equal). **Akchousanh Rasphone:** data curation (supporting), methodology (supporting), validation (equal), writing – review and editing (equal). **Chanratana Pin:** data curation (supporting), methodology (supporting), validation (equal), writing – review and editing (equal). **Cedric Kai Wei Tan:** data curation (supporting), methodology (supporting), validation (equal), writing – review and editing (equal). **Susana Rostro‐García:** data curation (supporting), methodology (supporting), validation (equal), writing – review and editing (equal). **Patrick Jantz:** conceptualization (equal), funding acquisition (equal), methodology (equal), software (lead), supervision (equal), validation (equal), writing – review and editing (equal). **Ivan Gonzalez:** conceptualization (equal), methodology (equal), software (lead), validation (equal), writing – review and editing (equal). **Dawn Burnham:** funding acquisition (equal), project administration (equal), software (supporting), validation (equal), writing – review and editing (equal). **Samuel A. Cushman:** conceptualization (equal), funding acquisition (lead), methodology (equal), software (equal), supervision (equal), validation (equal), writing – review and editing (equal). **David W. Macdonald:** conceptualization (equal), funding acquisition (equal), methodology (equal), project administration (equal), software (equal), supervision (equal), validation (equal), writing – review and editing (equal).

## Funding

Development of the CoLa Decision Support System (DSS) was initially supported through a series of three grants from the Robertson Foundation to DWM (2012–2024) focused on Felid Landscapes. Subsequent support was provided by the NASA Biodiversity and Ecological Conservation Applied Sciences Program (grant 80NSSC21K1942), which enabled the integration and enhancement of CDPOP and UNICOR within CoLa and the advancement of the DSS framework. Additional funding for the fieldwork underpinning this research was provided by the Recanati–Kaplan Foundation and, more recently, by Panthera.

## Conflicts of Interest

The authors declare no conflicts of interest.

## Supporting information


**Table S1:** Reclassification of the Miettinen et al. land‐cover map of Southeast Asia into the base resistance surface. Each land‐cover category (Value) is assigned a base resistance (Resistance, 1 = most permeable to 100 = most resistant): closed‐forest classes = 1, regrowth/plantation/deciduous shrub/floodzone shrub = 20, mosaic/open/bare limestone = 50, water = 80, urban = 100. Three features not present in the Miettinen land‐cover map are burned into this categorical input raster separately: secondary roads at resistance 30, and primary roads and built‐up areas at resistance 100 (Table S2).
**Table S2:** Resistance values under the three functional‐form transforms. The base surface is raised to a power *p* and rescaled to 1–100 (*R*′ = 1 + (*R* ^*p*^ − 1)/(100^*p*^ − 1) × 99), keeping the extremes fixed (minimum → 1, maximum → 100) and bending the intermediate classes: **p07** (concave, *p* = 0.7) makes intermediate classes more resistant, **p10** (linear, *p* = 1.0) is the identity, and **p15** (convex, *p* = 1.5) makes them more permeable. Each column gives the resulting integer resistance for a land‐cover/feature class under that transform (the p10 column is the base value; Table S1).
**Table S3:** Overall agreement of the connectivity surfaces across the analysis choices, pooled over all study areas and replicates, and for the pixel rows also over the three resistance transforms (summarising the per‐area values in Figures S1 and S2). Rows are the comparison axes: *pixel* = 1 km vs. 500 m grain (at both grains), *resistance* = pairs of resistance transforms. For each of three coefficients, Pearson, Spearman (rank) and Pearson on log1*p*‐transformed values (down‐weighting the heavy tail of movement density), the median, minimum and maximum across all area‐replicate comparisons are reported.
**Table S4:** Reproducibility of the connectivity prediction across the 10 random source‐point replicates, per study area and for the whole landscape, for the three resistance transforms. Rows are grouped by transform (p07, p10, p15) and the groups are separated by a horizontal rule. Columns: transform = resistance transform; scope = study‐area code or GLOBAL (all areas combined per replicate); scenario = scenario the study area belongs to (extant or reintroduction; combined for GLOBAL); cov_median, cov_mean = median and mean per‐cell CoV over the reachable footprint; cov_p90 = 90th percentile of the per‐cell CoV (the high‐variability tail); pct_cells_cov_lt0.10 = percentage of footprint cells with CoV below 0.10 (highly reproducible); n_footprint_cells = number of cells with positive mean movement density (footprint size). Lower CoV and a larger share of cells below 0.10 mean a more reproducible prediction.
**Table S5:** Overall similarity of the three resistance transforms at the landscape scale: cell‐wise correlation between the global mean connectivity surfaces (replicate mean of all areas combined). Pearson below the diagonal, Spearman rank correlation above; the diagonal is 1 by definition. Values near 1 mean the three transforms yield almost the same landscape‐scale pattern.
**Table S6:** Priority ranking, Extant, p10 resistance transform (see the column definitions). Corresponding map: Figure S8.
**Table S7:** Priority ranking, Extant, p07 resistance transform (see the column definitions). Corresponding map: Figure S7.
**Table S8:** Priority ranking, Extant, p15 resistance transform (see the column definitions). Corresponding map: Figure S9.
**Table S9:** Priority ranking, Reintroduction, p10 resistance transform (see the column definitions). Corresponding map: Figure S14.
**Table S10:** Priority ranking, Reintroduction, p07 resistance transform (see the column definitions). Corresponding map: Figure S13.
**Table S11:** Priority ranking, Reintroduction, p15 resistance transform (see the column definitions). Corresponding map: Figure S15.
**Table S12:** Rank stability of each study‐area complex across the three resistance transforms, by scenario, level (Landscape = complexes ranked on the combined‐area surface; Single area = each complex ranked in isolation) and threshold *p* (0.2 = Connectivity Kernels, 0.8 = Core Areas). Mean rank = the complex's mean priority rank across the three transforms; SD = its standard deviation; Min/Max = its best and worst rank across the three transforms. At the Landscape level a complex takes, under each transform, the best (lowest) rank of any patch it belongs to, so its Min–Max range spans from the rank it holds when it forms a standalone patch to the higher rank it inherits when it coalesces into a multi‐forest cluster; this is why peripheral areas such as NPL, UM, UT and TH show a wide range at *p* = 0.2, where under the more permeable transforms they merge into a high‐priority cluster. A complex that forms no patch above the threshold under a given transform is counted immediately below the last ranked patch of that transform, so mean ranks and ranges for complexes that qualify under only some of the transforms mix actual and placeholder positions: at *p* = 0.8 in the combined scenario this applies to MUL and NHNK, which cross the 80th percentile only under p1.5. A small SD and a narrow Min–Max range indicate a ranking that is stable to the resistance transform.
**Figure S1:** Robustness of the cumulative resistant‐kernel connectivity surfaces to the analysis choices, for the extant forest complexes. For each study area the bars give the replicate‐mean Spearman rank correlation (±SD across the 10 source‐point replicates for the transform comparisons, and across the 30 replicate × transform combinations for the grain comparisons) between the connectivity surface computed under different choices: the resistance transforms (p0.7 vs. p1.0, p1.0 vs. p1.5, p0.7 vs. p1.5) and the grain (1 km vs. 500 m, reported at both grains). Values near 1 mean the spatial pattern is largely unaffected by the choice; lower values flag more sensitive areas.
**Figure S2:** Robustness of the cumulative resistant‐kernel connectivity surfaces to the analysis choices, for the reintroduction forest complexes. For each study area the bars give the replicate‐mean Spearman rank correlation (±SD across the 10 source‐point replicates for the transform comparisons, and across the 30 replicate × transform combinations for the grain comparisons) between the connectivity surface computed under different choices: the resistance transforms (p0.7 vs. p1.0, p1.0 vs. p1.5, p0.7 vs. p1.5) and the grain (1 km vs. 500 m, reported at both grains). Values near 1 mean the spatial pattern is largely unaffected by the choice; lower values flag more sensitive areas.
**Figure S3:** Spatial pattern of the per‐cell replicate CoV for the combined scenario under the three resistance transforms, the map behind Table S4. Blue = low CoV (reproducible cells); red = high CoV (cells whose predicted movement density varies most across replicates). Values are on a 0–1 scale (CoV = 1 means the SD equals the mean); cells above 1 (about 5%–7% of the footprint) take the extreme colour.
**Figure S4:** Raw (continuous) connectivity surface, all values > 0, Combined, p07. The thresholded connectivity‐kernel map for this scenario/transform is in the main text.
**Figure S5:** Raw (continuous) connectivity surface, all values > 0, Combined, p10. The thresholded connectivity‐kernel map for this scenario/transform is in the main text.
**Figure S6:** Raw (continuous) connectivity surface, all values > 0, Combined, p15. The thresholded connectivity‐kernel map for this scenario/transform is in the main text.
**Figure S7:** Connectivity kernels (filled by study‐area complex, Viridis) and core areas (red outlines, leader‐labelled), Extant, p07. Priority ranking: Table S7. Continuous surface behind it: Figure S10.
**Figure S8:** Connectivity kernels (filled by study‐area complex, Viridis) and core areas (red outlines, leader‐labelled), Extant, p10. Priority ranking: Table S6. Continuous surface behind it: Figure S11.
**Figure S9:** Connectivity kernels (filled by study‐area complex, Viridis) and core areas (red outlines, leader‐labelled), Extant, p15. Priority ranking: Table S8. Continuous surface behind it: Figure S12.
**Figure S10:** Raw (continuous) connectivity surface, all values > 0, Extant, p07. Thresholded kernels and core areas: Figure S7.
**Figure S11:** Raw (continuous) connectivity surface, all values > 0, Extant, p10. Thresholded kernels and core areas: Figure S8.
**Figure S12:** Raw (continuous) connectivity surface, all values > 0, Extant, p15. Thresholded kernels and core areas: Figure S9.
**Figure S13:** Connectivity kernels (filled by study‐area complex, Viridis) and core areas (red outlines, leader‐labelled), Reintroduction, p07. Priority ranking: Table S10. Continuous surface behind it: Figure S16.
**Figure S14:** Connectivity kernels (filled by study‐area complex, Viridis) and core areas (red outlines, leader‐labelled), Reintroduction, p10. Priority ranking: Table S9. Continuous surface behind it: Figure S17.
**Figure S15:** Connectivity kernels (filled by study‐area complex, Viridis) and core areas (red outlines, leader‐labelled), Reintroduction, p15. Priority ranking: Table S11. Continuous surface behind it: Figure S18.
**Figure S16:** Raw (continuous) connectivity surface, all values > 0, Reintroduction, p07. Thresholded kernels and core areas: Figure S13.
**Figure S17:** Raw (continuous) connectivity surface, all values > 0, Reintroduction, p10. Thresholded kernels and core areas: Figure S14.
**Figure S18:** Raw (continuous) connectivity surface, all values > 0, Reintroduction, p15. Thresholded kernels and core areas: Figure S15.
**Figure S19:** Mean sensitivity of the priority metrics to the resistance transform, averaged over all study‐area groups. For each comparison (p10 vs. p07, p15 vs. p07, p10 vs. p15), scenario and threshold, and each metric (Extent, Kernel Sum, PAs, Intensity), the cell gives the mean symmetric relative difference (see how it is computed). Two results stand out: Intensity changes little (|srd| ≤ ~6, mostly below 3), whereas Extent and Kernel Sum scale monotonically toward the more permeable transform (p15), so the transform rescales the amount of connectivity, not its per‐area concentration or the priority ranking; and, at the Core Area threshold (*p* = 0.8), the reintroduction scenario is the most sensitive and the extant the least, whereas at the Connectivity Kernel threshold (*p* = 0.2) the three scenarios respond similarly.
**Figure S20:** Spatial confirmation for the combined scenario: cell‐wise absolute difference between the connectivity surfaces of pairs of transforms (each surface rescaled 0–1 by its own maximum; p07 vs. p10, p10 vs. p15, p07 vs. p15). Blue = agreement, red = maximum disagreement; absolute differences stay below about 0.4 and are largest within the interiors of the most extensive connectivity areas (the Dawna–Tenasserim belt, the Eastern Plains and Cardamom landscapes) rather than at patch edges.

## Data Availability

The data and R code supporting this study, including the reclassified land‐cover resistance surface and the random source‐point sets for each forest complex evaluated in the extant‐range and reintroduction scenarios, together with the full connectivity‐analysis pipeline, are openly archived on Zenodo at https://doi.org/10.5281/zenodo.18936982 (concept DOI, which always resolves to the latest version).
