# Supplementary material for: Mapping Leopard Conservation Priorities in Southeast Asia
Source: Ecol Evol. 2026 Nov 2;16(11):e74459. doi: 10.1002/ece3.74459 (PMC13727284; doi:10.1002/ece3.74459)
Supplement: Supplementary file 1 [file ECE3-16-e74459-s001.docx]

Supplementary Material to

# **Mapping Leopard Conservation Priorities in Southeast Asia**

Manuscript submitted to *Ecology and Evolution*

Atzeni Luciano^1^, Kamler Jan^1^, Ash Eric^1^, Kyaw Pyae Phyoe^2^, Rasphone Akchousanh^3^, Pin Chanratana^4,5^, Tan Cedric Kai Wei^6^, Rostro-García Susana^7^, Jantz Patrick^8^, Gonzalez Ivan^8^, Burnham Dawn^1^, Cushman Samuel A.^1,9^, Macdonald David W.^1,*^

1. Wildlife Conservation Research Unit, Department of Biology, University of Oxford, Life & Mind Building, South Parks Road, Oxford, UK

2. Wildlife Conservation Society Myanmar Program, Yangon, Myanmar

3. WWF-Laos, Vientiane, Lao PDR

4. Ministry of Environment, Cambodia

5. Conservation Ecology Program, King Mongkut's University of Technology Thonburi, Thailand

6. School of Environmental and Geographical Sciences, University of Nottingham, Malaysia

7. Panthera, 8 West 40th St, New York, NY 10018, USA

8. School of Informatics, Computing, and Cyber Systems, Northern Arizona University, Flagstaff, AZ, USA

9. Department of Biology, University of Southern Denmark, Odense, Denmark

* Corresponding author: David W. Macdonald, [david.macdonald@biology.ox.ac.uk](mailto:david.macdonald@biology.ox.ac.uk)

*Supplementary material for the connectivity analysis: sensitivity to the resistance transform, the pixel grain and the source-point replicates, and the supplementary priority maps and rankings. Everything here is supplementary; items reported in the main text are only referenced by their deposit path. Self-contained: every supplementary figure and table is embedded.*

**Contents**

1. Sensitivity tests for resistance variants and pixel grain

1.1. Resistance surfaces and transforms

1.2. Per-area Spearman correlations

1.3. Summary by comparison axis

2. Coefficient of variation across replicates

2.1. CoV summary and maps

2.2. Correlation among the global means

3. Priority connectivity kernels and core areas

3.1. Combined scenario, raw connectivity surfaces

3.2. Extant and reintroduction, priority maps

3.3. Priority ranking tables

3.4. Single-area rankings

4. Robustness of the priority ranking

4.1. Per-complex rank stability

5. Metric sensitivity to the resistance transform

## **1. Sensitivity tests for resistance variants (parametrisations) and pixel grain**

**Reported in the main text (not reproduced here):**

*Per-area Spearman table (CSV): 03_correlation/CORR_pairwise/spearman_table_SD.csv*

### **1.1 Resistance surfaces and transforms**

The connectivity model runs on a categorical land-cover resistance surface. The land-cover map is reclassified into base resistance values (Table S1), and the sensitivity to the resistance parametrisation is generated by applying three functional-form transforms to that surface (Table S2). The remainder of this section tests whether the mapped connectivity, and the resulting priority ranking, depends on this choice.

**Table S1.** Reclassification of the Miettinen et al. land-cover map of Southeast Asia into the base resistance surface. Each land-cover category (Value) is assigned a base resistance (Resistance, 1 = most permeable to 100 = most resistant): closed-forest classes = 1, regrowth / plantation / deciduous shrub / floodzone shrub = 20, mosaic / open / bare limestone = 50, water = 80, urban = 100. Three features not present in the Miettinen land-cover map are burned into this categorical input raster separately: secondary roads at resistance 30, and primary roads and built-up areas at resistance 100 (Table S2).

| **Value** | **Category** | **Resistance** |
| --- | --- | --- |
| 1 | Water | 80 |
| 2 | Mangrove | 1 |
| 3 | Peatswamp Forest | 1 |
| 4 | Lowland Evergreen Forest | 1 |
| 5 | Lower Montane Evergreen Forest | 1 |
| 6 | Upper Montane Evergreen Forest | 1 |
| 7 | Regrowth/Plantation | 20 |
| 8 | Lowland Mosaic | 50 |
| 9 | Montane Mosaic | 50 |
| 10 | Lowland Open | 50 |
| 11 | Montane Open | 50 |
| 12 | Urban | 100 |
| 13 | Large Scale Palm Plantations | 20 |
| 14 | Lowland Deciduous Forest | 1 |
| 15 | Lower Montane Deciduous Forest | 1 |
| 16 | Upper Montane Deciduous Shrub/Forest | 20 |
| 17 | Bare Limestone | 50 |
| 18 | Tonle Sap Floodzone Shrub/Forest | 20 |

*Deposit file (CSV): 01_resistance_variants/landcover_reclassification.csv*

**Table S2.** Resistance values under the three functional-form transforms. The base surface is raised to a power *p* and rescaled to 1–100 (R′ = 1 + (R*^p^* − 1) / (100*^p^* − 1) × 99), keeping the extremes fixed (minimum → 1, maximum → 100) and bending the intermediate classes: **p07** (concave, *p* = 0.7) makes intermediate classes more resistant, **p10** (linear, *p* = 1.0) is the identity, and **p15** (convex, *p* = 1.5) makes them more permeable. Each column gives the resulting integer resistance for a land-cover / feature class under that transform (the p10 column is the base value; Table S1).

| **Feature class** | **p07 (concave)** | **p10 (linear)** | **p15 (convex)** |
| --- | --- | --- | --- |
| Forest | 1 | 1 | 1 |
| Scrub / regrowth / plantation | 30 | 20 | 10 |
| Secondary road (burned in) | 41 | 30 | 17 |
| Mosaic / open / bare / agriculture | 60 | 50 | 36 |
| Water | 85 | 80 | 72 |
| Urban / built-up / primary road | 100 | 100 | 100 |

*Deposit file (CSV): 01_resistance_variants/resistance_transform_values.csv*

### **1.2 Per-area Spearman correlations**

These tests ask whether the mapped connectivity, and therefore the conservation priority ranking, depends on two analysis choices: the shape of the resistance transform (concave p0.7, linear p1.0, convex p1.5) and the pixel grain at which the surface is evaluated (~1 km vs ~500 m). For every study area the connectivity surface is correlated between choices, using the ten random source-point replicates.

**Figure S1.** Robustness of the cumulative resistant-kernel connectivity surfaces to the analysis choices, for the **extant** forest complexes. For each study area the bars give the replicate-mean Spearman rank correlation (± SD across the ten source-point replicates for the transform comparisons, and across the 30 replicate × transform combinations for the grain comparisons) between the connectivity surface computed under different choices: the resistance transforms (p0.7 vs p1.0, p1.0 vs p1.5, p0.7 vs p1.5) and the grain (1 km vs 500 m, reported at both grains). Values near 1 mean the spatial pattern is largely unaffected by the choice; lower values flag more sensitive areas.

*Figure: 03_correlation/CORR_pairwise/spearman_grouped_extant.png*

*Source data: 03_correlation/CORR_pairwise/spearman_by_area_table.csv*

*Main-text table: 03_correlation/CORR_pairwise/spearman_table_SD.csv*

**Figure S2.** Robustness of the cumulative resistant-kernel connectivity surfaces to the analysis choices, for the **reintroduction** forest complexes. For each study area the bars give the replicate-mean Spearman rank correlation (± SD across the ten source-point replicates for the transform comparisons, and across the 30 replicate × transform combinations for the grain comparisons) between the connectivity surface computed under different choices: the resistance transforms (p0.7 vs p1.0, p1.0 vs p1.5, p0.7 vs p1.5) and the grain (1 km vs 500 m, reported at both grains). Values near 1 mean the spatial pattern is largely unaffected by the choice; lower values flag more sensitive areas.

*Figure: 03_correlation/CORR_pairwise/spearman_grouped_reintroduction.png*

*Source data: 03_correlation/CORR_pairwise/spearman_by_area_table.csv*

*Main-text table: 03_correlation/CORR_pairwise/spearman_table_SD.csv*

### **1.3 Summary by comparison axis**

**Table S3.** Overall agreement of the connectivity surfaces across the analysis choices, pooled over all study areas and replicates, and for the pixel rows also over the three resistance transforms (summarising the per-area values in Figure S1 and Figure S2). Rows are the comparison axes: *pixel* = 1 km vs 500 m grain (at both grains), *resistance* = pairs of resistance transforms. For each of three coefficients, Pearson, Spearman (rank), and Pearson on log1p-transformed values (down-weighting the heavy tail of movement density), the median, minimum and maximum across all area-replicate comparisons are reported.

| **axis** | **comparison** | **pearson.median** | **pearson.min** | **pearson.max** | **spearman.median** | **spearman.min** | **spearman.max** | **pearson_log.median** | **pearson_log.min** | **pearson_log.max** |
| --- | --- | --- | --- | --- | --- | --- | --- | --- | --- | --- |
| pixel | 1km_vs_500m (1km grain) | 0.986 | 0.767 | 0.997 | 0.980 | 0.736 | 0.996 | 0.977 | 0.721 | 0.996 |
| pixel | 1km_vs_500m (500m grain) | 0.983 | 0.754 | 0.995 | 0.977 | 0.717 | 0.993 | 0.974 | 0.713 | 0.994 |
| resistance | p07 vs p10 | 0.996 | 0.934 | 0.999 | 0.993 | 0.845 | 0.998 | 0.993 | 0.910 | 1.000 |
| resistance | p07 vs p15 | 0.975 | 0.541 | 0.997 | 0.958 | 0.631 | 0.994 | 0.956 | 0.489 | 0.997 |
| resistance | p10 vs p15 | 0.990 | 0.784 | 0.999 | 0.982 | 0.787 | 0.998 | 0.981 | 0.775 | 0.999 |

*Deposit file (CSV): 03_correlation/CORR_pairwise/correlation_summary.csv*

## **2. Coefficient of variation across replicates**

### **2.1 CoV summary and maps**

This section asks how reproducible the connectivity prediction is when the source points are drawn at random: the analysis is repeated with ten independent random source-point sets and the per-cell coefficient of variation (CoV = standard deviation / mean across the ten replicate surfaces) is computed. Low CoV means the prediction barely changes between replicates.

**Table S4.** Reproducibility of the connectivity prediction across the ten random source-point replicates, per study area and for the whole landscape, for the three resistance transforms. Rows are grouped by transform (p07, p10, p15) and the groups are separated by a horizontal rule. Columns: transform = resistance transform; scope = study-area code, or GLOBAL (all areas combined per replicate); scenario = scenario the study area belongs to (extant or reintroduction; combined for GLOBAL); cov_median, cov_mean = median and mean per-cell CoV over the reachable footprint; cov_p90 = 90th percentile of the per-cell CoV (the high-variability tail); pct_cells_cov_lt0.10 = percentage of footprint cells with CoV below 0.10 (highly reproducible); n_footprint_cells = number of cells with positive mean movement density (footprint size). Lower CoV and a larger share of cells below 0.10 mean a more reproducible prediction.

| **transform** | **scope** | **scenario** | **cov_median** | **cov_mean** | **cov_p90** | **pct_cells_cov_lt0.10** | **n_footprint_cells** |
| --- | --- | --- | --- | --- | --- | --- | --- |
| p07 | GLOBAL | combined | 0.180 | 0.347 | 0.714 | 21.954 | 457,127 |
| p07 | AH | extant | 1.154 | 1.247 | 2.108 | 0 | 56 |
| p07 | CAR | reintroduction | 0.145 | 0.342 | 0.730 | 23.373 | 22,855 |
| p07 | DPKY | reintroduction | 0.139 | 0.517 | 1.930 | 0.041 | 9,776 |
| p07 | EPL | reintroduction | 0.138 | 0.301 | 0.657 | 25.924 | 32,603 |
| p07 | ER | extant | 0.091 | 0.134 | 0.210 | 53.438 | 6,544 |
| p07 | KKLT | extant | 0.158 | 0.340 | 0.773 | 32.086 | 40,579 |
| p07 | KSKS | reintroduction | 0.196 | 0.261 | 0.348 | 4.871 | 7,801 |
| p07 | MUL | extant | 0.384 | 0.581 | 1.221 | 6.308 | 26,093 |
| p07 | NEPL | reintroduction | 0.359 | 0.587 | 1.360 | 0 | 15,821 |
| p07 | NHNK | reintroduction | 0.247 | 0.388 | 0.688 | 1.378 | 18,146 |
| p07 | NN | reintroduction | 0.209 | 0.342 | 0.692 | 22.729 | 38,721 |
| p07 | NNPK | reintroduction | 0.103 | 0.278 | 0.476 | 49.069 | 6,658 |
| p07 | NPL | reintroduction | 0.400 | 0.694 | 1.769 | 0.846 | 28,259 |
| p07 | NZWS | extant | 0.111 | 0.155 | 0.222 | 43.303 | 13,334 |
| p07 | PAS | extant | 0.305 | 0.551 | 1.104 | 0 | 509 |
| p07 | PPC | extant | 0.334 | 0.529 | 1.147 | 4.669 | 13,879 |
| p07 | RBGT | extant | 0.158 | 0.313 | 0.616 | 31.443 | 21,305 |
| p07 | SAL | extant | 0.208 | 0.372 | 0.713 | 15.981 | 55,177 |
| p07 | TH | extant | 0.055 | 0.141 | 0.282 | 64.334 | 2,529 |
| p07 | TNFC | extant | 0.132 | 0.230 | 0.409 | 27.835 | 27,544 |
| p07 | UM | extant | 0.154 | 0.360 | 0.971 | 37.712 | 5,436 |
| p07 | UT | extant | 2.062 | 2.088 | 3.162 | 0 | 902 |
| p07 | VXP | reintroduction | 0.198 | 0.346 | 0.634 | 6.910 | 39,739 |
| p07 | WFCT | extant | 0.202 | 0.293 | 0.416 | 19.160 | 60,171 |
| p07 | XX | reintroduction | 0.170 | 0.247 | 0.493 | 27.044 | 28,479 |
| p10 | GLOBAL | combined | 0.174 | 0.316 | 0.587 | 23.044 | 497,867 |
| p10 | AH | extant | 1.132 | 1.226 | 2.108 | 0 | 56 |
| p10 | CAR | reintroduction | 0.139 | 0.330 | 0.693 | 27.870 | 24,611 |
| p10 | DPKY | reintroduction | 0.134 | 0.475 | 1.610 | 0.377 | 10,336 |
| p10 | EPL | reintroduction | 0.131 | 0.269 | 0.545 | 30.120 | 33,861 |
| p10 | ER | extant | 0.098 | 0.156 | 0.241 | 51.425 | 7,510 |
| p10 | KKLT | extant | 0.154 | 0.307 | 0.645 | 32.359 | 43,428 |
| p10 | KSKS | reintroduction | 0.182 | 0.243 | 0.324 | 8.168 | 8,533 |
| p10 | MUL | extant | 0.327 | 0.472 | 0.895 | 6.892 | 28,496 |
| p10 | NEPL | reintroduction | 0.297 | 0.515 | 1.166 | 0 | 19,027 |
| p10 | NHNK | reintroduction | 0.227 | 0.339 | 0.550 | 2.054 | 23,267 |
| p10 | NN | reintroduction | 0.201 | 0.324 | 0.605 | 22.539 | 41,125 |
| p10 | NNPK | reintroduction | 0.102 | 0.247 | 0.440 | 49.093 | 7,280 |
| p10 | NPL | reintroduction | 0.367 | 0.596 | 1.467 | 2.155 | 31,095 |
| p10 | NZWS | extant | 0.109 | 0.153 | 0.228 | 44.287 | 14,153 |
| p10 | PAS | extant | 0.344 | 0.667 | 2.119 | 0 | 721 |
| p10 | PPC | extant | 0.311 | 0.481 | 0.994 | 5.462 | 15,197 |
| p10 | RBGT | extant | 0.181 | 0.324 | 0.718 | 29.001 | 24,420 |
| p10 | SAL | extant | 0.204 | 0.354 | 0.611 | 19.010 | 60,706 |
| p10 | TH | extant | 0.065 | 0.152 | 0.261 | 59.190 | 3,014 |
| p10 | TNFC | extant | 0.144 | 0.233 | 0.458 | 26.138 | 30,656 |
| p10 | UM | extant | 0.187 | 0.300 | 0.543 | 32.399 | 7,028 |
| p10 | UT | extant | 1.514 | 1.726 | 3.162 | 0 | 1,158 |
| p10 | VXP | reintroduction | 0.186 | 0.309 | 0.546 | 8.789 | 43,056 |
| p10 | WFCT | extant | 0.195 | 0.283 | 0.410 | 19.311 | 63,234 |
| p10 | XX | reintroduction | 0.158 | 0.225 | 0.441 | 29.311 | 31,312 |
| p15 | GLOBAL | combined | 0.165 | 0.292 | 0.513 | 25.270 | 574,797 |
| p15 | AH | extant | 1.100 | 1.215 | 2.108 | 0 | 58 |
| p15 | CAR | reintroduction | 0.130 | 0.338 | 0.661 | 35.660 | 27,723 |
| p15 | DPKY | reintroduction | 0.133 | 0.458 | 1.434 | 15.186 | 11,517 |
| p15 | EPL | reintroduction | 0.123 | 0.245 | 0.478 | 35.002 | 36,489 |
| p15 | ER | extant | 0.120 | 0.241 | 0.421 | 41.397 | 11,165 |
| p15 | KKLT | extant | 0.145 | 0.258 | 0.504 | 34.065 | 47,949 |
| p15 | KSKS | reintroduction | 0.165 | 0.220 | 0.288 | 11.469 | 9,853 |
| p15 | MUL | extant | 0.265 | 0.384 | 0.690 | 8.323 | 32,547 |
| p15 | NEPL | reintroduction | 0.246 | 0.472 | 0.963 | 4.578 | 29,055 |
| p15 | NHNK | reintroduction | 0.203 | 0.333 | 0.585 | 5.194 | 32,169 |
| p15 | NN | reintroduction | 0.195 | 0.343 | 0.691 | 21.899 | 46,199 |
| p15 | NNPK | reintroduction | 0.120 | 0.249 | 0.544 | 45.024 | 9,113 |
| p15 | NPL | reintroduction | 0.289 | 0.461 | 1.110 | 6.741 | 35,171 |
| p15 | NZWS | extant | 0.107 | 0.151 | 0.228 | 45.438 | 15,529 |
| p15 | PAS | extant | 0.745 | 1.094 | 3.162 | 0.071 | 2,800 |
| p15 | PPC | extant | 0.287 | 0.455 | 0.949 | 6.612 | 17,469 |
| p15 | RBGT | extant | 0.184 | 0.289 | 0.531 | 27.559 | 29,569 |
| p15 | SAL | extant | 0.189 | 0.325 | 0.534 | 22.680 | 69,492 |
| p15 | TH | extant | 0.125 | 0.284 | 0.744 | 43.120 | 4,949 |
| p15 | TNFC | extant | 0.175 | 0.261 | 0.491 | 22.911 | 37,929 |
| p15 | UM | extant | 0.214 | 0.344 | 0.749 | 27.104 | 10,696 |
| p15 | UT | extant | 1.061 | 1.317 | 2.590 | 0 | 2,123 |
| p15 | VXP | reintroduction | 0.170 | 0.283 | 0.510 | 11.792 | 49,032 |
| p15 | WFCT | extant | 0.190 | 0.269 | 0.402 | 19.369 | 68,666 |
| p15 | XX | reintroduction | 0.147 | 0.222 | 0.427 | 33.720 | 36,088 |

*Deposit file (CSV): 04_COV/COV_1km/cov_raster_summary.csv*

**Figure S3.** Spatial pattern of the per-cell replicate CoV for the combined scenario under the three resistance transforms, the map behind Table S4. Blue = low CoV (reproducible cells); red = high CoV (cells whose predicted movement density varies most across replicates). Values are on a 0–1 scale (CoV = 1 means the SD equals the mean); cells above 1 (about 5–7% of the footprint) take the extreme colour.

*Figure: 04_COV/COV_1km/cov_maps_combined.png*

*Source data: 04_COV/COV_1km/mean/<surface>/global_cv.tif*

### **2.2 Correlation among the global means**

**Table S5.** Overall similarity of the three resistance transforms at the landscape scale: cell-wise correlation between the global mean connectivity surfaces (replicate mean of all areas combined). Pearson below the diagonal, Spearman rank correlation above; the diagonal is 1 by definition. Values near 1 mean the three transforms yield almost the same landscape-scale pattern.

|  | **p07** | **p10** | **p15** |
| --- | --- | --- | --- |
| p07 | 1 | 0.991 | 0.959 |
| p10 | 0.997 | 1 | 0.982 |
| p15 | 0.979 | 0.991 | 1 |

*Deposit file (CSV, Pearson): 04_COV/CORR_1km/correlation_pearson.csv*

*Deposit file (CSV, Spearman): 04_COV/CORR_1km/correlation_spearman.csv*

## **3. Priority connectivity kernels and core areas**

For every scenario (combined, extant, reintroduction) and resistance transform (p07, p10, p15) the connectivity surface is thresholded into **Connectivity Kernels** (patches above the 20th percentile of the kernel-value distribution, P = 0.2) and **Core Areas** (above the 80th percentile, P = 0.8), each tagged with the study-area complex(es) it contains and ranked by priority metrics. The combined-scenario connectivity-kernel maps and priority tables (all transforms) are in the main text; only their deposit paths are listed here.

**Reported in the main text (not reproduced here):** the combined-scenario connectivity-kernel maps and priority tables for all three transforms. Deposit files:

*Main-text figure: 05_analyses_landscapes/PRIORITY/maps/kernels_combined_p07.png*

*Main-text figure: 05_analyses_landscapes/PRIORITY/maps/kernels_combined_p10.png*

*Main-text figure: 05_analyses_landscapes/PRIORITY/maps/kernels_combined_p15.png*

*Main-text table: 05_analyses_landscapes/PRIORITY/tables/main/combined_landscape_mean_p10.csv*

*Main-text table: 05_analyses_landscapes/PRIORITY/tables/supplementary/combined_landscape_mean_p07.csv*

*Main-text table: 05_analyses_landscapes/PRIORITY/tables/supplementary/combined_landscape_mean_p15.csv*

### **3.1 Combined scenario, raw connectivity surfaces**

The continuous connectivity surfaces (all values above zero) for the combined scenario, behind the main-text connectivity-kernel maps, on the shaded-relief basemap.

**Figure S4.** Raw (continuous) connectivity surface, all values > 0, Combined, p07. The thresholded connectivity-kernel map for this scenario/transform is in the main text.

*Figure: 05_analyses_landscapes/PRIORITY/maps/raw_combined_p07.png*

*Main-text kernel map: 05_analyses_landscapes/PRIORITY/maps/kernels_combined_p07.png*

**Figure S5.** Raw (continuous) connectivity surface, all values > 0, Combined, p10. The thresholded connectivity-kernel map for this scenario/transform is in the main text.

*Figure: 05_analyses_landscapes/PRIORITY/maps/raw_combined_p10.png*

*Main-text kernel map: 05_analyses_landscapes/PRIORITY/maps/kernels_combined_p10.png*

**Figure S6.** Raw (continuous) connectivity surface, all values > 0, Combined, p15. The thresholded connectivity-kernel map for this scenario/transform is in the main text.

*Figure: 05_analyses_landscapes/PRIORITY/maps/raw_combined_p15.png*

*Main-text kernel map: 05_analyses_landscapes/PRIORITY/maps/kernels_combined_p15.png*

### **3.2 Extant and reintroduction, priority maps**

Connectivity kernels (filled by complex) and core areas (red outlines), and the continuous surfaces behind them, for the extant and reintroduction scenarios across the three transforms. Each map is cross-linked to its priority ranking table in Section 3.3.

### **Extant**

**Figure S7.** Connectivity kernels (filled by study-area complex, Viridis) and core areas (red outlines, leader-labelled), Extant, p07. Priority ranking: Table S7. Continuous surface behind it: Figure S10.

*Figure: 05_analyses_landscapes/PRIORITY/maps/kernels_extant_p07.png*

**Figure S8.** Connectivity kernels (filled by study-area complex, Viridis) and core areas (red outlines, leader-labelled), Extant, p10. Priority ranking: Table S6. Continuous surface behind it: Figure S11.

*Figure: 05_analyses_landscapes/PRIORITY/maps/kernels_extant_p10.png*

**Figure S9.** Connectivity kernels (filled by study-area complex, Viridis) and core areas (red outlines, leader-labelled), Extant, p15. Priority ranking: Table S8. Continuous surface behind it: Figure S12.

*Figure: 05_analyses_landscapes/PRIORITY/maps/kernels_extant_p15.png*

**Figure S10.** Raw (continuous) connectivity surface, all values > 0, Extant, p07. Thresholded kernels and core areas: Figure S7.

*Figure: 05_analyses_landscapes/PRIORITY/maps/raw_extant_p07.png*

**Figure S11.** Raw (continuous) connectivity surface, all values > 0, Extant, p10. Thresholded kernels and core areas: Figure S8.

*Figure: 05_analyses_landscapes/PRIORITY/maps/raw_extant_p10.png*

**Figure S12.** Raw (continuous) connectivity surface, all values > 0, Extant, p15. Thresholded kernels and core areas: Figure S9.

*Figure: 05_analyses_landscapes/PRIORITY/maps/raw_extant_p15.png*

### **Reintroduction**

**Figure S13.** Connectivity kernels (filled by study-area complex, Viridis) and core areas (red outlines, leader-labelled), Reintroduction, p07. Priority ranking: Table S10. Continuous surface behind it: Figure S16.

*Figure: 05_analyses_landscapes/PRIORITY/maps/kernels_reintroduction_p07.png*

**Figure S14.** Connectivity kernels (filled by study-area complex, Viridis) and core areas (red outlines, leader-labelled), Reintroduction, p10. Priority ranking: Table S9. Continuous surface behind it: Figure S17.

*Figure: 05_analyses_landscapes/PRIORITY/maps/kernels_reintroduction_p10.png*

**Figure S15.** Connectivity kernels (filled by study-area complex, Viridis) and core areas (red outlines, leader-labelled), Reintroduction, p15. Priority ranking: Table S11. Continuous surface behind it: Figure S18.

*Figure: 05_analyses_landscapes/PRIORITY/maps/kernels_reintroduction_p15.png*

**Figure S16.** Raw (continuous) connectivity surface, all values > 0, Reintroduction, p07. Thresholded kernels and core areas: Figure S13.

*Figure: 05_analyses_landscapes/PRIORITY/maps/raw_reintroduction_p07.png*

**Figure S17.** Raw (continuous) connectivity surface, all values > 0, Reintroduction, p10. Thresholded kernels and core areas: Figure S14.

*Figure: 05_analyses_landscapes/PRIORITY/maps/raw_reintroduction_p10.png*

**Figure S18.** Raw (continuous) connectivity surface, all values > 0, Reintroduction, p15. Thresholded kernels and core areas: Figure S15.

*Figure: 05_analyses_landscapes/PRIORITY/maps/raw_reintroduction_p15.png*

### **3.3 Priority ranking tables**

**How to read the priority tables.** In each table every row is a patch (Site = its complex[es]); the two thresholds are marked once in the **P** column (0.2 = Connectivity Kernels, 0.8 = Core Areas). Raw metrics: **Kernel Extent** = patch area (km²); **Kernel Sum** = summed kernel value in the patch; **PAs** = number of protected areas intersected; **Intensity** = Kernel Sum per km². The four “… rank” columns rescale each metric to its maximum within the block (1 = highest), and **Mean** is their average, the composite priority score used to order the patches.

### **Extant**

**Table S6.** Priority ranking, Extant, p10 resistance transform (see the column definitions). Corresponding map: Figure S8.

| **Site** | **Kernel Extent** | **Kernel Sum** | **PAs** | **Intensity** | **Kernel Extent rank** | **Kernel Sum rank** | **PAs rank** | **Intensity rank** | **Mean** | **P** |
| --- | --- | --- | --- | --- | --- | --- | --- | --- | --- | --- |
| WFCT-KKLT-MUL | 68219.870 | 1925703.700 | 32 | 28.230 | 1 | 1 | 0.550 | 1 | 0.890 | 0.2 |
| TNFC-RBGT-TH | 41593.680 | 795461.950 | 58 | 19.120 | 0.610 | 0.410 | 1 | 0.680 | 0.680 | 0.2 |
| SAL | 38291.270 | 426964.250 | 17 | 11.150 | 0.560 | 0.220 | 0.290 | 0.400 | 0.370 | 0.2 |
| UM | 4325.400 | 38118.610 | 8 | 8.810 | 0.060 | 0.020 | 0.140 | 0.310 | 0.130 | 0.2 |
| ER | 5791.520 | 39953.480 | 10 | 6.900 | 0.080 | 0.020 | 0.170 | 0.240 | 0.130 | 0.2 |
| NZWS | 11163.620 | 68100.190 | 1 | 6.100 | 0.160 | 0.040 | 0.020 | 0.220 | 0.110 | 0.2 |
| PPC | 6755.250 | 8935.180 | 3 | 1.320 | 0.100 | 0 | 0.050 | 0.050 | 0.050 | 0.2 |
| PAS | 381.120 | 412.070 | 5 | 1.080 | 0.010 | 0 | 0.090 | 0.040 | 0.030 | 0.2 |
| UT | 577.150 | 242.080 | 1 | 0.420 | 0.010 | 0 | 0.020 | 0.010 | 0.010 | 0.2 |
| WFCT | 12513.940 | 782551.300 | 11 | 62.530 | 0.940 | 1 | 1 | 1 | 0.990 | 0.8 |
| KKLT | 13272.520 | 705177.240 | 8 | 53.130 | 1 | 0.900 | 0.730 | 0.850 | 0.870 | 0.8 |
| TNFC | 8359.030 | 394017.580 | 11 | 47.140 | 0.630 | 0.500 | 1 | 0.750 | 0.720 | 0.8 |
| SAL | 6566.510 | 262638.150 | 3 | 40 | 0.490 | 0.340 | 0.270 | 0.640 | 0.440 | 0.8 |
| RBGT | 3554.960 | 116833.590 | 5 | 32.860 | 0.270 | 0.150 | 0.450 | 0.530 | 0.350 | 0.8 |

*Deposit file (CSV): 05_analyses_landscapes/PRIORITY/tables/main/extant_landscape_mean_p10.csv*

**Table S7.** Priority ranking, Extant, p07 resistance transform (see the column definitions). Corresponding map: Figure S7.

| **Site** | **Kernel Extent** | **Kernel Sum** | **PAs** | **Intensity** | **Kernel Extent rank** | **Kernel Sum rank** | **PAs rank** | **Intensity rank** | **Mean** | **P** |
| --- | --- | --- | --- | --- | --- | --- | --- | --- | --- | --- |
| WFCT-KKLT-MUL | 64395.030 | 1782494.930 | 32 | 27.680 | 1 | 1 | 1 | 1 | 1 | 0.2 |
| TNFC | 21739.140 | 515602.290 | 28 | 23.720 | 0.340 | 0.290 | 0.880 | 0.860 | 0.590 | 0.2 |
| RBGT | 15110.630 | 228615.760 | 25 | 15.130 | 0.230 | 0.130 | 0.780 | 0.550 | 0.420 | 0.2 |
| SAL | 33897.500 | 378067.660 | 14 | 11.150 | 0.530 | 0.210 | 0.440 | 0.400 | 0.390 | 0.2 |
| UM | 3502.080 | 34870.280 | 8 | 9.960 | 0.050 | 0.020 | 0.250 | 0.360 | 0.170 | 0.2 |
| ER | 5240.810 | 37618.670 | 10 | 7.180 | 0.080 | 0.020 | 0.310 | 0.260 | 0.170 | 0.2 |
| NZWS | 10441.500 | 63636.210 | 1 | 6.090 | 0.160 | 0.040 | 0.030 | 0.220 | 0.110 | 0.2 |
| TH | 1923.820 | 13210.110 | 5 | 6.870 | 0.030 | 0.010 | 0.160 | 0.250 | 0.110 | 0.2 |
| PPC | 6285.680 | 8032.590 | 3 | 1.280 | 0.100 | 0 | 0.090 | 0.050 | 0.060 | 0.2 |
| PAS | 302.700 | 332.900 | 5 | 1.100 | 0 | 0 | 0.160 | 0.040 | 0.050 | 0.2 |
| UT | 441.290 | 140.720 | 1 | 0.320 | 0.010 | 0 | 0.030 | 0.010 | 0.010 | 0.2 |
| WFCT | 11657.790 | 730647.770 | 8 | 62.670 | 0.950 | 1 | 0.730 | 1 | 0.920 | 0.8 |
| KKLT | 12321.550 | 642650.560 | 8 | 52.160 | 1 | 0.880 | 0.730 | 0.830 | 0.860 | 0.8 |
| TNFC | 7950.570 | 376183.600 | 11 | 47.320 | 0.650 | 0.510 | 1 | 0.750 | 0.730 | 0.8 |
| SAL | 5687.570 | 220705.970 | 3 | 38.800 | 0.460 | 0.300 | 0.270 | 0.620 | 0.410 | 0.8 |
| RBGT | 3197.550 | 104403.320 | 5 | 32.650 | 0.260 | 0.140 | 0.450 | 0.520 | 0.340 | 0.8 |

*Deposit file (CSV): 05_analyses_landscapes/PRIORITY/tables/supplementary/extant_landscape_mean_p07.csv*

**Table S8.** Priority ranking, Extant, p15 resistance transform (see the column definitions). Corresponding map: Figure S9.

| **Site** | **Kernel Extent** | **Kernel Sum** | **PAs** | **Intensity** | **Kernel Extent rank** | **Kernel Sum rank** | **PAs rank** | **Intensity rank** | **Mean** | **P** |
| --- | --- | --- | --- | --- | --- | --- | --- | --- | --- | --- |
| WFCT-KKLT-MUL | 75063.560 | 2198723.860 | 32 | 29.290 | 1 | 1 | 0.430 | 1 | 0.860 | 0.2 |
| TNFC-RBGT-UM-TH-UT | 54556.200 | 922065.460 | 74 | 16.900 | 0.730 | 0.420 | 1 | 0.580 | 0.680 | 0.2 |
| SAL | 44765.700 | 523687.700 | 19 | 11.700 | 0.600 | 0.240 | 0.260 | 0.400 | 0.370 | 0.2 |
| ER | 7348.800 | 44972.180 | 11 | 6.120 | 0.100 | 0.020 | 0.150 | 0.210 | 0.120 | 0.2 |
| NZWS | 12313.340 | 75501.380 | 1 | 6.130 | 0.160 | 0.030 | 0.010 | 0.210 | 0.110 | 0.2 |
| PPC | 7590.420 | 10686.440 | 3 | 1.410 | 0.100 | 0 | 0.040 | 0.050 | 0.050 | 0.2 |
| PAS | 602.670 | 612.040 | 5 | 1.020 | 0.010 | 0 | 0.070 | 0.030 | 0.030 | 0.2 |
| WFCT | 13865.170 | 870040.260 | 12 | 62.750 | 0.920 | 1 | 1 | 1 | 0.980 | 0.8 |
| KKLT | 15056.840 | 823594.680 | 8 | 54.700 | 1 | 0.950 | 0.670 | 0.870 | 0.870 | 0.8 |
| TNFC | 9195.120 | 429104.790 | 12 | 46.670 | 0.610 | 0.490 | 1 | 0.740 | 0.710 | 0.8 |
| SAL | 8198.560 | 341725.150 | 4 | 41.680 | 0.540 | 0.390 | 0.330 | 0.660 | 0.480 | 0.8 |
| RBGT | 4150.340 | 140019.360 | 5 | 33.740 | 0.280 | 0.160 | 0.420 | 0.540 | 0.350 | 0.8 |

*Deposit file (CSV): 05_analyses_landscapes/PRIORITY/tables/supplementary/extant_landscape_mean_p15.csv*

### **Reintroduction**

**Table S9.** Priority ranking, Reintroduction, p10 resistance transform (see the column definitions). Corresponding map: Figure S14.

| **Site** | **Kernel Extent** | **Kernel Sum** | **PAs** | **Intensity** | **Kernel Extent rank** | **Kernel Sum rank** | **PAs rank** | **Intensity rank** | **Mean** | **P** |
| --- | --- | --- | --- | --- | --- | --- | --- | --- | --- | --- |
| EPL-VXP-XX | 70898.630 | 1124856.230 | 35 | 15.870 | 1 | 1 | 1 | 0.600 | 0.900 | 0.2 |
| CAR | 19583.750 | 514231.680 | 12 | 26.260 | 0.280 | 0.460 | 0.340 | 1 | 0.520 | 0.2 |
| NN | 28436.020 | 318484.290 | 14 | 11.200 | 0.400 | 0.280 | 0.400 | 0.430 | 0.380 | 0.2 |
| NPL | 19173.450 | 147771.740 | 14 | 7.710 | 0.270 | 0.130 | 0.400 | 0.290 | 0.270 | 0.2 |
| DPKY | 8044.480 | 141259.910 | 6 | 17.560 | 0.110 | 0.130 | 0.170 | 0.670 | 0.270 | 0.2 |
| NNPK | 5631.040 | 94092.600 | 9 | 16.710 | 0.080 | 0.080 | 0.260 | 0.640 | 0.260 | 0.2 |
| NHNK | 14548.990 | 138438.290 | 4 | 9.520 | 0.210 | 0.120 | 0.110 | 0.360 | 0.200 | 0.2 |
| KSKS | 6710.570 | 47855.020 | 9 | 7.130 | 0.090 | 0.040 | 0.260 | 0.270 | 0.170 | 0.2 |
| NEPL | 12801.140 | 106219.670 | 2 | 8.300 | 0.180 | 0.090 | 0.060 | 0.320 | 0.160 | 0.2 |
| EPL | 12008.830 | 559136.490 | 8 | 46.560 | 1 | 1 | 1 | 1 | 1 | 0.8 |
| CAR | 9603.590 | 437942.600 | 8 | 45.600 | 0.800 | 0.780 | 1 | 0.980 | 0.890 | 0.8 |
| VXP | 7669.740 | 244491.900 | 5 | 31.880 | 0.640 | 0.440 | 0.620 | 0.680 | 0.600 | 0.8 |
| NN | 5673.880 | 233729.110 | 4 | 41.190 | 0.470 | 0.420 | 0.500 | 0.880 | 0.570 | 0.8 |
| NNPK | 2397.930 | 70984.670 | 5 | 29.600 | 0.200 | 0.130 | 0.620 | 0.640 | 0.400 | 0.8 |
| DPKY | 2461.760 | 75655.100 | 4 | 30.730 | 0.200 | 0.140 | 0.500 | 0.660 | 0.380 | 0.8 |
| NPL | 2180.020 | 63104.160 | 3 | 28.950 | 0.180 | 0.110 | 0.380 | 0.620 | 0.320 | 0.8 |
| NEPL | 1951.170 | 56426.790 | 1 | 28.920 | 0.160 | 0.100 | 0.120 | 0.620 | 0.250 | 0.8 |
| DPKY | 1259.150 | 32858.480 | 1 | 26.100 | 0.100 | 0.060 | 0.120 | 0.560 | 0.210 | 0.8 |
| NHNK | 707.530 | 17049.280 | 1 | 24.100 | 0.060 | 0.030 | 0.120 | 0.520 | 0.180 | 0.8 |
| NHNK | 450.410 | 10537.300 | 1 | 23.390 | 0.040 | 0.020 | 0.120 | 0.500 | 0.170 | 0.8 |

*Deposit file (CSV): 05_analyses_landscapes/PRIORITY/tables/main/reintroduction_landscape_mean_p10.csv*

**Table S10.** Priority ranking, Reintroduction, p07 resistance transform (see the column definitions). Corresponding map: Figure S13.

| **Site** | **Kernel Extent** | **Kernel Sum** | **PAs** | **Intensity** | **Kernel Extent rank** | **Kernel Sum rank** | **PAs rank** | **Intensity rank** | **Mean** | **P** |
| --- | --- | --- | --- | --- | --- | --- | --- | --- | --- | --- |
| EPL-VXP-XX | 66596.020 | 1013898.500 | 34 | 15.220 | 1 | 1 | 1 | 0.650 | 0.910 | 0.2 |
| CAR | 18788.690 | 440703.610 | 12 | 23.460 | 0.280 | 0.430 | 0.350 | 1 | 0.520 | 0.2 |
| NN | 26713.710 | 302771.690 | 14 | 11.330 | 0.400 | 0.300 | 0.410 | 0.480 | 0.400 | 0.2 |
| DPKY | 7420.840 | 127838.980 | 6 | 17.230 | 0.110 | 0.130 | 0.180 | 0.730 | 0.290 | 0.2 |
| NNPK | 5073.040 | 85917.810 | 9 | 16.940 | 0.080 | 0.080 | 0.260 | 0.720 | 0.290 | 0.2 |
| NPL | 17438.350 | 121828.920 | 13 | 6.990 | 0.260 | 0.120 | 0.380 | 0.300 | 0.270 | 0.2 |
| NHNK | 11538.340 | 114362.370 | 2 | 9.910 | 0.170 | 0.110 | 0.060 | 0.420 | 0.190 | 0.2 |
| KSKS | 5993.920 | 42704.190 | 9 | 7.120 | 0.090 | 0.040 | 0.260 | 0.300 | 0.180 | 0.2 |
| NEPL | 10226.320 | 82050.680 | 2 | 8.020 | 0.150 | 0.080 | 0.060 | 0.340 | 0.160 | 0.2 |
| EPL | 11429.860 | 508461.030 | 8 | 44.490 | 1 | 1 | 1 | 1 | 1 | 0.8 |
| CAR | 8992.720 | 374488.010 | 7 | 41.640 | 0.790 | 0.740 | 0.880 | 0.940 | 0.830 | 0.8 |
| NN | 5545.330 | 226403.890 | 4 | 40.830 | 0.490 | 0.450 | 0.500 | 0.920 | 0.590 | 0.8 |
| VXP | 6905.690 | 213628.320 | 5 | 30.940 | 0.600 | 0.420 | 0.620 | 0.700 | 0.590 | 0.8 |
| NNPK | 2326.820 | 67231.460 | 5 | 28.890 | 0.200 | 0.130 | 0.620 | 0.650 | 0.400 | 0.8 |
| DPKY | 2348.700 | 69132.720 | 4 | 29.430 | 0.210 | 0.140 | 0.500 | 0.660 | 0.380 | 0.8 |
| NPL | 1762.430 | 47839.500 | 3 | 27.140 | 0.150 | 0.090 | 0.380 | 0.610 | 0.310 | 0.8 |
| NEPL | 1591.030 | 43300.520 | 1 | 27.220 | 0.140 | 0.090 | 0.120 | 0.610 | 0.240 | 0.8 |
| DPKY | 1166.150 | 29051.300 | 1 | 24.910 | 0.100 | 0.060 | 0.120 | 0.560 | 0.210 | 0.8 |
| NHNK | 357.410 | 8111.730 | 1 | 22.700 | 0.030 | 0.020 | 0.120 | 0.510 | 0.170 | 0.8 |

*Deposit file (CSV): 05_analyses_landscapes/PRIORITY/tables/supplementary/reintroduction_landscape_mean_p07.csv*

**Table S11.** Priority ranking, Reintroduction, p15 resistance transform (see the column definitions). Corresponding map: Figure S15.

| **Site** | **Kernel Extent** | **Kernel Sum** | **PAs** | **Intensity** | **Kernel Extent rank** | **Kernel Sum rank** | **PAs rank** | **Intensity rank** | **Mean** | **P** |
| --- | --- | --- | --- | --- | --- | --- | --- | --- | --- | --- |
| EPL-NPL-VXP-XX | 101613.180 | 1534692.330 | 50 | 15.100 | 1 | 1 | 1 | 0.500 | 0.870 | 0.2 |
| CAR | 21554.080 | 651318.410 | 13 | 30.220 | 0.210 | 0.420 | 0.260 | 1 | 0.470 | 0.2 |
| NN | 31656.380 | 346017.740 | 14 | 10.930 | 0.310 | 0.230 | 0.280 | 0.360 | 0.290 | 0.2 |
| DPKY | 8830.420 | 166448.760 | 6 | 18.850 | 0.090 | 0.110 | 0.120 | 0.620 | 0.230 | 0.2 |
| NNPK | 6560.130 | 108632.240 | 10 | 16.560 | 0.060 | 0.070 | 0.200 | 0.550 | 0.220 | 0.2 |
| NHNK | 21374.440 | 193077.840 | 4 | 9.030 | 0.210 | 0.130 | 0.080 | 0.300 | 0.180 | 0.2 |
| NEPL | 17503.100 | 173233.810 | 2 | 9.900 | 0.170 | 0.110 | 0.040 | 0.330 | 0.160 | 0.2 |
| KSKS | 7782.800 | 56818.850 | 9 | 7.300 | 0.080 | 0.040 | 0.180 | 0.240 | 0.130 | 0.2 |
| EPL | 13037.290 | 653767.740 | 9 | 50.150 | 1 | 1 | 1 | 0.980 | 1 | 0.8 |
| CAR | 11050.560 | 565118.510 | 8 | 51.140 | 0.850 | 0.860 | 0.890 | 1 | 0.900 | 0.8 |
| VXP | 8885.120 | 298691.150 | 5 | 33.620 | 0.680 | 0.460 | 0.560 | 0.660 | 0.590 | 0.8 |
| NN | 5861.700 | 244887.070 | 4 | 41.780 | 0.450 | 0.370 | 0.440 | 0.820 | 0.520 | 0.8 |
| NNPK | 2508.260 | 76649.740 | 6 | 30.560 | 0.190 | 0.120 | 0.670 | 0.600 | 0.390 | 0.8 |
| NPL | 3172.930 | 100822.970 | 4 | 31.780 | 0.240 | 0.150 | 0.440 | 0.620 | 0.370 | 0.8 |
| DPKY | 2694.260 | 88536.310 | 4 | 32.860 | 0.210 | 0.140 | 0.440 | 0.640 | 0.360 | 0.8 |
| NEPL | 2835.580 | 92431.200 | 1 | 32.600 | 0.220 | 0.140 | 0.110 | 0.640 | 0.280 | 0.8 |
| NHNK | 2585.760 | 68802.690 | 2 | 26.610 | 0.200 | 0.110 | 0.220 | 0.520 | 0.260 | 0.8 |
| DPKY | 1499.860 | 42330.150 | 1 | 28.220 | 0.120 | 0.060 | 0.110 | 0.550 | 0.210 | 0.8 |

*Deposit file (CSV): 05_analyses_landscapes/PRIORITY/tables/supplementary/reintroduction_landscape_mean_p15.csv*

### **3.4 Single-area rankings**

The priority metrics computed for each forest complex in isolation (single-area rankings) are provided in the deposit but are not reproduced here. Deposit files:

*Deposit file: 05_analyses_landscapes/PRIORITY/tables/supplementary/extant_single_area_mean_p07.csv*

*Deposit file: 05_analyses_landscapes/PRIORITY/tables/supplementary/extant_single_area_mean_p10.csv*

*Deposit file: 05_analyses_landscapes/PRIORITY/tables/supplementary/extant_single_area_mean_p15.csv*

*Deposit file: 05_analyses_landscapes/PRIORITY/tables/supplementary/reintroduction_single_area_mean_p07.csv*

*Deposit file: 05_analyses_landscapes/PRIORITY/tables/supplementary/reintroduction_single_area_mean_p10.csv*

*Deposit file: 05_analyses_landscapes/PRIORITY/tables/supplementary/reintroduction_single_area_mean_p15.csv*

## **4. Robustness of the priority ranking across parameterisations**

How stable the priority ranking is across the three resistance transforms is quantified with Kendall's coefficient of concordance (W) and, per study-area complex, the spread of its rank. The concordance table, the two main rank figures (the rank heat map and the rank-stability figure) and the multi-forest metric-change figure are reported in the main text; the per-complex rank-stability table is reproduced here.

**Reported in the main text (not reproduced here):**

*Concordance table (CSV): 05_analyses_landscapes/PRIORITY/robustness/rank_concordance_formatted.csv*

*Rank heat map: 05_analyses_landscapes/PRIORITY/robustness/rank_heatmap_combined.png*

*Rank-stability figure: 05_analyses_landscapes/PRIORITY/robustness/rank_stability_main.png*

*Multi-forest metric-change figure: 05_analyses_landscapes/PRIORITY/robustness/multiforest_pairwise.png*

*Multi-forest figure, data (CSV): 05_analyses_landscapes/PRIORITY/robustness/multiforest_pairwise.csv*

### **4.1 Per-complex rank stability**

**Table S12.** Rank stability of each study-area complex across the three resistance transforms, by scenario, level (Landscape = complexes ranked on the combined-area surface; Single area = each complex ranked in isolation) and threshold P (0.2 = Connectivity Kernels, 0.8 = Core Areas). **Mean rank** = the complex's mean priority rank across the three transforms; **SD** = its standard deviation; **Min**/**Max** = its best and worst rank across the three transforms. At the Landscape level a complex takes, under each transform, the best (lowest) rank of any patch it belongs to, so its Min–Max range spans from the rank it holds when it forms a standalone patch to the higher rank it inherits when it coalesces into a multi-forest cluster; this is why peripheral areas such as NPL, UM, UT and TH show a wide range at P = 0.2, where under the more permeable transforms they merge into a high-priority cluster. A complex that forms no patch above the threshold under a given transform is counted immediately below the last ranked patch of that transform, so mean ranks and ranges for complexes that qualify under only some of the transforms mix actual and placeholder positions: at P = 0.8 in the combined scenario this applies to MUL and NHNK, which cross the 80th percentile only under p1.5. A small SD and a narrow Min–Max range indicate a ranking that is stable to the resistance transform.

| **Scenario** | **Level** | **P** | **Complex** | **Mean rank** | **SD** | **Min** | **Max** |
| --- | --- | --- | --- | --- | --- | --- | --- |
| Extant | Landscape | 0.2 | ER | 5.00 | 1.00 | 4 | 6 |
| Extant | Landscape | 0.2 | KKLT | 1.00 | 0.00 | 1 | 1 |
| Extant | Landscape | 0.2 | MUL | 1.00 | 0.00 | 1 | 1 |
| Extant | Landscape | 0.2 | NZWS | 6.00 | 1.00 | 5 | 7 |
| Extant | Landscape | 0.2 | PAS | 8.33 | 1.53 | 7 | 10 |
| Extant | Landscape | 0.2 | PPC | 7.33 | 1.53 | 6 | 9 |
| Extant | Landscape | 0.2 | RBGT | 2.33 | 0.58 | 2 | 3 |
| Extant | Landscape | 0.2 | SAL | 3.33 | 0.58 | 3 | 4 |
| Extant | Landscape | 0.2 | TH | 4.00 | 3.46 | 2 | 8 |
| Extant | Landscape | 0.2 | TNFC | 2.00 | 0.00 | 2 | 2 |
| Extant | Landscape | 0.2 | UM | 3.67 | 1.53 | 2 | 5 |
| Extant | Landscape | 0.2 | UT | 7.33 | 4.73 | 2 | 11 |
| Extant | Landscape | 0.2 | WFCT | 1.00 | 0.00 | 1 | 1 |
| Extant | Landscape | 0.8 | KKLT | 2.00 | 0.00 | 2 | 2 |
| Extant | Landscape | 0.8 | RBGT | 5.00 | 0.00 | 5 | 5 |
| Extant | Landscape | 0.8 | SAL | 4.00 | 0.00 | 4 | 4 |
| Extant | Landscape | 0.8 | TNFC | 3.00 | 0.00 | 3 | 3 |
| Extant | Landscape | 0.8 | WFCT | 1.00 | 0.00 | 1 | 1 |
| Extant | Single area | 0.2 | ER | 7.00 | 0.00 | 7 | 7 |
| Extant | Single area | 0.2 | KKLT | 3.00 | 0.00 | 3 | 3 |
| Extant | Single area | 0.2 | MUL | 6.00 | 0.00 | 6 | 6 |
| Extant | Single area | 0.2 | NZWS | 9.33 | 0.58 | 9 | 10 |
| Extant | Single area | 0.2 | PAS | 11.00 | 1.73 | 9 | 12 |
| Extant | Single area | 0.2 | PPC | 11.33 | 0.58 | 11 | 12 |
| Extant | Single area | 0.2 | RBGT | 5.00 | 0.00 | 5 | 5 |
| Extant | Single area | 0.2 | SAL | 4.00 | 0.00 | 4 | 4 |
| Extant | Single area | 0.2 | TH | 10.33 | 0.58 | 10 | 11 |
| Extant | Single area | 0.2 | TNFC | 2.00 | 0.00 | 2 | 2 |
| Extant | Single area | 0.2 | UM | 8.00 | 0.00 | 8 | 8 |
| Extant | Single area | 0.2 | UT | 13.00 | 0.00 | 13 | 13 |
| Extant | Single area | 0.2 | WFCT | 1.00 | 0.00 | 1 | 1 |
| Extant | Single area | 0.8 | ER | 7.67 | 0.58 | 7 | 8 |
| Extant | Single area | 0.8 | KKLT | 2.00 | 0.00 | 2 | 2 |
| Extant | Single area | 0.8 | MUL | 6.00 | 0.00 | 6 | 6 |
| Extant | Single area | 0.8 | NZWS | 10.67 | 0.58 | 10 | 11 |
| Extant | Single area | 0.8 | PAS | 12.33 | 0.58 | 12 | 13 |
| Extant | Single area | 0.8 | PPC | 9.33 | 1.15 | 8 | 10 |
| Extant | Single area | 0.8 | RBGT | 5.00 | 0.00 | 5 | 5 |
| Extant | Single area | 0.8 | SAL | 4.00 | 0.00 | 4 | 4 |
| Extant | Single area | 0.8 | TH | 9.67 | 1.15 | 9 | 11 |
| Extant | Single area | 0.8 | TNFC | 3.00 | 0.00 | 3 | 3 |
| Extant | Single area | 0.8 | UM | 7.67 | 1.15 | 7 | 9 |
| Extant | Single area | 0.8 | UT | 12.33 | 0.58 | 12 | 13 |
| Extant | Single area | 0.8 | WFCT | 1.00 | 0.00 | 1 | 1 |
| Reintroduction | Landscape | 0.2 | CAR | 2.00 | 0.00 | 2 | 2 |
| Reintroduction | Landscape | 0.2 | DPKY | 4.33 | 0.58 | 4 | 5 |
| Reintroduction | Landscape | 0.2 | EPL | 1.00 | 0.00 | 1 | 1 |
| Reintroduction | Landscape | 0.2 | KSKS | 8.00 | 0.00 | 8 | 8 |
| Reintroduction | Landscape | 0.2 | NEPL | 8.33 | 1.15 | 7 | 9 |
| Reintroduction | Landscape | 0.2 | NHNK | 6.67 | 0.58 | 6 | 7 |
| Reintroduction | Landscape | 0.2 | NN | 3.00 | 0.00 | 3 | 3 |
| Reintroduction | Landscape | 0.2 | NNPK | 5.33 | 0.58 | 5 | 6 |
| Reintroduction | Landscape | 0.2 | NPL | 3.67 | 2.52 | 1 | 6 |
| Reintroduction | Landscape | 0.2 | VXP | 1.00 | 0.00 | 1 | 1 |
| Reintroduction | Landscape | 0.2 | XX | 1.00 | 0.00 | 1 | 1 |
| Reintroduction | Landscape | 0.8 | CAR | 2.00 | 0.00 | 2 | 2 |
| Reintroduction | Landscape | 0.8 | DPKY | 6.33 | 0.58 | 6 | 7 |
| Reintroduction | Landscape | 0.8 | EPL | 1.00 | 0.00 | 1 | 1 |
| Reintroduction | Landscape | 0.8 | NEPL | 8.00 | 0.00 | 8 | 8 |
| Reintroduction | Landscape | 0.8 | NHNK | 9.67 | 0.58 | 9 | 10 |
| Reintroduction | Landscape | 0.8 | NN | 3.67 | 0.58 | 3 | 4 |
| Reintroduction | Landscape | 0.8 | NNPK | 5.00 | 0.00 | 5 | 5 |
| Reintroduction | Landscape | 0.8 | NPL | 6.67 | 0.58 | 6 | 7 |
| Reintroduction | Landscape | 0.8 | VXP | 3.33 | 0.58 | 3 | 4 |
| Reintroduction | Single area | 0.2 | CAR | 2.00 | 0.00 | 2 | 2 |
| Reintroduction | Single area | 0.2 | DPKY | 8.33 | 0.58 | 8 | 9 |
| Reintroduction | Single area | 0.2 | EPL | 1.00 | 0.00 | 1 | 1 |
| Reintroduction | Single area | 0.2 | KSKS | 10.33 | 0.58 | 10 | 11 |
| Reintroduction | Single area | 0.2 | NEPL | 10.67 | 0.58 | 10 | 11 |
| Reintroduction | Single area | 0.2 | NHNK | 8.33 | 1.15 | 7 | 9 |
| Reintroduction | Single area | 0.2 | NN | 3.00 | 0.00 | 3 | 3 |
| Reintroduction | Single area | 0.2 | NNPK | 7.33 | 0.58 | 7 | 8 |
| Reintroduction | Single area | 0.2 | NPL | 5.00 | 0.00 | 5 | 5 |
| Reintroduction | Single area | 0.2 | VXP | 4.00 | 0.00 | 4 | 4 |
| Reintroduction | Single area | 0.2 | XX | 6.00 | 0.00 | 6 | 6 |
| Reintroduction | Single area | 0.8 | CAR | 3.67 | 0.58 | 3 | 4 |
| Reintroduction | Single area | 0.8 | DPKY | 7.33 | 1.53 | 6 | 9 |
| Reintroduction | Single area | 0.8 | EPL | 1.00 | 0.00 | 1 | 1 |
| Reintroduction | Single area | 0.8 | KSKS | 10.00 | 1.00 | 9 | 11 |
| Reintroduction | Single area | 0.8 | NEPL | 10.67 | 0.58 | 10 | 11 |
| Reintroduction | Single area | 0.8 | NHNK | 8.67 | 1.53 | 7 | 10 |
| Reintroduction | Single area | 0.8 | NN | 2.00 | 0.00 | 2 | 2 |
| Reintroduction | Single area | 0.8 | NNPK | 7.33 | 1.15 | 6 | 8 |
| Reintroduction | Single area | 0.8 | NPL | 5.00 | 0.00 | 5 | 5 |
| Reintroduction | Single area | 0.8 | VXP | 3.33 | 0.58 | 3 | 4 |
| Reintroduction | Single area | 0.8 | XX | 7.00 | 1.00 | 6 | 8 |
| Combined | Landscape | 0.2 | CAR | 4.00 | 0.00 | 4 | 4 |
| Combined | Landscape | 0.2 | DPKY | 7.67 | 1.15 | 7 | 9 |
| Combined | Landscape | 0.2 | EPL | 2.00 | 0.00 | 2 | 2 |
| Combined | Landscape | 0.2 | ER | 12.67 | 1.15 | 12 | 14 |
| Combined | Landscape | 0.2 | KKLT | 1.00 | 0.00 | 1 | 1 |
| Combined | Landscape | 0.2 | KSKS | 12.67 | 1.53 | 11 | 14 |
| Combined | Landscape | 0.2 | MUL | 1.00 | 0.00 | 1 | 1 |
| Combined | Landscape | 0.2 | NEPL | 12.00 | 2.65 | 10 | 15 |
| Combined | Landscape | 0.2 | NHNK | 10.67 | 2.08 | 9 | 13 |
| Combined | Landscape | 0.2 | NN | 6.33 | 0.58 | 6 | 7 |
| Combined | Landscape | 0.2 | NNPK | 8.00 | 0.00 | 8 | 8 |
| Combined | Landscape | 0.2 | NPL | 7.00 | 4.36 | 2 | 10 |
| Combined | Landscape | 0.2 | NZWS | 14.67 | 1.53 | 13 | 16 |
| Combined | Landscape | 0.2 | PAS | 17.00 | 2.00 | 15 | 19 |
| Combined | Landscape | 0.2 | PPC | 16.00 | 2.00 | 14 | 18 |
| Combined | Landscape | 0.2 | RBGT | 3.67 | 1.15 | 3 | 5 |
| Combined | Landscape | 0.2 | SAL | 5.33 | 0.58 | 5 | 6 |
| Combined | Landscape | 0.2 | TH | 7.67 | 8.08 | 3 | 17 |
| Combined | Landscape | 0.2 | TNFC | 3.00 | 0.00 | 3 | 3 |
| Combined | Landscape | 0.2 | UM | 8.67 | 4.93 | 3 | 12 |
| Combined | Landscape | 0.2 | UT | 13.67 | 9.29 | 3 | 20 |
| Combined | Landscape | 0.2 | VXP | 2.00 | 0.00 | 2 | 2 |
| Combined | Landscape | 0.2 | WFCT | 1.00 | 0.00 | 1 | 1 |
| Combined | Landscape | 0.2 | XX | 2.00 | 0.00 | 2 | 2 |
| Combined | Landscape | 0.8 | CAR | 5.00 | 0.00 | 5 | 5 |
| Combined | Landscape | 0.8 | DPKY | 11.00 | 0.00 | 11 | 11 |
| Combined | Landscape | 0.8 | EPL | 3.00 | 0.00 | 3 | 3 |
| Combined | Landscape | 0.8 | KKLT | 2.00 | 0.00 | 2 | 2 |
| Combined | Landscape | 0.8 | MUL | 15.67 | 1.15 | 15 | 17 |
| Combined | Landscape | 0.8 | NEPL | 13.00 | 0.00 | 13 | 13 |
| Combined | Landscape | 0.8 | NHNK | 15.00 | 0.00 | 15 | 15 |
| Combined | Landscape | 0.8 | NN | 8.00 | 0.00 | 8 | 8 |
| Combined | Landscape | 0.8 | NNPK | 10.00 | 0.00 | 10 | 10 |
| Combined | Landscape | 0.8 | NPL | 12.00 | 0.00 | 12 | 12 |
| Combined | Landscape | 0.8 | RBGT | 9.00 | 0.00 | 9 | 9 |
| Combined | Landscape | 0.8 | SAL | 6.00 | 0.00 | 6 | 6 |
| Combined | Landscape | 0.8 | TNFC | 4.00 | 0.00 | 4 | 4 |
| Combined | Landscape | 0.8 | VXP | 7.00 | 0.00 | 7 | 7 |
| Combined | Landscape | 0.8 | WFCT | 1.00 | 0.00 | 1 | 1 |

*Deposit file (CSV): 05_analyses_landscapes/PRIORITY/robustness/rank_stability_formatted.csv*

## **5. Metric sensitivity to the resistance transform**

**How the percentages are computed.** For every pairwise comparison of two resistance transforms (A vs B), every study-area complex and every metric, the metric total under A and under B is combined as the *symmetric relative difference* srd = 100·(A−B)/(A+B). It treats A and B symmetrically (swapping them only flips the sign) and is bounded to ±100, so it stays stable when A+B is small, unlike an ordinary per cent change, 100·(A−B)/B, which blows up when the denominator is small. The same complex can stand alone as its own patch under one transform but fall inside a multi-forest cluster under another (and vice versa), so a lone patch on one side would otherwise be compared with a whole cluster on the other. To keep every comparison like-for-like on a fixed unit, any areas that share a patch under either transform are grouped together (connected components), and on each side the metric is totalled over every patch that contains any of the group's areas. Extent, Kernel Sum and PAs are additive and are simply summed over those patches; Intensity is a ratio (kernel value per km²) and cannot be summed, so it is re-derived from the aggregated totals as summed Kernel Sum ÷ summed Extent. The group is defined on the union of the two transforms, so an area that forms a patch under only one of them (for example a small forest that clears the threshold at p15 but not at p07) adds to that side's total and contributes nothing to the other, which is what drives the additive metrics (Extent, Kernel Sum) upward while Intensity stays almost flat because its numerator and denominator grow together. Each srd therefore compares the same union of forest areas under the two transforms. The figure below averages this over all study-area groups. Note that a value of e.g. +14 corresponds to about a +33% ordinary change (A/B = 1.14/0.86); the approximation srd ≈ ½ the per cent change holds only for small differences.

**Figure S19.** Mean sensitivity of the priority metrics to the resistance transform, averaged over all study-area groups. For each comparison (p10 vs p07, p15 vs p07, p10 vs p15), scenario and threshold, and each metric (Extent, Kernel Sum, PAs, Intensity), the cell gives the mean symmetric relative difference (see how it is computed). Two results stand out: **Intensity changes little** (|srd| ≤ ~6, mostly below 3), whereas Extent and Kernel Sum scale monotonically toward the more permeable transform (p15), so the transform rescales the amount of connectivity, not its per-area concentration or the priority ranking; and, at the Core Area threshold (P = 0.8), the **reintroduction scenario is the most sensitive and the extant the least**, whereas at the Connectivity Kernel threshold (P = 0.2) the three scenarios respond similarly.

*Figure: 05_analyses_landscapes/PRIORITY/differences/merge_delta_summary.png*

*Source data (CSV): 05_analyses_landscapes/PRIORITY/differences/merge_delta_summary.csv*

**Figure S20.** Spatial confirmation for the combined scenario: cell-wise absolute difference between the connectivity surfaces of pairs of transforms (each surface rescaled 0–1 by its own maximum; p07 vs p10, p10 vs p15, p07 vs p15). Blue = agreement, red = maximum disagreement; absolute differences stay below about 0.4 and are largest within the interiors of the most extensive connectivity areas (the Dawna–Tenasserim belt, the Eastern Plains and Cardamom landscapes) rather than at patch edges.

*Figure: 05_analyses_landscapes/PRIORITY/differences/diff_resistance.png*

*Source data (GeoTIFFs): 05_analyses_landscapes/PRIORITY/differences/diff_*.tif*

**Not reproduced here (deposit only), as they repeat the summary above:**

***Study-area level*** *(each complex shown individually, taking its group's relative change; the grouping and summing rules above apply in full):*

*Detailed heat map, combined, P = 0.2: 05_analyses_landscapes/PRIORITY/differences/merge_delta_combined_P20.png*

*Detailed heat map, combined, P = 0.8: 05_analyses_landscapes/PRIORITY/differences/merge_delta_combined_P80.png*

*Detailed heat map, extant, P = 0.2: 05_analyses_landscapes/PRIORITY/differences/merge_delta_extant_P20.png*

*Detailed heat map, extant, P = 0.8: 05_analyses_landscapes/PRIORITY/differences/merge_delta_extant_P80.png*

*Detailed heat map, reintroduction, P = 0.2: 05_analyses_landscapes/PRIORITY/differences/merge_delta_reintroduction_P20.png*

*Detailed heat map, reintroduction, P = 0.8: 05_analyses_landscapes/PRIORITY/differences/merge_delta_reintroduction_P80.png*

*Full per-group data (CSV): 05_analyses_landscapes/PRIORITY/differences/merge_delta_pairwise.csv*

***Scenario level*** *(mean metric value on each side of a comparison, averaged over all groups in a scenario, not per area; the grouping/summing rule enters only in forming those per-group values):*

*Dumbbell, P = 0.2: 05_analyses_landscapes/PRIORITY/differences/merge_delta_dumbbell_P20.png*

*Dumbbell, P = 0.8: 05_analyses_landscapes/PRIORITY/differences/merge_delta_dumbbell_P80.png*
